# Exploring the cobia (*Rachycentron canadum*) genome: unveiling putative male heterogametic regions and identification of sex-specific markers

**DOI:** 10.1093/gigascience/giae034

**Published:** 2024-07-12

**Authors:** Xueyan Shen, Jie Hu, José M Yáñez, Giana Bastos Gomes, Zhi Weng Josiah Poon, Derick Foster, Jorge F Alarcon, Libin Shao, Xinyu Guo, Yunchang Shao, Roger Huerlimann, Chengze Li, Evan Goulden, Kelli Anderson, Guangyi Fan, Jose A Domingos

**Affiliations:** Tropical Futures Institute, James Cook University Singapore, 387380, Singapore; BGI-Qingdao, BGI-Shenzhen, Qingdao, Shandong 266555, China; Facultad de Ciencias Veterinarias y Pecuarias, Universidad de Chile, 8820808 Santiago, Chile; Temasek Life Sciences Laboratory, 1 Research Link, National University of Singapore, 117604, Singapore; James Cook University, 387380, Singapore; Open Blue Sea Farms, Panama City, Panama; Open Blue Sea Farms, Panama City, Panama; BGI-Qingdao, BGI-Shenzhen, Qingdao, Shandong 266555, China; BGI-Qingdao, BGI-Shenzhen, Qingdao, Shandong 266555, China; China National GeneBank, BGI-Shenzhen, Shenzhen, Guangdong 518120, China; State Key Laboratory of Quality Research in Chinese Medicine, Institute of Chinese Medical Sciences, University of Macau, Macao 999078, China; Geogia Tech Shenzhen Institute (GTSI), Tianjin University, Shen Zhen 518067, China; Marine Climate Change Unit, Okinawa Institute of Science and Technology (OIST), Okinawa, 904-0495, Japan; Marine Climate Change Unit, Okinawa Institute of Science and Technology (OIST), Okinawa, 904-0495, Japan; Department of Agriculture and Fisheries, Queensland Government, Bribie Island Research Centre, Woorim, QLD 4507, Australia; Department of Agriculture and Fisheries, Queensland Government, Bribie Island Research Centre, Woorim, QLD 4507, Australia; BGI-Qingdao, BGI-Shenzhen, Qingdao, Shandong 266555, China; China National GeneBank, BGI-Shenzhen, Shenzhen, Guangdong 518120, China; BGI-Shenzhen, Shenzhen, Guangdong 518083, China; Tropical Futures Institute, James Cook University Singapore, 387380, Singapore; Centre for Sustainable Tropical Fisheries and Aquaculture, James Cook University, Townsville QLD 4811, Australia

**Keywords:** chromosome-level genome, cobia, molecular sex markers, stLFR, Hi-C, PacBio sequencing

## Abstract

**Background:**

Cobia (*Rachycentron canadum*) is the only member of the Rachycentridae family and exhibits considerable sexual dimorphism in growth rate. Sex determination in teleosts has been a long-standing basic biological question, and the molecular mechanisms of sex determination/differentiation in cobia are completely unknown.

**Results:**

Here, we reported 2 high-quality, chromosome-level annotated male and female cobia genomes with assembly sizes of 586.51 Mb (contig/scaffold N50: 86.0 kb/24.3 Mb) and 583.88 Mb (79.9 kb/22.5 Mb), respectively. Synteny inference among perciform genomes revealed that cobia and the remora *Echeneis naucrates* were sister groups. Further, whole-genome resequencing of 31 males and 60 females, genome-wide association study, and sequencing depth analysis identified 3 short male-specific regions within a 10.7-kb continuous genomic region on male chromosome 18, which hinted at an undifferentiated sex chromosome system with a putative XX/XY mode of sex determination in cobia. Importantly, the only 2 genes within/between the male-specific regions, epoxide hydrolase 1 (*ephx1*, renamed *cephx1y*) and transcription factor 24 (*tcf24*, renamed *ctcf24y*), showed testis-specific/biased gene expression, whereas their counterparts *cephx1x* and *ctf24x*, located in female chromosome 18, were similarly expressed in both sexes. In addition, male-specific PCR targeting the *cephx1y* gene revealed that this genomic feature is conserved in cobia populations from Panama, Brazil, Australia, and Japan.

**Conclusion:**

The first comprehensive genomic survey presented here is a valuable resource for future studies on cobia population structure and dynamics, conservation, and evolutionary history. Furthermore, it establishes evidence of putative male heterogametic regions with 2 genes playing a potential role in the sex determination of the species, and it provides further support for the rapid evolution of sex-determining mechanisms in teleost fish.

## Introduction

Cobia (*Rachycentron canadum*) is a large migratory pelagic fish with geographic distribution in tropical and subtropical waters worldwide with the exception of the eastern Pacific Ocean [1]. It is a promising marine fish species with great aquaculture potential due to its desirable traits, such as excellent quality fillets, easy adaptation to captivity, high survival rates, tolerance to variations in temperature and salinity, and high growth rate [2]. The species has been farmed in many countries around the world, including China, Taiwan, and Hong Kong, and more recently expanding to Australia, Vietnam, and the American continent (United States, Brazil, Panama, Belize, etc.) [3–5]. Cobia exhibits a strong sexually dimorphic growth [6–8]. Females grow faster than males in both body length and weight, creating considerable differences between sexes. At similar developmental stages, females can be double the size of males [1]. In light of this, it is widely acknowledged that monosex female breeding through artificial sex control can significantly boost cobia aquaculture yields [2]. The morphological characteristics (i.e., secondary sexual traits) used for sexing are usually only observed after sexual maturation and thus are not useful for sexing juvenile fish. Nevertheless, it is often useful to know the sex of juveniles (e.g., in aquaculture breeding programs). Relying solely on cobia morphology is not enough to distinguish their sex at any developmental stage, including after sexual maturity. Furthermore, a reliable approach for distinguishing cobia’s genotypic sex has yet to be established.

Teleost fish exhibit a remarkable diversity and complexity of sex-determining mechanisms, and sex differentiation involves the expression of a considerable number of genes in a spatial and temporal order [9]. Sexual determination mechanisms in fish may involve genetic control (e.g., heterogamety for males (XY) or females (ZW)), multiple sex-determining chromosomes and genes (X1 × 1 × 2 × 2/X1 × 2Y, XX/XY1Y2), environmental triggers (e.g., temperature, pH, behavior, population density, and social status) [10–12], epigenetic sex determination, and hermaphroditism [13–16]. Currently, multiple master sex determination genes have been reported in various fish species (for review, see [17]), such as *sdy* in rainbow trout (*Oncorhynchus mykiss*) [18], *dmy/dmrt1* in Japanese rice fish/medaka (*Oryzias latipes*) [19, 20], *amhy/amhby* in Patagonian pejerrey (*Odontesthes hatcheri*) [21], Nile tilapia (*Oreochromis niloticus*) [22], three-spined stickleback [23, 24] and northern pike (*Esox lucius*) [25], *hsd17b1* in yellowtail spp. [26], and *bcar1* in channel catfish (*Ictalurus punctatus*) [27].

The even representation of males and females within cobia populations suggests that there is a genetic system (i.e., a master gene) driving sex determination, and the species is considered gonochoristic [1]. Rare occurrences of intersex individuals have been reported in India [28] and Australia [3], with the latter supposedly attributed to the presence of endocrine-disrupting compounds in the water. Unfortunately, limited knowledge is available on the molecular mechanisms of sex determination and differentiation in this species. To date, there have been no reported sex chromosomes, sex-determining regions, or sex determination genes in cobia. Furthermore, cytologically there are no distinguishable sex chromosomes observed between genders, as male and female cobia show the same diploid number (2n = 48) and the same karyotype morphology [2, 29]. Hence, the lack of reliable genotypic and phenotypic approaches for distinguishing the sex of cobia presents a significant hurdle for practitioners seeking to optimize broodstock management, conduct molecular selective breeding, and advance the conservation of the species. Consequently, it is crucial to explore the genetic underprinnings of sex determination and develop molecular markers that permit noninvasive and early sexing of cobia individuals.

Cobia is the extant monotypic member of family Rachycentridae, order Carangiformes, which consists of 6 families. Three of these families (i.e., Rachycentridae, Coryphaenidae, and Echeneidae) are within the super family Echeneoidea that comprise a monophyletic grouping [30, 31]. *R. canadum* was assumed to be closely related (sister groups) to the remoras (*Echeneis naucrates*), within the family Echeneidae, based on the morphology (form, color, and fin shape) of juveniles [31]. However, osteological examinations revealed a greater likelihood of sister groups between *R. canadum* and *Coryphaena* based on the larval morphology [31]. In addition, a phylogenetic analysis of 138 putatively informative characters of 11 species (including *R. canadum*) resulted in a single most parsimonious tree and showed that Rachycentridae is the sister-group to Echeneidae [32]. Phylogenetics of *Carangoides* based on the complete mitochondrial DNA, however, supported that the relationship between *R. canadum* and mahi-mahi (*Coryphaena hippurus*), within family Coryphaenidae, was the closest [33]. Although these studies have shed light on *R. canadum* phylogeny in relation to other clades, whether it is more closely related to Coryphaenidae or Echeneidae still remains controversial. Therefore, further studies are required to elucidate the phylogenetic relationships of *R. canadum* within the order Carangiformes and understand its evolutionary history.

Genomic resources for cobia are currently extremely limited, hindering a better understanding of the genetic basis of sex determination and differentiation, as well as the molecular mechanisms of remarkable sexual dimorphisms in this unique fish species. Therefore, the goals of this study were to (i) assemble the first male and female chromosome-level reference genome for cobia; (ii) identify candidate sex-linked genomic regions and putative sex-determining genes, as well as develop affordable and rapid male-specific DNA markers to determine the genetic sex of cobia; and (iii) elucidate the phylogenetic relationship between cobia and other teleosts via genome synteny.

## Results

### Assembly and annotation of chromosome-level male and female cobia genomes

**Figure 1:**
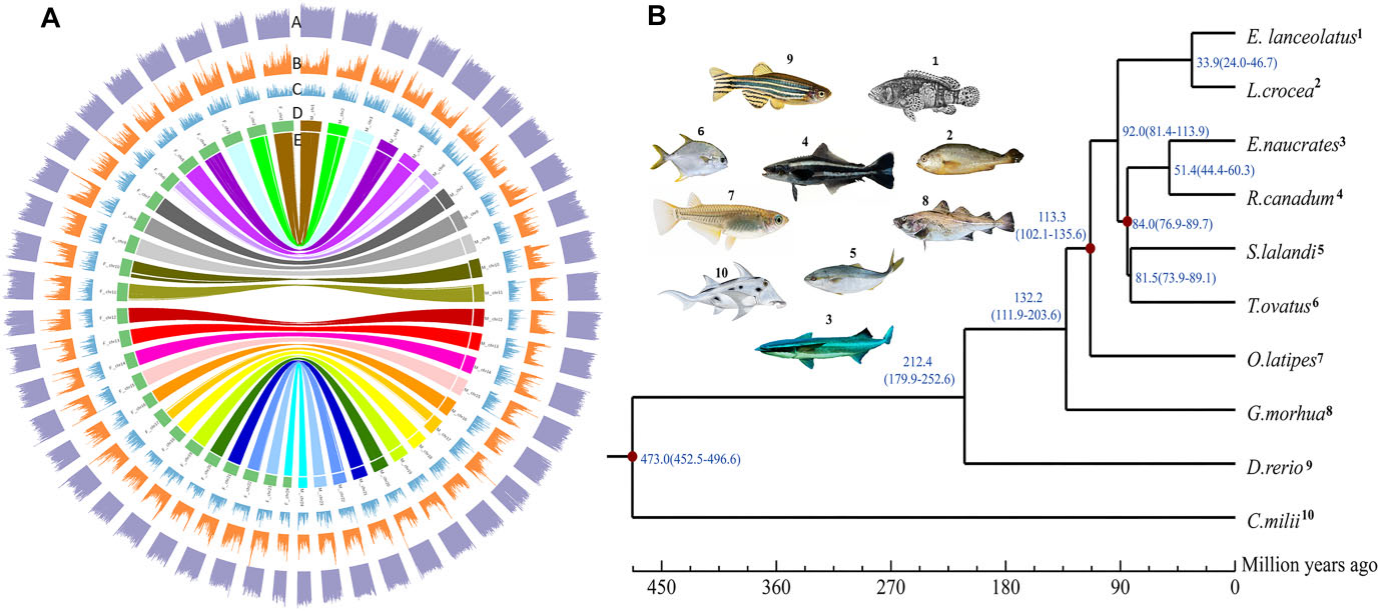
Overview of male and female cobia genome features. (A) Landscape of the 24 assembled cobia chromosomes. In the male genome, chromosome numbering is organized in descending order according to chromosome assembly size, whereas the female genome’s chromosome numbering follows a one-to-one syntenic relationship with the male genome. From the outer to the inner: (A) GC_content, (B) transposable element content density, (C) gene density, (D) chromosomes, and (E) syntenic relationship of female (left of the circle) and male (right of the circle) chromosomes. (B) Phylogenetic tree of 10 vertebrate genomes constructed using 572 single-copy orthologous genes. The numbers (blue) on the branches represent the estimated divergence time in million years ago (Mya). Time span in brackets were the 95% confidence interval of divergence time, and red circles indicate the calibration time from fossil. All nodes had support values of 100%.

We sequenced 1 male and 1 female cobia using single-tube long fragment read (stLFR) and Hi-C technologies, each sex with over 285-fold genome coverage (Supplementary Tables S1, S2). *De novo* genome assembly was performed on 87.07 Gb and 78.12 Gb of clean stLFR reads separately generated for male and female, respectively (Supplementary Table S1). A 586.23 Mb of the male genome was assembled with a contig/scaffold N50 of 86.0 kb/10.3 Mb (Supplementary Table S3), which is close to the 585.72 Mb estimate from *k*-mer analysis (Supplementary Fig. S1A). The genome assembly size for the female was 583.56 Mb (accounting for 99.2% of the *k*-mer estimated 588.46 Mb) with a contig/scaffold N50 of 79.9 kb/6.3 Mb (Supplementary Table S3 and Supplementary Fig. S1B). Detailed information on the estimation of the genome size based on *k*-mer analysis is shown in Supplementary File Note 1. To further improve the genome assembly and anchor the scaffold sequences to chromosomes, we generated 81.5 Gb and 103.1 Gb Hi-C data for the male and female, respectively (Supplementary Table S2). By incorporating the Hi-C data, 586.51 Mb of the male genome was assembled with a scaffold N50 of 24.3 Mb, whereas the female genome assembly size was 583.88 Mb with scaffold N50 of 22.5 Mb (Supplementary Table S3). A total of 563.06 Mb (96.00% of the assembly) of the male and 537.27 Mb (92.02% of the assembly) of the female genome sequence were ordered and oriented into 24 pseudo-chromosomes, respectively (Fig. 1A, Supplementary Fig. S2 and Table S4).

This outcome was consistent with the previous report on cobia karyotype (2n = 48) [29]. All 24 chromosomes of the male and female genomes showed a clear one-to-one syntenic relationship (Fig. 1A and Supplementary Table S4). The quality of the 2 genome assemblies was assessed in 2 aspects: (i) complete and single-copy BUSCO scores of 94.2% (male) and 93.8% (female) (Supplementary Table S5) and (B) an average of 96.45% and 97.88% RNA sequencing (RNA-seq) reads from gonadal tissues of cobia [8] could be mapped to the male and female genome assemblies, respectively. These results indicate that the assembled genomes were high quality.

A total of 21,604 and 21,688 protein-coding genes were separately annotated in the male and female genome assembly (Supplementary Table S3), and over 99% of them were annotated by a functional database (Supplementary Table S6). The BUSCO evaluation of the protein sequences identified 93.1% and 92.6% of complete single-copy genes for the male and female genome assembly, respectively (Supplementary Table S5). Approximately 11.08% of the male genome and 11.55% of the female genome were annotated as repetitive elements (Supplementary Tables S7, S8). We also identified 1,304 and 1,289 noncoding RNAs, with a total length of 116.9 kb and 117.0 kb in the male and female genomes, respectively (Supplementary Table S9).

### Phylogenetic construction and evolution analysis reveals *R. canadum* and *E. naucrates* as sister groups

To investigate the evolutionary relationship of cobia (*R. canadum*) and related teleosts, a phylogenetic tree was constructed using 572 single-copy orthologous genes of cobia and 9 other fish species (Supplementary Figs. S3, S4). Of these, 3 were Carangiformes, including 2 Carangidaes of *Trachinotus ovatus* (pompano) and *Seriola lalandi* (yellowtail amberjack), as well as one Echeneidae of *E. naucrates* (remora or live sharksucker). The remaining 6 were *Callorhinchus milii* (elephant shark), *Larimichthys crocea* (large yellow croker), *Danio rerio* (zebrafish), *O. latipes* (medaka), *Gadus morhua* (Atlantic cod), and *Epinephelus lanceolatus* (giant grouper). The phylogenetic relationship showed that *R. canadum* clustered within the order Carangiforme, together with *E. naucrates, S. lalandi*, and *T. ovatus*, which was consistent with results reported previously [31] and confirmed that *R. canadum* and *E. naucrates* were sister groups (Supplementary Fig. S5). From the estimates of divergence time, the ancestor of *R. canadum* separated from the ancestor of *E. naucrates* approximately 51.4 million years ago (Mya). The ancestor of *R. canadum* and *E. naucrates* separated from the ancestor of *S. lalandi* and *T. ovatus* approximately 84.0 Mya (Fig. 1B). In addition, the 24 pseudo-chromosomes of cobia had a clear one-to-one relationship to *E. naucrates* (Supplementary Fig. S6A), while 7 chromosomes (6, 7, 10, 13, 14, 15, and 17) of *R. canadum* were observed to have a hit to 2 or 3 chromosomes of *T. ovatus* (Supplementary Fig. S6B). Unfortunately, the chromosomal-level genome of *S. lalandi* was not available, so no syntenic relationship was explored between *R. canadum* and *S. lalandi*.

### Characterization of sex-specific regions in cobia

**Figure 2:**
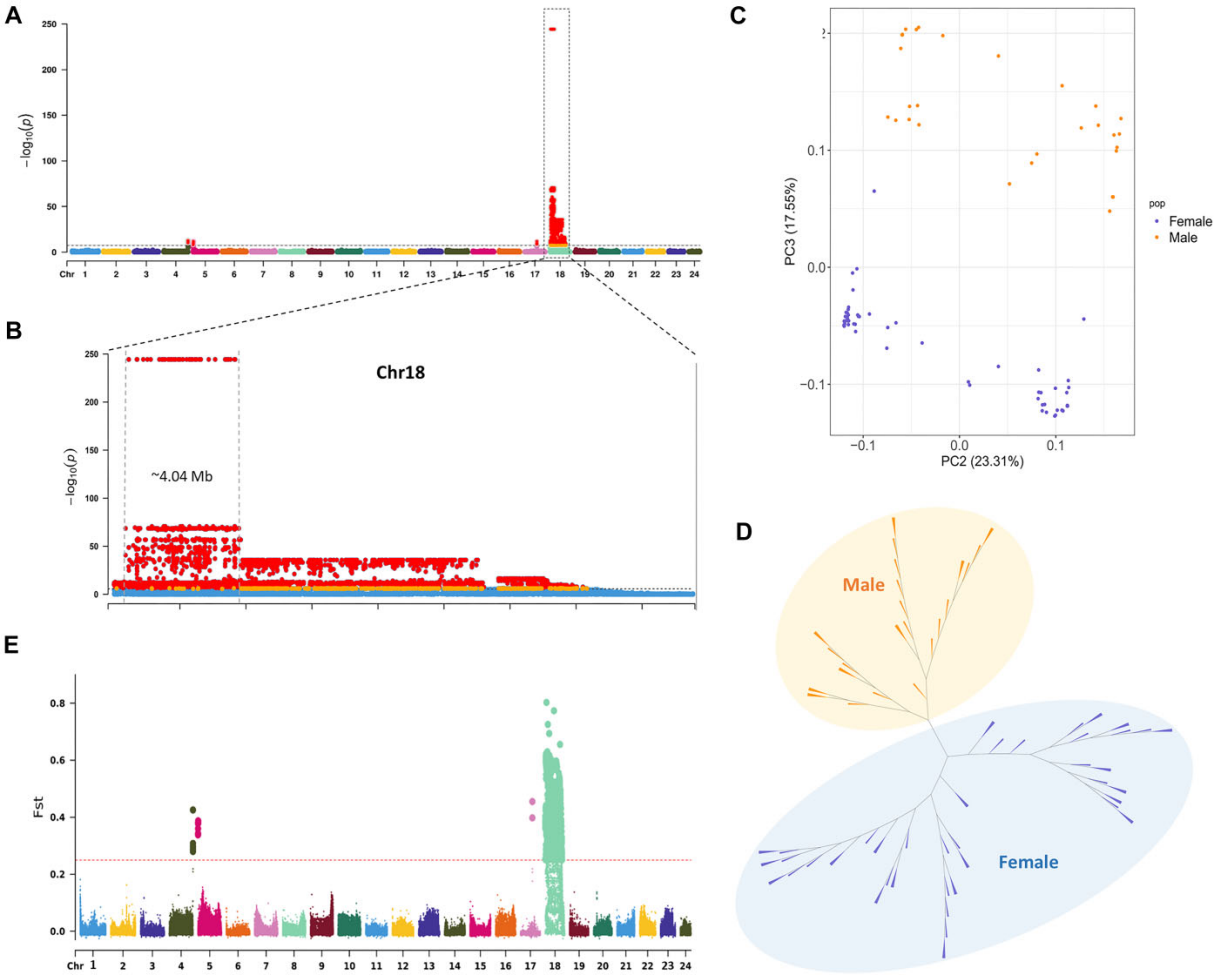
Genome-wide distribution of SNPs from 31 males and 60 females. (A) Manhattan plot showing –log_10_  *P* value of each SNP from the GWAS investigating sex-associated regions on the cobia genome. The horizontal line indicates the genome-wide significance threshold −log_10_(*P*)= 7.7. (B) The SNP distribution on Chr18. The 162 SNPs significantly associated with sex spanning over a region of ∼4.04 Mb (559.54 kb to 4.59 Mb). (C) Principal component analysis of 91 individuals using SNPs. (D) Phylogenetic tree showing relationships of females (blue) and males (orange). (E) Genome-wide scan of fixation index (*Fst*) matching the results from the GWAS.

To locate the sex-specific genomic region(s) of cobia, a total of 2,681 Gb of filtered whole-genome resequencing (WGRS) data were generated from 91 individuals (31 males and 60 females), with an average of ∼49-fold depth per sample (Supplementary Table S10). Using the male genome as reference, an average mapping rate of 99.0% per sample was obtained (Supplementary Table S10). In total, 551,838 filtered single-nucleotide polymorphisms (SNPs) were detected. The genome-wide association analysis (GWAS) using the male genome as reference revealed a single peak (−log *P* values of up to 244.37) with 162 SNPs significantly associated with sex, spanning over a region of ∼4.04 Mb (559.54 kb to 4.59 Mb) on male chromosome 18 (MChr18) (Fig. 2A, B and Supplementary Table S11). Most important, the 162 strongly sex-associated SNPs showed the same pattern where all 31 males were heterozygous, but homozygous for all 60 females (Supplementary Table S11). These results hinted at a putative male heterogametic or potential XX/XY model of sex determination, with a fully sex-linked region on MChr18. In addition, the principal component analysis (Fig. 2C) and a neighbor-joining tree (Fig. 2D) calculated using the SNPs from MChr18 showed that male and female individuals clustered into 2 distinct groups. Moreover, the relative component of genetic differentiation (estimated as *Fst*) between males and females further confirmed the region detected by GWAS (Fig. 2E). Hence, both the GWAS and *Fst* scan, which takes genetic structure into consideration, consistently identified a peak genomic region on MChr18, showing the highest probability as a sex-associated region in cobia. While GWAS indicated potential association signals in 3 other genomic areas (MChr4, MChr5, and MChr17) (Fig. 2A), a more detailed examination revealed that the SNP genotypes within these regions did not consistently exhibit heterozygosity in males and homozygosity in females (Supplementary Table S12). Furthermore, the resequencing data showed comparable coverage of these regions in both males and females. Additionally, 3 genes were identified on MChr4: *mdn1* (midasin AAA ATPase 1), *trm6* (tRNA methyltransferase 6 noncatalytic subunit), and *fermt1* (FERM domain containing kindlin 1), while no genes were detected on Chr5. On Chr17, *ostm1* (osteoclastogenesis associated transmembrane protein 1) was identified. However, these genes have not previously been reported to have a functional role in sexual development and function. Consequently, we conclude that it is improbable these regions play an important role in sex determination.

**Figure 3:**
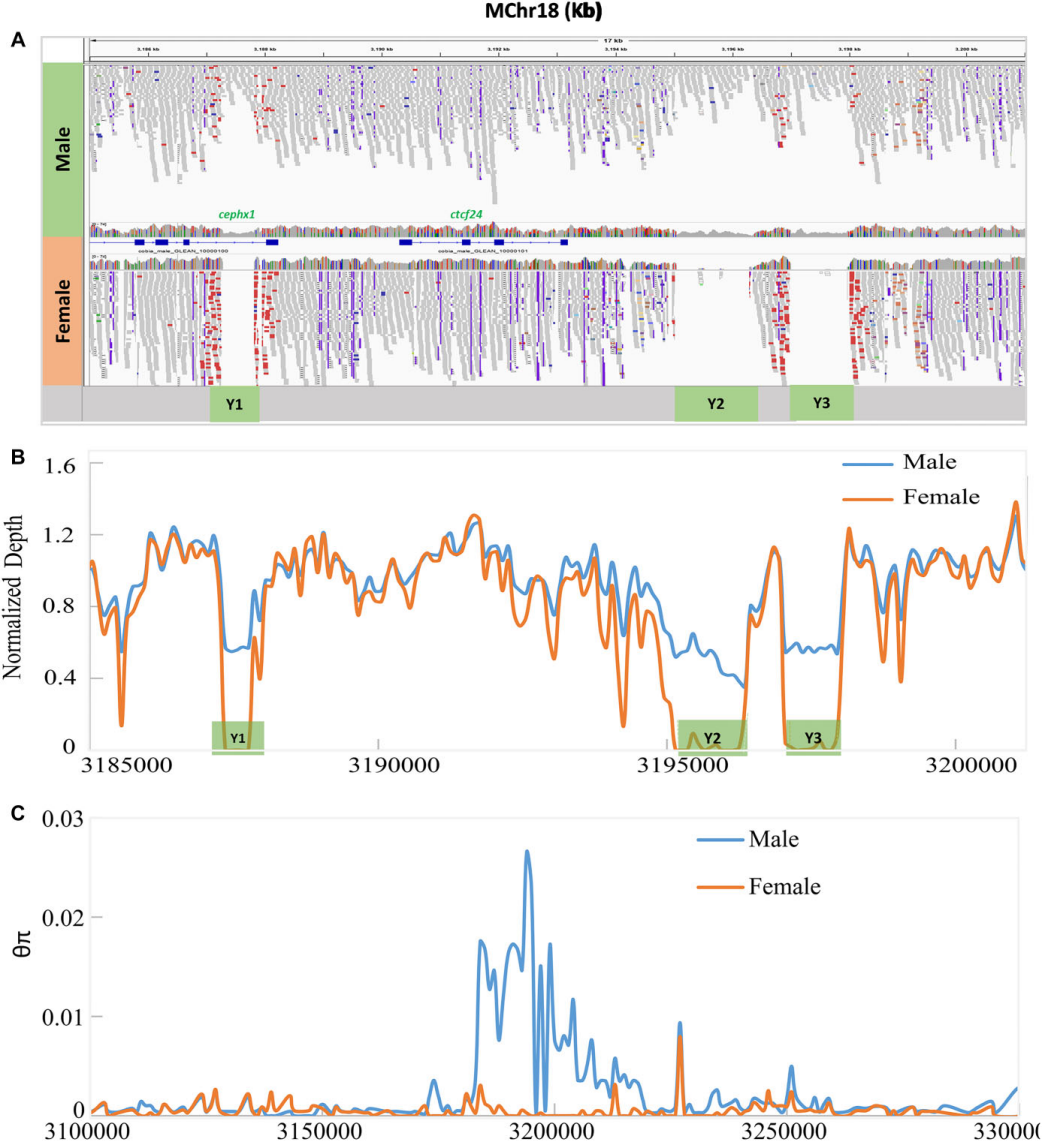
(A) Alignments of the stLFR reads from male and female individuals to the sex-associated regions. (B) Average depth normalized per group. For both A and B, the letters of Y1, Y2, and Y3 stand for the distinct regions between males and females. (C) Genetic diversity of sex-determining region. Blue line indicates the female group, and yellow line represents the male group.

The genome-wide difference of sequencing depth between males and females was also analyzed to identify the sex-specific region(s) in cobia. By investigating the mean depth (sites depth/average depth), the sex-linked region identified above was further narrowed down on MChr18. Unfortunately, an unacceptable number of gaps with variable length were observed within the region and the flanking regions. To improve the contiguity of this sex-associated region, we performed PacBio HiFi sequencing of the DNA from a male cobia individual, which rendered 1,170,581 highly accurate PacBio long reads with an average length of 16.1 kb (longest read: 40.4 kb; N50 = 15.9 kb) totaling 18.86 Gb, representing 32× coverage of the male genome. This set of PacBio reads was assembled first and then aligned to the MChr18. A large scaffold of 15.99 Mb from the PacBio genome assembly that contained the above identified sex-associated region was further reassembled with MChr18. The result was a new MCh18 with a total length of 21.98 Mb, and 843 genes (68 more than in the original MCh18) were detected from its reannotation (Supplementary Table S13). Most important, all the gaps presenting within the sex-linked region and its flanking regions in the original MChr18 were fully filled. GWAS analysis was carried out on the newly assembled MChr18, which detected 232 SNPs significantly associated with sex in a single peak. Consistently, all 232 SNPs showed that all 31 males were heterozygous, but all 60 females were homozygous (Supplementary Table S14). Interestingly, further sequencing depth analysis revealed that 3 short male-specific regions of Y1 (400 bp; 3,187,350 to 3,187,750 bp), Y2 (1,100 bp; 3,195,150 to 3,196,250 bp), and Y3 (1,000 bp; 3,197,050 to 3,198,050 bp) within a continuous region of 10.7 kb were discovered within the sex region, which showed no WGRS reads mapped from 60 females (corresponding depth of zero for females) but with a mean depth of 0.5 (haploid copy specific to males) in 31 males (Fig. 3A, B), suggesting that the Y1, Y2, and Y3 are putative male-determining regions that could contain the candidate master sex-determining genes. In addition, the θπ value analysis showed that the divergence mainly came from the male group (Fig. 3C).

### The *cephx1y* and *ctcf24y:* the putative drivers of cobia sex determination

**Figure 4:**
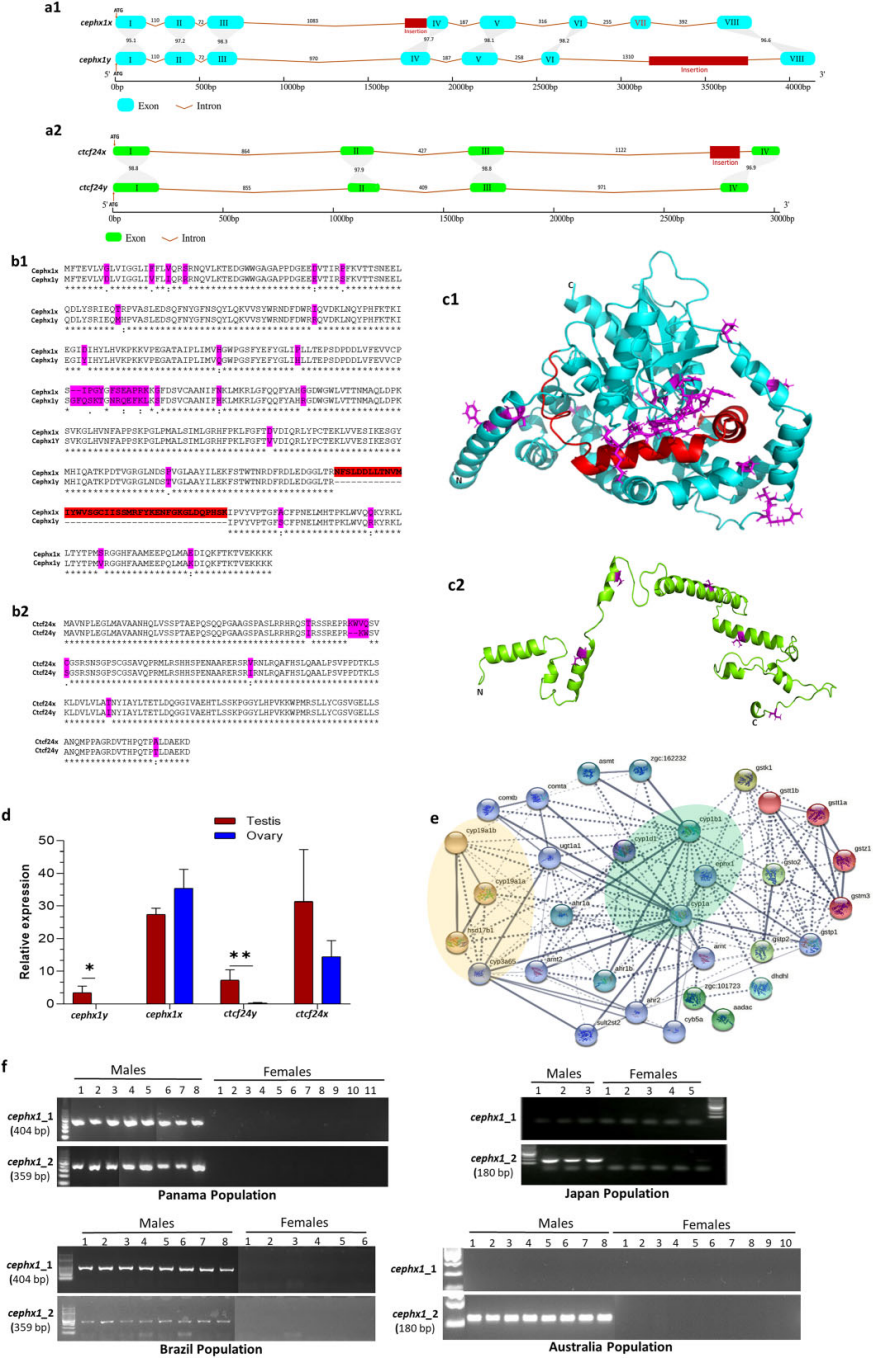
(A) Schematic representation gene structure of *cephx1y* and *cephx1x, ctcf24y* and *ctcf24x* in cobia. The red boxes indicate sex-specific insertions. (B) Amino acid sequence alignment of *cephx1y* and *cephx1x*, as well as *ctcf24y* and *ctcf24x* of cobia. (C) Structural model of cobia *ephx1* (c1_Blue color) and *tcf24* (c2_Green color). The red color represents an extra helix-turn-helix domain in *cephx1*; the purple color indicates amino acid differences between the paralogs. C, C-terminus; N, N-terminus. (D) The expression profile of *ephx1* and *tcf24* in the testes and ovaries based on gonadal transcriptomic analysis. “*” and “**” indicate *P* < 0.01 and *P* < 0.001, respectively. (E) The interaction of *cephx1y* with CYP components (green and yellow shaded) and other proteins based on the STRING PPI network. Nodes represent genes, and edges represent correlation between nodes. (F) Sex-specific markers in cobia. The PCR amplification in Panama, Brazil, Japan, and Australia populations.

We further scanned the 10.7-kb sex-associated region on MChr18. A short insertion (540 bp within the sixth intron region) in a functionally annotated gene of epoxide hydrolase 1_*ephx1* (3,184,084 to 3,188,235 bp) was identified in the male-specific region Y1. There were no genes detected in regions Y2 and Y3 (Fig. 3A). However, another gene, transcription factor 24_*tcf24* (3,190,353 to 3,193,193 bp), was detected between Y1 and Y2 (Fig. 3A). These 2 genes, especially the *ephx1*, were considered of high interest for male function in cobia. In addition, both genes were also found in the homologous female chromosome of FChr18 (19.29 Mb). Alignment of *ephx1* and *tcf24* genomic sequences in MChr18 and FChr18 revealed a high nucleotide identity of 96.6% and 95.9%, respectively. Indel (insertion–deletion) variants with variable length and SNPs also existed in both gene (coding regions and introns) comparison groups (Supplementary File: Genomic DNA sequence alignment of *ephx1* and *tcf24*). The 2 genes on MChr18 were termed cobia *ephx1y* (*cephx1y*) and cobia *tcf24y* (*ctcf24y*), as well as *cephx1x* and *ctcf24x* for Fchr18. As nucleotide sequence divergence impacts protein sequence, gene structure predictions were performed for both genes. The results showed that *cephx1x* spans about 3.78 kb and consists of 7 introns and 8 exons (Fig. 4A1) encoding 455 amino acids (Fig. 4B1). However, only 6 introns and 7 exons were detected for *cephx1y* with a total length of 4.15 kb, which showed 1 exon (VII) absent (Fig. 4A1) encoding 416 amino acid residues (Fig. 4B1).

The nucleotide identity between exon sequences of *cephx1x* and *cephx1y* ranged from 95.1% to 98.3% with an average of 97.3% (Fig. 4A1). A closer look at *cephx1x* and *cephx1y* revealed that the largest sequence differences were 2 indels of 165 bp of X-specific insertion and 540 bp of Y-specific insertion in the noncoding regions (Fig. 4A1), while the remaining were randomly distributed SNPs and short indels (Supplementary File). It is noteworthy that the Y1 region was in fact the male-specific fragment specifically inserted in the sixth intron of the *cephx1y* (Fig. 4A1 and Supplementary File). In terms of *tcf24*, the *ctcf24y* contained the same number of exons (4) and introns (3) as *ctcf24x* (Fig. 4A2), *ctcf24y* spans 2,840 bp and encodes 202 amino acids, and *ctcf24x* has 3,024 bp with a translated protein product of 204 amino acids (Fig. 4B2). The observed sequence identity in exons was 96.9% to 98.8%. There was also a large 220-bp X-specific insertion in the third intron of *ctcf24x*, and several small indels and SNPs were also detected between them (Fig. 4B1 and Supplementary File). Moreover, we built a structural model for both genes. The Ephx1 is a protein coding gene, with the Cephx1y protein folds essentially identical to Cephx1x (Cα root mean square deviation of 1.108 Å), while the Cephx1x had an extra 41–amino acid helix-turn-helix domain (missing in Cephx1y), which plays an important role in the stability of the protein (Fig. 4C1). In addition, the amino acid alignment of the 2 *ephx1* genes of cobia and other fish like *E. naucrates, E. lanceolatus*, and *Seriola dumerili* revealed that the loss of the helix-turn-helix domain existed only in the Cephx1y (Supplementary File). Both cobia *tcf24* counterparts lack a fixed or ordered 3-dimensional structure, and a total of 9 amino acid differences at 6 sites were detected between Ctcf24x and Ctcf24y (Fig. 4C2).

A further investigation of the expression pattern of *ephx1* and *tcf24* by examining the cobia gonadal transcriptome [8] showed that both *cephx1y* and *ctcf24y* were significantly differentially expressed between males and females (Fig. 4D). The *ctcf24y* was more highly expressed in testis (Fragments Per Kilobase per Million mapped fragments [FPKM]: 7.27) than ovaries (FPKM: 0.28), with the log_2_ (fold change [FC]) of ovary/testis of −4.8. In addition, the expression of *cephx1y* was observed in all 5 testis samples (1.5 ≤ FPKMs ≤ 6.3) but only in 1 of 5 ovary samples (0 ≤ FPKMs ≤ 0.02), with the log_2_FC = −9.5, indicating this gene was nearly exclusively expressed in male cobia (Fig. 4D). The *cephx1x* and *ctcf24x* showed no significant differential expression between testes and ovaries. In addition, protein–protein interaction (PPI) network analysis showed that the *cephx1y* interacts directly and significantly with 3 cyp1 genes: *cyp1a1, cyp1b1*, and *cyp1d1* (Fig. 4E). Testis-specific expression was observed for *cyp1a1*, while *cyp1b1* was overexpressed in the ovary, and *cyp1d1* was absent [8]. The *ephx1* gene together with *cyp1a1* and *cyp1b1* are involved in the metabolism of xenobiotics through the cytochrome P450 pathway (Supplementary Fig. S7). In addition, the *cephx1y* also interacts indirectly with *hsd17b1* and 3 other CYP components: *cyp19a1a* (gonadal aromatase), *cyp19a1b* (brain aromatase), and *cyp3a65* (predicted to enable steroid hydroxylase activity in zebrafish).

### Sex-specific DNA markers of development and population specificity validation

To develop sex-specific markers, 2 forward (F) and 2 reverse (R) primers were designed to target the male-specific fragment insertion region within the sixth intron of *cephx1y*. The first set of primers, *cephx1_*1 (F1–R1), was designed to amplify a 404-bp region where there was an absolute deletion of the fragment for *cephx1x* in the region of FChr18; therefore, the PCR would only amplify the “Y” sequence. The second set of primers, *cephx1_*2 (F2–R2), had the forward primer located in the male-specific region of *cephx1y*, while the reverse primer (R2) was situated in a region common to both *cephx1y* and *cephx1x*, resulting in a predicted PCR product size of 359 bp. Following the PCR validation of 8 male and 11 female cobia individuals from Panama, both pairs of primers successfully amplified a male-specific product in all the unambiguously phenotypically sexed males (Fig. 4F).

Furthermore, distinct patterns were observed for the 2 markers during the analysis of cross-population validation. Similarly to Panama population, both *cephx1*_1 and *cephx1*_2 successfully amplified a PCR product from all males and was absent from females within the population of Brazil. Sanger sequencing of the PCR products, using both sets primers, confirmed the accuracy of the target sequences in both Panama and Brazilian populations. However, *cephx1_*1 did not show any amplification in both males and females in Japan and Australia populations, while *cephx1*_2 successfully amplified a shorter 180-bp product exclusively in males, and females showed no amplification. In contrast, a longer 359-bp product was amplified from male DNA originating from the Brazilian and Panama populations (Fig. 4F). Subsequent Sanger sequencing of PCR products confirmed the presence of the 180-bp fragment in Japan and Australia populations. In addition, the failure of *cephx1_1* to amplify in Japan and Australia populations is due to its reverse primer being situated within the 179-bp (359 bp − 180 bp) missing region.

## Discussion

The absence of a chromosome-level reference genome for cobia posed a significant challenge for in-depth genomic analysis for the species, such as the investigation on its phylogenetic relationship with other teleosts and sex determination mechanisms. In the present study, we successfully obtained 2 high-quality genomes for both sexes of cobia using a combined strategy involving stLFR and Hi-C technologies. The high BUSCO score of 94.2% for the male and 93.8% for the female indicates the completeness of the 2 genome assemblies. To our knowledge, this represents the first annotated chromosome-level reference genome of the species. These resources will provide researchers with opportunities to explore the molecular mechanisms controlling cobia’s sex determination system and other economically important traits through genomic selection for faster growth, disease resistance, and high-quality fillets. Moreover, it may also allow the development of further in-depth studies to better understand the biology of the species, such as how this large pelagic migrant inhabits all tropical and subtropical oceans of the globe and to inform more sustainable fisheries management practices.

The study of sex determination systems in teleosts can be technically challenging as most of them possess undifferentiated sex chromosomes and have various complex and diverse mechanisms for determining sex [34, 35]. GWAS have gained popularity in exploring sex determination mechanisms, enabling the identification of sex-linked markers, sex determination loci, and candidate genes [36–39]. In this study, we conducted GWAS to investigate the genetic basis of sex determination in cobia and identified a sex-associated region on MChr18, with 232 most significantly sex-linked SNPs that presented as heterozygote genotypes in all males and complete homozygosity in all females of the captive Panama cobia population. The high density of sex-specific SNPs was a feature of the putative sex determination locus, which has also been observed in sex determination studies of other aquatic species [36, 40]. Moreover, this male-specific heterozygosity pattern suggested that the cobia may possess a putative male heterogametic sex determination system (XX/XY), which would be consistent with being a gonochoristic species [1]. In addition, the selection signatures of the fixation index *Fst* and sequencing depth analysis served to further strengthen the evidence for the potential sex determination locus identified through GWAS. Taken together, the integration of GWAS, *Fst* scanning, and sequencing coverage analysis identified a strongly sex-linked region and provided the evidence that the MChr18 is the potential undifferentiated homologue containing sex-specific loci, which is in agreement with the previous karyotype analysis where no morphologically distinct sex chromosomes for cobia were found [29]. The combination of these strategies has proven to be an effective approach for investigating the putative sex determination mechanism in cobia, as well as in other species [36, 41, 42]. Furthermore, it is noteworthy that the sex-associated region on MChr18, identified preliminarily in the male genome assembly by combining the stLFR and Hi-C reads, contained several gaps with variable length within the region and the flanking regions. The highly repeated content of this region complicated the assembly of the region. By further adding the PacBio HiFi reads, we obtained a small gap-free sex-linked region of 10.7 kb. This highlights the power of using long sequencing reads to assemble highly repetitive and complex genomic regions.

The small sex-associated region characterized on MChr18 contains 2 putative master sex-determining genes for cobia, *ephx1* (a short male-specific fragment insertion within Y1) and *tcf24* (between Y1 and Y2). Although *ephx1* has not previously been reported as a master sex gene or linked to sex determination, it is known to regulate endogenous steroid metabolism (i.e., androgens and estrogens), suggesting a functional role in sexual development and function in mammals [43]. A previous study in humans showed that upon treatment with an *ephx1* inhibitor, a decrease in estradiol formation was seen in ovaries [44]. In mice, *ephx1* is upregulated in the embryo-containing oviduct and is thought to play a role in preimplantation embryo development [45]. However, its reproductive function in fish remains poorly studied [46–48]. Here, the *cepxh1y* was observed to be nearly exclusively expressed in the testes of adult fish by examining a recently published gonadal transcriptome of cobia [8]. In addition, a small male-specific insertion was detected in the sixth intron of *cephx1y*, which was in fact the identified male-specific region of Y1. Moreover, the loss of exon VII (a 41–amino acid helix-turn-helix domain) was only observed in the *cephx1Y* when compared to that of *cephx1x* and the other fish species and mammals. All these findings suggest that *cephx1y* could be a potential sex-determining gene in cobia. In addition, the PPI network analysis showed that *cephx1y* exhibits direct or indirect interactions with 6 Cyp genes and *hsd17b1*. The Cyp genes, specifically P450 aromatase (*cyp19a1a*), are known to have a crucial function in the development of gonads in various fish species [49, 50]. The *hsd17b1*, a gene involved in the steroidogenic pathway, has been recognized as a master sex-determining gene in yellowtail species [26], which belong to the same order (Carangiformes) as cobia.

The *ephx1* gene encodes microsomal epoxide hydrolase (EPHX1), an enzyme known to be involved in the metabolism of xenobiotics and is thought to mediate functions including bioactivation and detoxification of environmental deleterious compounds [45, 51]. The occurrence of cobia intersex individuals has been reported in India [28] and Australia [3]. The observation of 17% intersex individuals is attributed to increased levels of endocrine-disrupting compounds (EDCs) from industrial and agricultural pollutants in local waterways. Interestingly, the EPHX1 enzyme has been reported to be involved in xenobiotic metabolism and regulates endogenous steroid metabolism [43]. Therefore, it stands to reason that EDC exposure could have an effect on EPHX1 catalytic activity, disrupting its functional associations with the cytochrome P450 family, which mediate sex determination and differentiation pathways and potentially cause aberrations in gonadal development of cobia, more so given that *cephx1y* is the only male-specific gene detected in the species. Fish exhibit a wide variety of sex-determining genes (for review, see [17]), and more “newcomers” with no previously known role in sex determination have also been discovered in recent years, such as *Paics* in blue tilapia (*Oreochromis aureus*) [52] and *bcar1* in channel catfish (*Ictalurus punctatus*) [27]. The present study has identified *cephx1y* as a novel potential sex determination gene in cobia, offering new knowledge on the molecular mechanisms involved in teleost sex determination. Further functional experiments, such as genome editing, are necessary to confirm and further explore these findings, as well as to clarify the complete mechanism by which *cephx1y* might modulate aromatase activity or other steps of the steroidogenic pathway in cobia and potentially other teleosts.

The transcription factor *tcf24* was first described in humans in 2002, but its functions remain largely unknown [53]. The only publication related to this factor in fish revealed that *tcf24* is upregulated in the hindbrain of individually housed three-spined stickleback (*Gasterosteus aculeatus*) as a molecular basis for social behavior [54]. The *ctcf24y* was significantly upregulated in the testes by examining the data from the cobia gonadal transcriptome [8], suggesting a potential role in testicular differentiation of cobia. It is important to note that *tcf24* has a paralog, *tcf23* (also called OUT), which plays a role in mammalian reproduction. In humans, *tcf23* is a newly identified decidual mediator of progesterone action [55]. In mice, it was expressed in adult reproductive tissues (e.g., uterus, ovaries, and testes) [56], indicating its potential role in male and female reproductive biology. Studies in fish have also shown that *tcf23* was highly upregulated in the ovaries of coho salmon (*Oncorhynchus kisutch*) after treatment with 11-KT (11-ketotestosterone). In rainbow trout, *tcf23* was detected exclusively in the gonads of both sexes [57]. To date, functional studies of both *tcf24* and *tcf23* on reproductive biology are still very limited, particularly in teleost fishes. Thus, further detailed functional characterization of *tcf24* is required to understand its potential role in sex determination and differentiation in cobia.

Identifying reliable and universally applicable sex-linked markers in fish poses challenges due to the considerable variability in sex determination genes and systems, even among closely related species and within populations of the same species [58–60]. The current research successfully developed and validated 2 male-specific PCR-based markers (overlapping amplicons) targeting *cephx1* for the cobia population of Panama. These 2 markers were validated and shown to be amplifiable only in males among individuals from Brazil. In the Japan and Australia populations, primers for *cephx1_1* did not amplify, while primers for *cephx1_2* amplified shorter 180-bp products in males compared to the 359 bp found in the Brazil and Panama populations, revealing a shorter *cephx1y* intron 6 in the Asian and Australian populations when compared with the 2 populations from the Americas. Nevertheless, the absence of *cephx1y* in cobia females in far-distant populations across the globe indicates a conserved role of *cephx1y* as a key putative sex-determining gene for the species. The development of this simple sex-specific PCR tool has the potential to significantly improve artificial fertilization and precise breeding in the cobia aquaculture industry, ultimately leading to the development of monosex populations and increased productivity. Additionally, it aids nonlethal sampling and improves animal welfare in breeding programs.

The placement of the cobia (*R. canadum*) within the Carangiformes order and its status as the only member of the Rachycentridae family are well established. However, there have been divergent findings regarding its phylogenetic relationship to other species, particularly whether it is more closely related to the Coryphaenidae or Echeneidae [31–33, 61, 62]. In the current study, comparative genome analysis between *R. canadum* and 1 Echeneidae (*E. naucrates*), as well as 2 Carangidaes (*T. ovatus* and *S. lalandi*), showed that *R. canadum* and *E. naucrates* were sister groups, and the ancestor of *R. canadum* separated from the ancestor of *E. naucrates* approximately 51.4 million years ago. This investigation represents the first of its kind and provides insights into the evolutionary relationship of *R. canadum* through comparative genomic and phylogenetic analysis. Unfortunately, genomes of the only 2 species within the Coryphaenidae family, the mahi-mahi or common dolphinfish (*C. hippurus*) and the pompano dolphinfish (*Coryphaena equiselis*), are not yet available. Therefore, a more complete study of the evolution of the Rachycentridae genome (cobia as a single representative) needs to be further investigated when dolphinfish genomes become available.

## Conclusions

We have successfully assembled and annotated high-quality chromosome-level reference genomes for male and female cobia, which will provide a valuable resource for future investigations into the population structure, evolutionary history, fisheries management, and conservation of cobia and other Carangiformes species. Furthermore, the findings of this study suggest that cobia may harbor a putative male heterogametic (XX/XY) genetic sex determination system, with 2 genes, *cephx1y* and *ctcf24y*, as potential putative main drivers of cobia sex determination. Notably, *cephx1y* could represent a putative novel sex-determining gene, which further supports the rapid evolution of sex-determining mechanisms in teleost fish. Moreover, our development of a practical PCR-based method for identifying genetic sex in cobia can assist in breeding monosex female populations in commercial farming of the species.

## Materials and Methods

### Experiential fish and sample collection

The majority of cobia individuals used in this study were obtained from Open Blue Sea Farms, the Republic of Panama. One male and 1 female adult fish at 2 years old were sampled for the whole-genome *de novo* sequencing and assembly. In addition, a total of 91 fin clips from adult fish (31 males and 60 females) were sampled for whole-genome resequencing. For the development and validation of sex-specific DNA markers, the ovary and testis tissues were dissected from 5 male and 5 female fish, and 9 fin clips (from the 91 referenced above) were chosen from 3 male and 6 female fish. Moreover, to validate the population specificity of the sex-specific DNA markers, fin clips from adult fish were obtained from 3 additional cultured populations in Japan (3 males and 5 females), Brazil (8 males and 5 females), and Australia (8 males and 10 females). Sex of fish individuals was determined through cannulation or gonadal observations.

### Genome sequencing

High-quality and molecular weight genomic DNA was extracted from fin clips of male and female cobia with a QIAamp DNA purification kit (Qiagen) in accordance with the manufacturer’s protocol. Paired-end stLFR libraries [63] and Hi-C libraries were constructed using published protocols available via protocols.io [64] and sequenced on the BGISEQ-500 platform (BGI; RRID:SCR_017979) [65], yielding 100-bp paired-end (PE) reads. Barcodes were first split from stLFR raw reads and subsequently filtered by Soapfilter v2.2 (parameter: -y –p –M 2 –f -1 –Q 10) to generate high-quality sequences. The genome sizes of the male and female cobia were estimated based on *k*-mer analysis (*k* = 17) using Jellyfish v2.2.6 [66] and Genome Scope v1.0 [67]. Genome size was estimated with the formula genome size (Mb) = *k*-mer number/*k*-mer depth. For PacBio sequencing, high molecular weight genomic DNA from testis was extracted using a standard phenol/chloroform method. The testis was selected because it ensures certainty regarding the sex and it yielded high-quality DNA. The integrity of the extracted DNA was assessed by 0.75% agarose gel electrophoresis, and the concentration was quantified by a Qubit 4 Fluorometer (Thermo Fisher Scientific). Ten micrograms of DNA was then used to construct the library for PacBio SMRT sequencing using the SMRTbell express template prep kit (PacBio). The library was sequenced using the PacBio Sequel II System (RRID:SCR_017990) with HiFi sequencing modes.

### Genome assembly

The high-quality paired-end stLFRs, with read length of 100 bp, were used for initial genome assembly by employing the 10X Genomics software supernova [67]. First, the format of high-quality reads was transformed to 10X Genomics format, and then the male and female cobia genomes were separately assembled with Supernova v2.1.1. To further improve the quality of the assembly, Gapcloser (v1.12; RRID:SCR_015026) [68] was used with default parameters to fill gaps. Furthermore, Purge_haplotigs (RRID:SCR_017616) [69] was used to reduce redundancy of the initial assembly. The uniformity and completeness of the cobia male and female genome assemblies were evaluated by the read mapping rate as well as BUSCO [70]. Finally, chromosome-level assemblies were constructed using Hi-C data. HiC-Pro v3.2 (RRID:SCR_017643) [71] was utilized to perform quality control of raw reads. Valid reads (the reads with contact information after processing of HiC-Pro pipeline, including read alignment, detection and filtering of valid interaction products, binning, and contact map normalization) were used for assignment of contigs or scaffolds to chromosomes. Juicer v1.5 (RRID:SCR_017226) [72] and 3D-DNA (3D *de novo* assembly) [73] was used to anchor the male and female cobia genome assembly onto pseudo-chromosomes. In order to enhance the continuity of the sex-associated region, we further conducted genome assembly of a male cobia using PacBio reads. The obtained HiFi long reads were fed to hifiasm (v0.14.1-r314; RRID:SCR_021069) with the default parameters, and the primary assembly result p ctg.gfa file was converted into FASTA format with in-house scripts.

### Genome annotation

RepeatModeler v1.0.8 (RRID:SCR_015027) [74], LTR_FINDER v1.0.6 (RRID:SCR_015247) [75], and TRF tool v.4.09 (RRID:SCR_022193) [76] were used for *de novo* prediction of repeat elements based on the features of the repeat sequences. Homolog-based searches against the RepBase database (v21.01) [77] using RepeatMasker v.3.3.0 (RRID:SCR_012954) and RepeatProteinMask v.3.3.0 were performed. Protein-coding genes were identified using a combination of homology-based and *de novo* prediction. For the homology-based gene prediction, homologous protein sequences of 6 well-annotated fish species, including zebrafish, tongue sole, stickleback, tilapia, medaka, and Japanese pufferfish, were downloaded from Ensembl (release 94), while large yellow croaker was from NCBI. First, homologous proteins were aligned with the cobia genome using BLAT v319 (RRID:SCR_011919) [78], and then GeneWise v2.4.1 (RRID:SCR_015054) [79] was employed to predict genes. For the *de novo* prediction, the *ab initio* gene prediction program of Augustus software v3.1 (RRID:SCR_008417) [80] was chosen, adopting zebrafish genes as a training dataset. Gene sets were integrated into a comprehensive and nonredundant gene set using GLEAN [81]. The completeness of the final gene set was assessed by searching for 4,584 single-copy actinopterygian genes in BUSCO. Noncoding RNAs (microRNA and ribosomal RNA) were also identified by aligning the cobia genome sequences to Rfam [82] using Infernal v1.1.1 (RRID:SCR_011809) [83], and transfer RNAs (tRNAs) were defined using tRNAscan-SE v1.3.1 software (RRID:SCR_008637) with eukaryote default parameters. Functional annotation of the predicted protein-coding genes was conducted by aligning the predicted protein sequences to the public database, including SwissProt, Interpro, TrEMBL, and KEGG databases, using BLASTp with a maximal e-value of 1e-05.

### Phylogenetic tree construction and divergence time estimation

To confirm the evolutionary status of cobia, 9 other fish species, including *C. milii, E. naucrates, S. lalandi, T. ovatus, E. lanceolatus, L. crocea, D. rerio, O. latipes*, and *G. morhua*, were selected to uncover orthologous gene sets and conduct genome phylogenetic analysis. The male cobia genome was chosen as the representative of *R. canadum* to define gene families. For the other 9 teleosts, protein sequences of *C. milii, E. naucrates, S. lalandi, L. crocea, D. rerio, O. latipes*, and *G. morhua* were downloaded from Ensembl (release 99); the *E. lanceolatu* from NCBI (GCF_005,281,545.1); and *T. ovatus* from Figshare [84, 85]. All-to-all orthologous genes were aligned using BLASTP v2.2.26 with an e-value cutoff of 1e-7. Gene families were clustered by TreeFam [86] pipeline. For phylogenetic tree analysis, single-copy gene families from male cobia and 9 other fish species were aligned using MUSCLE v3.8.31 (RRID:SCR_011812) [87]. Phase 1 sites were extracted and merged to a supergene as an input of MrBayes v3.1.2 (RRID:SCR_012067) [88] with *C. milii* as the outgroup. The divergence time for cobia and the other 3 Carangiformes (*T. ovatus*, S. *lalandi*, and *E. naucrates*) was estimated by MCMCTree from the PAML v4.4 (RRID:SCR_014932) [89] package based on the HKY85 model. Correlated rates were used for a molecular clock model. Three-calibration fossil evidence was found using the website TimeTree [90], including *C. milii* with other teleost fish (453–497 Mya), *O. latipes* with perciformes species (104–145 Mya), and *E. naucrates* with *S. lalandi* (70–86 Mya).

### Whole-genome resequencing and identification of the sex-specific genomic region

Genomic DNA was isolated from the fin clips of individual fish (31 males and 60 females) and used to construct 100-bp PE libraries and sequenced with the Dipseq-T1 platform. Raw reads that contained more than 10% Ns, contained adaptors, or had a half base quality below 12 were discarded. Filtered reads (2,680 Gbp in total) were then mapped to the male reference genome, which resulted in an average mapping rate of 99.04% and 49.42× depth. The population SNPs were called with Accelerated Sentieon node [91], and sites were filtered that matched the condition “QD < 2.0 || MQ < 40.0 || MQRankSum < -12.5 || ReadPosRankSum < -8.0 || FS>60.0 || SOR>3.0.” Finally, a merged vcf for 91 samples with filtered SNPs (filtering with –max-missing 0.8 –maf 0.05 –minDP 4 –min-meanDP 3) on chromosomes were generated and used for later comparative analysis. Filtered SNPs were annotated by SnpEff (v 4.3t; RRID:SCR_005191) [92] and then classified into regions of exon, intron, splicing site, and upstream and downstream intergenic regions.

Using the male genome as reference, we employed 2 different strategies to identify the sex-specific region(s) in cobia. A GWAS was first performed using EMMAX [93], a mixed linear model, to test whether any of the SNPs identified were significantly associated with sex. The first 10 PCs of PCA from plink (v1.90b6.12) were used as concomitant variables at the same time. Second, we calculated depth of each site for all 91 samples using samtools-depth module (v-1.9) [94]. The average depth distribution analysis between the male and female group (bin 50 bp, normalized per sequencing depth of each sample) was also employed by exploiting the difference in sex chromosome ploidy between males and females. The *Fst* (Wright’s fixation index) between male and female groups and θπ (nucleotide diversity) of each group were calculated by vcftools (v0.1.13) [95]. A variant density approach was performed by searching for differences in SNP density between males and females. The PPI network prediction [96, 97] was adopted for the identified putative master sex-determining gene for cobia.

### Development of sex-specific markers and population specificity validation

Sex-specific primers were designed using Primer3 (RRID:SCR_003139) [98] in Geneious Prime 2021.2.2 (Biomatters). Two sets of primers (*cephx1_1*) (forward: 5′-ATCCAACATTTCAAGATCAACAGGTT-3′; reverse: 5′-GGGGACATCCTGATATCTAACCAATA-3′) (*cephx1_2*) (forward: 5′-GCTAGTTTAGAAAATGACAGCTCACA-3′; Reverse: 5′-GTAAAATTCCAAGATGTGAACAAGCC-3′) for *cephx1* were designed based on a 540-bp continuous fragment insertion in males where there is an absolute deletion for the gene in females. PCR conditions were first tested on 2 individual samples (1 male and 1 female) to verify PCR amplification and presence (in males)/absence (in females) polymorphism, then further screened on more sexed fish from Panama, Brazil, Japan, and Australia [99].

## Supplementary Material

giae034_GIGA-D-23-00328_Original_Submission

giae034_GIGA-D-23-00328_Revision_1

giae034_Response_to_Reviewer_Comments_Original_Submission

giae034_Reviewer_1_Report_Original_Submission Chris Armit -- 2/7/2024 Reviewed

giae034_Reviewer_1_Report_Revision_1 Chris Armit -- 4/17/2024 Reviewed

giae034_Reviewer_2_Report_Original_Submission Shangpeng Sun -- 2/26/2024 Reviewed

giae034_Reviewer_3_Report_Original_Submission Michael Pound -- 3/30/2024 Reviewed

giae034_Supplemental_Files

## Data Availability

The raw read data used to generate the genome assembly and the whole - resequencing data have been deposited in the NCBI BioProject database under accession code PRJNA864890. The final assembly data of the male and female cobia genome have been submitted to NCBI (SAMN30088480, SAMN30088482). All supporting data and materials are available in the *GigaScience* database, GigaDB [100].

## Additional Files

**Supplementary Fig. S1**. The *k*-mer depth distribution curve (a: male, b: female). Horizontal axis: *k*-mer depth. Vertical axis: frequency of *k*-mer at specified depth. Blue area represents observed *k*-mer depth distribution. Area under red line represents low-frequency *k*-mers, which are identified as sequencing errors. Reliable *k*-mers are shown under the black line, which are used to estimate genome size. Vertical dashed lines show peak positions of *k*-mer depth distribution. Nonrepeat fraction is shown under yellow line.

**Supplementary Fig. S2**. Chromosome-level assembly of the male and female cobia genome using Hi-C data (a: male, b: female). Heatmap of contact metrics generated from mapping of Hi-C reads to genome sequences. The darker the red, the stronger the interaction. Interaction intensity of intrachromosomes is stronger than that of interchromosomes. Boundaries of chromosomes are obvious.

**Supplementary Fig. S3**. Gene family comparison of cobia and other fish, and single-copy orthologs were used to construct the phylogenetic tree. Statistics of orthologous families for *R. canadum* (cobia), *T. ovatus* (pompano), *S. lalandi* (yellowtail amberjack), *E. naucrates* (live sharksucker), *C. milii* (elephant shark), *L. crocea* (large yellow croker), *D. rerio* (zebrafish), *O. latipes* (medaka), *G. morhua* (Atlantic cod), and *E. lanceolatus* (giant grouper). Single-copy orthologs represent single-copy genes in the family. Multiple-copy orthologs represent genes with multiple copies in the family. Other orthologs represent the gene families not in all species. Unique paralogs mean that genes belong to the families that existed in only 1 species. Unclustered genes represent the genes that could not be clustered into gene families.

**Supplementary Fig. S4**. Venn diagram showing shared orthologous groups for *R. canadum* (cobia), *S. lalandi, L. crocea*, and *E. lanceolatus*. A total of 11,968 gene families were shared by the 4 species. In addition, 255 gene families were specific in *R. canadum*.

**Supplementary Fig. S5**. Phylogenetic tree. Single-copy families were chosen to construct a phylogenetic tree. *C. milii* is the outgroup.

**Supplementary Fig. S6**. Genome comparisons shows 24 chromosomes of *R. canadum* with a one-to-one relationship with 24 chromosomes of the other 3 Carangiformes, including 2 Carangidaes of *T. ovatus* and *S. lalandi*, as well as 1 Echeneidae of *E. naucrates*. (A) *R. canadum* and *E. naucrates* (live sharksucker). (B) *R. canadum* and *T. ovatus* (pompano). Right half round: chromosomes of *R. canadum*. Left half round: chromosomes of *E. naucrates* and *T. ovatus*.

**Supplementary Fig. S7**. The metabolism xenobiotics by the cytochrome P450 pathway.

**Supplementary Table S1**. Statistics of raw reads and clean reads generated from stLFR.

**Supplementary Table S2**. Statistics of raw reads and clean reads generated from Hi-C.

**Supplementary Table S3**. Summary of male and female cobia genome assembly and annotation.

**Supplementary Table S4**. Scaffolding statistics based on Hi-C data for each chromosome of male and female cobia genome.

**Supplementary Table S5**. Quality assessment of female and male genome assembly completeness and gene sets with the BUSCO tool (using Actinopterygii gene set of BUSCO database).

**Supplementary Table S6**. Functional annotation of predicted protein-coding genes of the male and female cobia genome.

**Supplementary Table S7**. Statistics of repeat elements in the male cobia genome.

**Supplementary Table S8**. Statistics of repeat elements in the female cobia genome.

**Supplementary Table S9**. Noncoding RNA prediction in the male and female cobia genome.

**Supplementary Table S10**. Statistics of male and female cobia resequencing. See separate Excel sheet.

**Supplementary Table S11**. A total of 162 SNPs strongly associated with sex at the peak region on Chr18 detected by genome-wide association studies. See separate Excel sheet.

**Supplementary Table S12**. GWAS detected suggestive signals of sex association at Chr4, Chr5, and Chr17.

**Supplementary Table S13**. Genes annotated in reassembled male chromosome 18 (MChr18). See separate Excel sheet.

**Supplementary Table S14**. In total, 231 SNPs strongly associated with sex at the peak region on Chr18 detected by genome-wide association studies. See separate Excel sheet.

## Abbreviations

BUSCO: Benchmarking Universal Single-Copy Orthologs; EDC: endocrine-disrupting compound; Gb: gigabase; GWAS: genome-wide association study; Mya: million years ago; NCBI: National Center for Biotechnology Information; PE: paired-end; PPI: protein–protein interaction; SNP: single-nucleotide polymorphism; stLFR: single-tube long fragment read; tRNA: transfer RNA; WGRS: whole-genome resequencing.
