# Supplementary material for: Exploring the cobia (Rachycentron canadum) genome: unveiling putative male heterogametic regions and identification of sex-specific markers
Source: Gigascience. 2024 Jul 12;13:giae034. doi: 10.1093/gigascience/giae034 (PMC11240236; doi:10.1093/gigascience/giae034)
Supplement: giae034_GIGA-D-23-00328_Original_Submission [file giae034_giga-d-23-00328_original_submission.pdf]

GigaScience

Genomic investigation of the cobia (Rachycentron canadum) reveals putative male heterogametic regions on chromosome 18

--Manuscript Draft--

**Manuscript Number:**	GIGA-D-23-00328
**Full Title:**	Genomic investigation of the cobia (Rachycentron canadum) reveals putative male heterogametic regions on chromosome 18
**Article Type:**	Research
**Funding Information:**	Open Blue Sea Farms Panama S.A. Cobia (OBSF), James Cook University Singapore (JCUS) and University of Chile (UoC) — Dr Jose Domingos
**Abstract:**	Background: Cobia (Rachycentron canadum) is the only member of the Rachycentridae family and exhibits considerable sexual dimorphism in growth rate. Sex determination in teleosts has been a long-standing basic biological question, and the molecular mechanisms of sex determination/differentiation in cobia species are completely unknown.<br>Results: Here, we reported two high-quality, chromosome-level annotated male and female cobia genomes with assembly sizes of 586.51 Mb (contig/scaffold N50: 86.0 kb/24.3 Mb) and 583.88 Mb (79.9 kb/22.5 Mb), respectively. Synteny inference among perciform genomes revealed that cobia and the remora Echeneis naucrates were sister groups. Further, whole genome resequencing of 31 males and 60 females, genome-wide association study (GWAS) and sequencing depth analysis identified three short male-specific regions within a 10.7 kb continuous genomic region on male chromosome 18, which hinted at an undifferentiated sex chromosome system with a XX/XY mode of sex determination in cobia. Importantly, the two only genes within/between the male-specific regions, epoxide hydrolase 1 (ephx1, renamed cephx1y), and transcription factor 24 (tcf24, renamed ctcf24y), showed testis-specific/biased gene expression, whereas their counterparts cephx1x and ctf24x, located in female chromosome 18, were similarly expressed in both sexes. In addition, male-specific PCR targeting the cephx1y gene revealed that this genomic feature is conserved in cobia populations from Panama, Brazil, Australia, and Japan.<br>Conclusion: The first comprehensive genomic survey presented here is a valuable resource for future studies on cobia population structure and dynamics, conservation, and evolutionary history. Furthermore, it establishes evidence of male heterogametic regions with two genes playing a potential role in the sex determination of the species, and provides further support for the rapid evolution of sex-determining mechanisms in teleost fish.
**Corresponding Author:**	Xueyan Shen<br>James Cook University - Singapore Campus<br>Singapore, SINGAPORE
**Corresponding Author Secondary Information:**	
**Corresponding Author's Institution:**	James Cook University - Singapore Campus
**Corresponding Author's Secondary Institution:**	
**First Author:**	Xueyan Shen
**First Author Secondary Information:**	
**Order of Authors:**	Xueyan Shen
	Jie Hu
	José Yáñez
	Giana Bastos Gomes
	Zhi Weng Josiah Poon
	Derick Foster

Powered by Editorial Manager® and ProduXion Manager® from Aries Systems Corporation

	Jorge Alarcon
	Libin Shao
	Xinyu Guo
	Yunchang Shao
	Roger Huerlimann
	Chengze Li
	Evan Goulden
	Kelli Anderson
	Guangyi Fan
	Jose Domingos
**Order of Authors Secondary Information:**	
**Additional Information:**	
**Question**	**Response**
Are you submitting this manuscript to a special series or article collection?	No
**Experimental design and statistics**<br><br>Full details of the experimental design and statistical methods used should be given in the Methods section, as detailed in our Minimum Standards Reporting Checklist. Information essential to interpreting the data presented should be made available in the figure legends.<br><br>Have you included all the information requested in your manuscript?	Yes
**Resources**<br><br>A description of all resources used, including antibodies, cell lines, animals and software tools, with enough information to allow them to be uniquely identified, should be included in the Methods section. Authors are strongly encouraged to cite Research Resource Identifiers (RRIDs) for antibodies, model organisms and tools, where possible.<br><br>Have you included the information requested as detailed in our Minimum Standards Reporting Checklist?	Yes

Powered by Editorial Manager® and ProduXion Manager® from Aries Systems Corporation

**Availability of data and materials**<br><br>All datasets and code on which the conclusions of the paper rely must be either included in your submission or deposited in publicly available repositories (where available and ethically appropriate), referencing such data using a unique identifier in the references and in the “Availability of Data and Materials” section of your manuscript.<br><br>Have you have met the above requirement as detailed in our Minimum Standards Reporting Checklist?	Yes

Powered by Editorial Manager® and ProduXion Manager® from Aries Systems Corporation

Manuscript Click here to access/download;Manuscript;Manuscript.docx

Genomic investigation of the cobia (*Rachycentron canadum*) reveals putative male heterogametic regions on chromosome 18

Xueyan Shen[1#*], Jie Hu[2#], José M. Yáñez[3], Giana Bastos Gomes[4], Zhi Weng Josiah Poon[5], Derick Foster[6], Jorge F. Alarcon[6], Libin Shao[2], Xinyu Guo[2], Yunchang Shao[7,8,9], Roger Huerlimann[10], Chengze Li[10], Evan Goulden[11], Kelli Anderson[11], Guangyi Fan[2,7,12*], Jose A. Domingos[1,13*]

[1]Tropical Futures Institute, James Cook University Singapore, 149 Sims Drive, 387380 Singapore

[2]BGI-Qingdao, BGI-Shenzhen, Qingdao, Shandong, 266555, China

[3]Facultad de Ciencias Veterinarias y Pecuarias, Universidad de Chile, Av. Santa Rosa 11735, La Pintana, 8820808 Santiago, Chile

[4]Temasek Life Sciences Laboratory, 1 Research Link, National University of Singapore, 117604, Singapore

[5]James Cook University, Singapore, 149 Sims Drive, 387380, Singapore

[6]Open Blue Sea Farms, Panama City, Panama

[7]China National GeneBank, BGI-Shenzhen, Shenzhen, Guangdong, 518120, China

[8]State Key Laboratory of Quality Research in Chinese Medicine, Institute of Chinese Medical Sciences, University of Macau, Macao 999078, China

[9]Geogia Tech Shenzhen Institute (GTSI), Tianjin University, Shen Zhen 518067, China

[10]Marine Climate Change Unit, Okinawa Institute of Science and Technology (OIST), 1919-1 Tancha, Onna-son Okinawa, 904-0495, Japan

[11]Department of Agriculture and Fisheries, Queensland Government, Bribie Island Research Centre, P.O. Box 2066, Woorim, QLD 4507, Australia.

[12]BGI-Shenzhen, Shenzhen, Guangdong, 518083, China

[13]Centre for Sustainable Tropical Fisheries and Aquaculture, James Cook University, 1 James Cook Drive, Townsville QLD 4811, Australia

[#]These authors contributed equally to this work.

[*]Correspondence to: Xueyan Shen; Email: Xueyan.shen@jcu.edu.au; Tel: Guangyi Fan; Email: fanguangyi@genomics.cn; Jose A. Domingos; Email: jose.domingos1@jcu.edu.au

Abstract

Background: Cobia (*Rachycentron canadum*) is the only member of the *Rachycentridae* family and exhibits considerable sexual dimorphism in growth rate. Sex determination in teleosts has been a long-standing basic biological question, and the molecular mechanisms of sex determination/differentiation in cobia species are completely unknown.

Results: Here, we reported two high-quality, chromosome-level annotated male and female cobia genomes with assembly sizes of 586.51 Mb (contig/scaffold N50: 86.0 kb/24.3 Mb) and 583.88 Mb (79.9 kb/22.5 Mb), respectively. Synteny inference among perciform genomes revealed that cobia and the remora *Echeneis naucrates* were sister groups. Further, whole genome resequencing of 31 males and 60 females, genome-wide association study (GWAS) and sequencing depth analysis identified three short male-specific regions within a 10.7 kb continuous genomic region on male chromosome 18, which hinted at an undifferentiated sex chromosome system with a XX/XY mode of sex determination in cobia. Importantly, the two only genes within/between the male-specific regions, epoxide hydrolase 1 (*ephx1*, renamed *cephx1y*), and transcription factor 24 (*tcf24*, renamed *ctcf24y*), showed testis-specific/biased gene expression, whereas their counterparts *cephx1x* and *ctf24x*, located in female chromosome 18, were similarly expressed in both sexes. In addition, male-specific PCR targeting the *cephx1y* gene revealed that this genomic feature is conserved in cobia populations from Panama, Brazil, Australia, and Japan.

Conclusion: The first comprehensive genomic survey presented here is a valuable resource for future studies on cobia population structure and dynamics, conservation, and evolutionary history. Furthermore, it establishes evidence of male heterogametic regions with two genes playing a potential role in the sex determination of the species, and provides further support for the rapid evolution of sex-determining mechanisms in teleost fish.

Keywords: Chromosome-level genome; Cobia; Molecular sex markers; stLFR, Hi-C, PacBio sequencing

Introduction

Cobia (*Rachycentron canadum)* is a large migratory pelagic fish with geographic distribution in tropical and subtropical waters worldwide with the exception of the eastern Pacific Ocean [1]. It is a promising marine fish species with great aquaculture potential due to its desirable traits, such as excellent quality fillets, easy adaptation to captivity, high survival rates, tolerance to variations in temperature and salinity, and high growth rate [2]. The species has been farmed in many countries around the world including China, Taiwan, and Hong Kong, and more recently expanding to Australia, Vietnam, and the American continent (USA, Brazil, Panama, Belize etc.) [3-5]. Cobia exhibits a strong sexually dimorphic growth [6-8]. Females grow faster than males both in body length and weight, creating considerable differences between sexes in which females can be double the size of males at similar developmental stages [1]. In light of this, it is widely acknowledged that monosex female breeding through artificial sex control can significantly boost cobia aquaculture yields [2]. The morphological characteristics (i.e., secondary sexual traits) used for sexing are usually only observed after sexual maturation, and thus are not useful for sexing juvenile fish. Nevertheless, it is often useful to know the sex of juveniles, e.g. in aquaculture breeding programs. Relying solely on cobia morphology is not enough to distinguish their sex at any developmental stage, including post-sexual maturity. Furthermore, a reliable approach for distinguishing cobia's genotypic sex has yet to be established.

Teleost fish exhibit a remarkable diversity and complexity of sex-determining mechanisms, and sex differentiation involves the expression of a considerable number of genes in a spatial and temporal order [9]. Sexual determination mechanisms in fish may involve: genetic control (e.g., heterogamety for males (XY) or females (ZW)), multiple sex-determining chromosomes and genes (X1X1X2X2/X1X2Y, XX/XY1Y2), environmental triggers (e.g., temperature, pH, behaviour, population density and social status) [10-12], epigenetic sex determination and hermaphroditism [13-16]. Currently, multiple master sex determination genes have been reported in various fish species (for review see [17], such as *sdy* in rainbow trout (*Oncorhynchus mykiss*) [18], *dmy/dmrt1* in Japanese rice fish/medaka (*Oryzias latipes*) [19, 20], *amhy/amhby* in Patagonian pejerrey (*Odontesthes hatcheri*) [21], Nile tilapia (*Oreochromis niloticus*) [22], threespine stickleback [23, 24] and northern pike

(*Esox lucius*) [25], *hsd17b1* in yellowtail spp. [26], and *bcar1* in channel catfish (*Ictalurus punctatus*) [27].

The even representation of males and females within cobia populations suggests that there is a genetic system (i.e., a master gene) driving sex determination, and the species is considered gonochoristic [1]. Rare occurrences of intersex individuals have been reported in India [28] and Australia [3], with the latter supposedly attributed to the presence of endocrine disrupting compounds in the water. Unfortunately, limited knowledge is available on the molecular mechanisms of sex determination and differentiation in this species. To date, there have been no reported sex chromosomes, sex-determining regions, or sex determination genes in cobia. Furthermore, cytologically there are no distinguishable sex chromosomes observed between genders, as male and female cobia show the same diploid number (2n = 48) and the same karyotype morphology [2, 29]. Hence, the lack of reliable genotypic and phenotypic approaches for distinguishing the sex of cobia presents a significant hurdle for practitioners seeking to optimize broodstock management, conduct molecular selective breeding, as well as advance the conservation of the species. Consequently, it is crucial to explore the genetic underprinnings of sex determination and develop molecular markers that permit non-invasive and early sexing of cobia individuals.

Cobia is the extant monotypic member of family *Rachycentridae*, order *Carangiformes,* which consists of six families. Three of these families (i.e., *Rachycentridae*, *Coryphaenidae* and *Echeneidae*) are within the super family *Echeneoidea* that comprise a monophyletic grouping [30, 31]. *R. canadum* was assumed to be closely related (sister groups) to the remoras (*E. naucrates*), within the family *Echeneidae,* based on the morphology (form, colour and fin shape) of juveniles [31]. However, osteological examinations revealed a greater likelihood of sister groups between *R. canadum* and *Coryphaena* based on the larval morphology [31]. In addition, a phylogenetic analysis of 138 putatively informative characters of eleven species (including *R. canadum*) resulted in a single most parsimonious tree and showed that *Rachycentridae* is the sister-group to *Echeneidae* [32]. While phylogenetics of *Carangoid* based on the complete mitochondrial DNA supported that the relationship between *R. canadum* and mahi-mahi (*Coryphaena hippurus)*, within family *Coryphaenidae*, was the closest [33]. Whereas these studies have shed light on *R. canadum* phylogeny in relation to other clades, the question of whether it is more closely related to *Coryphaenidae* or

Echeneidae still remains controversial. Therefore, further studies are required to elucidate the phylogenetic relationships of *R. canadum* within the order *Carangiformes* and understand its evolutionary history.

Genomic resources for cobia are currently extremely limited, hindering a better understanding of the genetic basis of sex determination and differentiation, as well as the molecular mechanisms of remarkable sexual dimorphisms in this unique fish species. Therefore, the goals of this study were to: i) assemble the first male and female chromosome-level reference genome for cobia; ii) identify candidate sex-linked genomic regions, putative sex-determining genes, as well as develop affordable and rapid male-specific DNA markers to determine the genetic sex of cobia; and iii) elucidate the phylogenetic relationship between cobia and other teleosts via genome synteny.

Results

Assembly and annotation of chromosome-level male and female cobia genomes

We sequenced one male and one female cobia using single tube long fragment read (stLFR) and Hi-C technologies, each sex with over 285-fold genome coverage (Supplementary Tables S1,S2). *De novo* genome assembly was performed on 87.07 Gb and 78.12 Gb of clean stLFR reads separately generated for male and female, respectively (Supplementary Table S1). A 586.23 Mb of the male genome was assembled with a contig/scaffold N50 of 86.0 kb/10.3 Mb (Supplementary Table S3), which is close to the 585.72 Mb estimate from k-mer analysis (Supplementary Fig. S1a). The genome assembly size for the female was 583.56 Mb (accounting for 99.2% of the k-mer estimated 588.46 Mb) with a contig/scaffold N50 of 79.9 kb/6.3 Mb (Supplementary Table S3 and Supplementary Fig. S1b). Detailed information on the estimation of the genome size based on K-mer analysis is shown in Supplementary File Note 1. To further improve the genome assembly and anchor the scaffold sequences to chromosomes, we generated 81.5 Gb and 103.1 Gb Hi-C data for the male and female, respectively (Supplementary Table S2). By incorporating the Hi-C data, 586.51 Mb of the male genome was assembled with a scaffold N50 of 24.3 Mb, whereas the female genome assembly size was 583.88 Mb with scaffold N50 of 22.5 Mb (Supplementary Table S3). A total of 563.06 Mb (96.00% of the assembly) of the male and 537.27 Mb (92.02% of the assembly) of the female genome sequence were ordered and oriented into 24 pseudo-chromosomes,

respectively (Fig. 1a, Supplementary Fig. S2 and Table S4). This outcome was consistent with the previous report on cobia karyotype (2n = 48) [29]. All 24 chromosomes of the male and female genomes showed a clear one-to-one syntenic relationship (Fig. 1a and Supplementary Table S4). The quality of the two genome assemblies was assessed in two aspects: a) complete and single copy BUSCO scores of 94.2% (male) and 93.8% (female) (Supplementary Table S5); and b) an average of 96.45% and 97.88% RNASeq reads from gonadal tissues of cobia [8] could be mapped to the male and female genome assemblies, respectively. These results indicate that the assembled genomes were high quality.

A total of 21,604 and 21,688 protein-coding genes were separately annotated in the male and female genome assembly (Supplementary Table S3), and over 99% of them were annotated by functional database (Supplementary Table S6). The BUSCO evaluation of the protein sequences identified 93.1% and 92.6% of complete single copy genes for the male and female genome assembly, respectively (Supplementary Table S5). About 11.08% and 11.55% of the assembled male and female genome was annotated and identified as repetitive elements (Supplementary Tables S7, S8). We also identified 1,304 and 1,289 non-coding RNAs, with a total length of 116.9 kb and 117.0 kb in the male and female genomes, respectively (Supplementary Table S9).

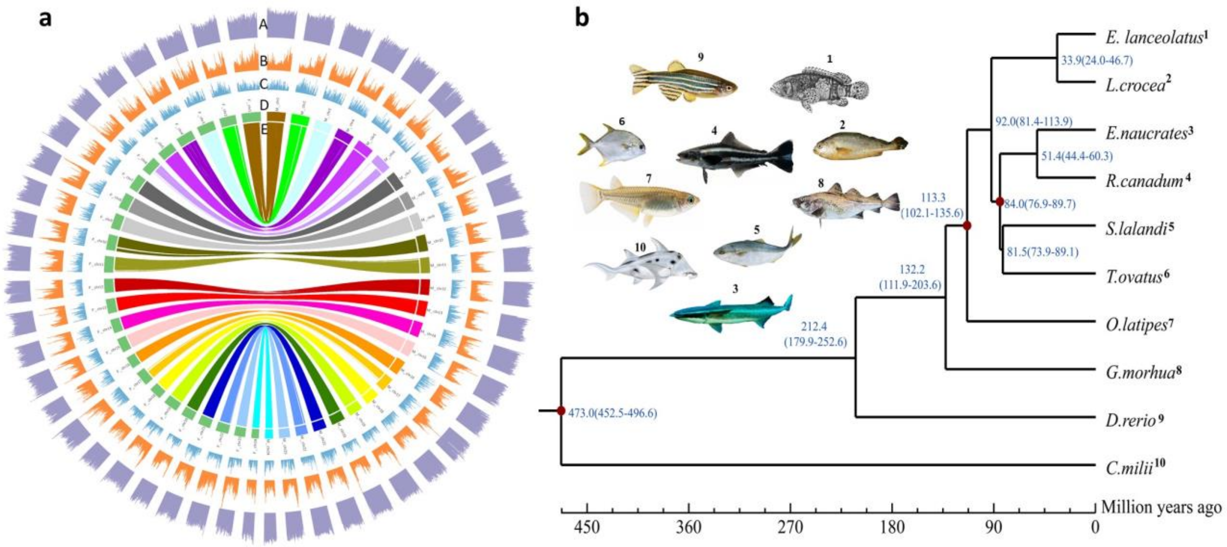

Figure 1: Overview of male and female cobia genome features. (a) Landscape of the 24 assembled cobia chromosomes. From the outer to the inner: A) GC_content, B) Transposable element content density, C) Gene density, D) Chromosomes, E) Syntenic relationship of female (left of the circle) and male (right of the circle) chromosomes. (b) Phylogenetic tree of 10 vertebrate genomes constructed using 572 single copy orthologous genes. The numbers (blue) on the branches represent the estimated divergence time in million years ago (Mya). Time span in brackets were the 95% confidence interval of divergence time, and red circles indicate the calibration time from fossil. All nodes had support values of 100%.

Phylogenetic construction and evolution analysis reveals *R. canadum* and *E. naucrates* as sister groups

To investigate the evolutionary relationship of cobia (*R. canadum*) and related teleosts, a phylogenetic tree was constructed using 572 single-copy orthologous genes of cobia and nine other fish species (Supplementary Fig. S3,S4). Of which, three were Carangiformes including two Carangidaes of *T. ovatus* (pompano) and *S. lalandi* (yellowtail amberjack), as well as one Echeneidae of *E. naucrates* (remora or live sharksucker). The remaining six were *C. milii* (elephant shark), *L. crocea* (large yellow croker), *D. rerio* (zebrafish), *O. latipes* (medaka), *G. morhua* (Atlantic cod), and *E. lanceolatus* (giant grouper). The phylogenetic relationship showed that *R. canadum* clustered within the order Carangiforme, together with *E. naucrates*, *S. lalandi* and *T. ovatus,* which was consistent with results reported previously (Johnson, 1984) and confirmed that *R. canadum* and *E. naucrates* were sister groups (Fig. S5). From the estimates of divergence time, the ancestor of *R. canadum* separated from the ancestor of *E. naucrates* approximately 51.4 million years ago (Mya). The ancestor of *R. canadum* and *E. naucrates* separated from the ancestor of *S. lalandi* and *T. ovatus* approximately 84.0 Mya (Fig. 1b). In addition, the 24 pseudo-chromosomes of cobia had a clear one-to-one relationship to *E. naucrates* (Supplementary Fig. S6a), while seven chromosomes (6, 7, 10, 13, 14, 15 and 17) of *R. canadum* were observed to have a hit to two or three chromosomes of *T. ovatus* (Supplementary Fig. S6b). Unfortunately, the chromosomal level genome of *S. lalandi* was not available, so no syntenic relationship was explored between *R. canadum* and *S. lalandi.*

Characterization of sex-specific regions in cobia

To locate the sex-specific genomic region(s) of cobia, a total of 2,681 Gb of filtered whole genome resequencing (WGRS) data were generated from 91 individuals (31 males and 60 females), with an average of ~49-fold depth per sample (Supplementary Table S10). Using the male genome as reference, an average mapping rate of 99.0% per sample was obtained (Supplementary Table S10). In total, 551,838 filtered single nucleotide polymorphisms (SNPs) were detected. The genome-wide association analysis (GWAS) using the male genome as reference revealed a single peak (-log p-values of up to 244.37) with 162 SNPs significantly associated with sex, spanning over a region of ~4.04 Mb (559.54 kb to 4.59 Mb) on male chromosome 18 (MChr18) (Fig. 2a, 2b and Supplementary Table S11). Most importantly, the 162 strongly sex-associated SNPs showed the same pattern where all thirty-one males were

heterozygous, but homozygous for all sixty females (Supplementary Table S11). These results hinted at a male heterogametic or XX/XY model of sex determination, with a fully sex-linked region on MChr18. In addition, the principal component analysis (Fig. 2c) and a neighbour-joining tree (Fig. 2d) calculated using the SNPs from MChr18 showed that male and female individuals clustered into two distinct groups. Moreover, the relative component of genetic differentiation (estimated as *Fst*) between males and females further confirmed the region detected by GWAS (Fig. 2e). Hence, both the GWAS and *Fst* scan, which takes genetic structure into consideration, consistently identified a peak genomic region on MChr18, showing the highest probability as a sex-associated region in cobia. While GWAS indicated potential association signals in three other genomic areas (MChr4, MChr5, and MChr17) (Fig. 2a), a more detailed examination revealed that the SNP genotypes within these regions did not consistently exhibit heterozygosity in males and homozygosity in females. Furthermore, the resequencing data showed comparable coverage of these regions in both males and females. Consequently, it's improbable that these regions play an important role in sex determination.

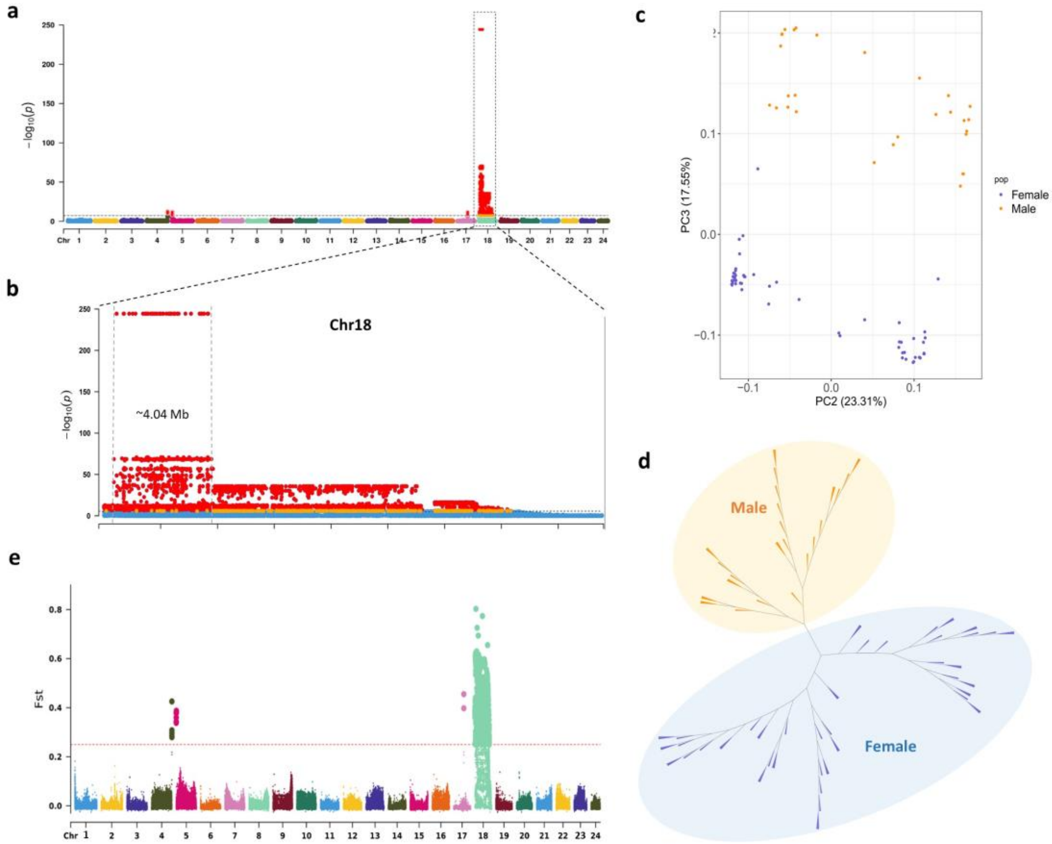

Figure 2: Genome-wide distribution of SNPs from 31 males and 60 females. (a) Manhattan plot showing –log10 p-value of each SNP from the GWAS investigating sex-associated regions on the cobia genome. The horizontal line indicates the genome-wide significance threshold -log10(P)= 7.7. (b) The SNP distribution on

Chr18. The 162 SNPs significantly associated with sex spanning over a region of ~4.04 Mb (559.54 kb to 4.59 Mb). (c) Principal component analysis of ninety-one individuals using SNPs. (d) Phylogenetic tree showing relationships of females (blue) and males (orange). (e) Genome-wide scan of fixation index (*Fst*) matching the results from the GWAS.

The genome-wide difference of sequencing depth between males and females was also analysed to identify the sex-specific region(s) in cobia. By investigating the mean depth (sites depth/average depth), the sex-linked region identified above was further narrowed down on MChr18. Unfortunately, an unacceptable number of gaps with variable length were observed within the region and the flanking regions. To improve the contiguity of this sex-associated region, we performed PacBio HiFi sequencing of the DNA from a male cobia individual, which rendered 1,170,581 highly accurate PacBio long reads with an average length of 16.1 kb (longest read: 40.4 kb; N50 = 15.9 kb) totalling 18.86 Gb; representing 32X coverage of the male genome. This set of PacBio reads were assembled first and then aligned to the MChr18. A large scaffold of 15.99 Mb from the PacBio genome assembly which contained the above identified sex-associated region was further reassembled with MChr18. The result was a new MCh18 with a total length of 21.98 Mb, and 843 genes (68 more than in the original MCh18) were detected from its re-annotation (Supplementary Table S12). Most importantly, all the gaps presenting within the sex-linked region and its flanking regions in the original MChr18 were fully filled. GWAS analysis was carried out on the newly assembled MChr18, which detected 232 SNPs significantly associated with sex in a single peak. Consistently, all 232 SNPs showed that all 31 males were heterozygous, but all 60 females were homozygous (Supplementary Table S13). Interestingly, further sequencing depth analysis revealed that three short male-specific regions of Y1 (400 bp; 3,187,350 to 3187750 bp), Y2 (1,100 bp; 3,195,150 to 3,196,250 bp) and Y3 (1,000 bp; 3,197,050 to 3,198,050 bp) within a continuous region of 10.7 kb were discovered within the sex region, which showed no WGRS reads mapped from 60 females (corresponding depth of zero for females), while with mean depth of 0.5 (haploid copy specific to males) in 31 males (Fig. 3a, 3b), suggesting that the Y1, Y2 and Y3 are putative male determining regions that could contain the candidate master sex-determining genes. In addition, the θπ value analysis showed that the divergence mainly came from the male group (Fig. 3c).

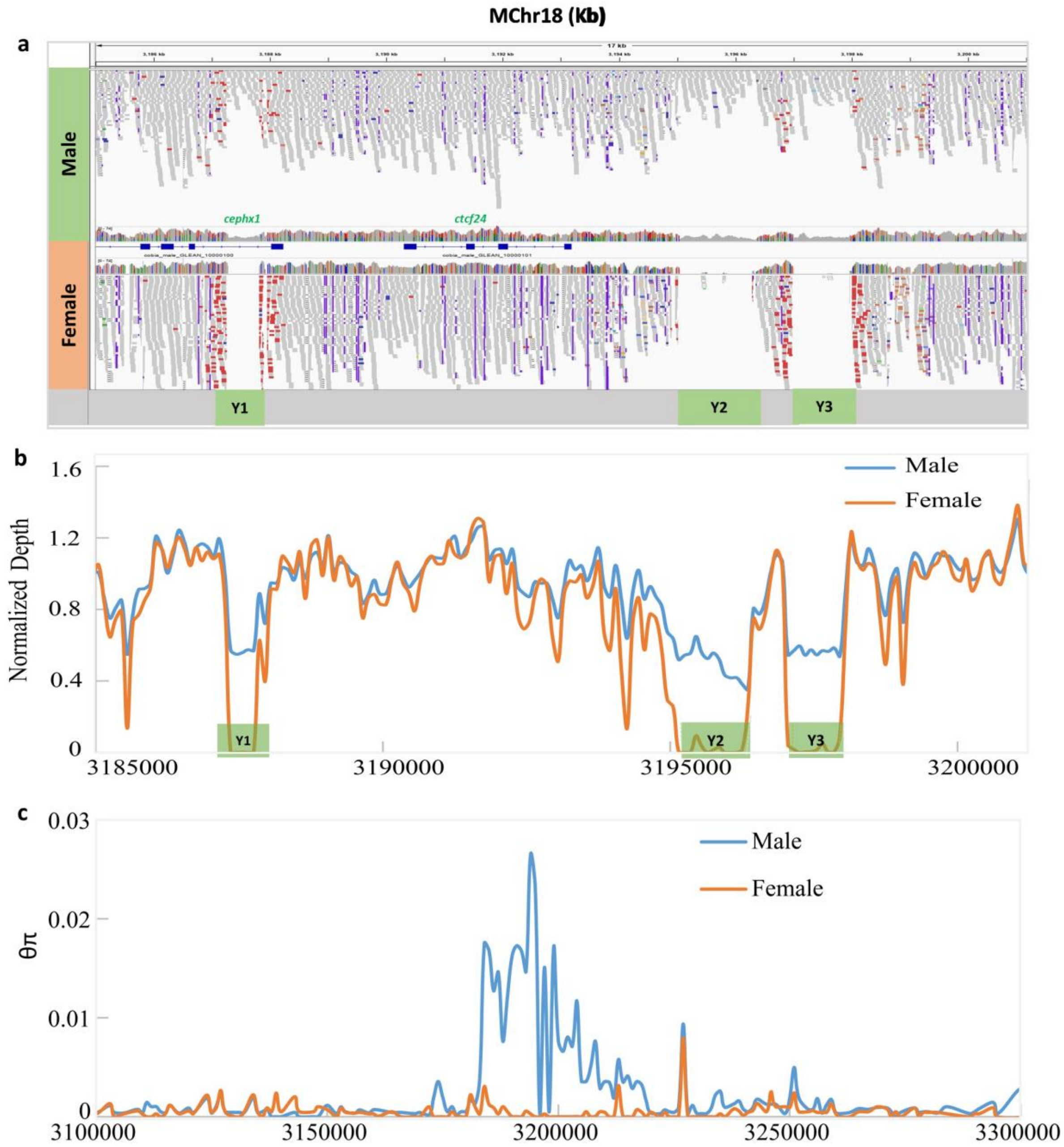

Figure 3: (a) Alignments of the stLFR reads from male and female individuals to the sex-associated regions. (b) Average depth normalized per group. For both figure (a) and (b)**,** the letters of Y1, Y2 and Y3 stand for the distinct regions between males and females. (c) Genetic diversity of sex-determining region. Blue line indicates the female group, and yellow line represents the male group.

The c*ephx1y* and *ctcf24y:* the putative drivers of cobia sex determination

We further scanned the 10.7 kb sex-associated region on MChr18. A short insertion (540 bp within the 6th intron region) in a functionally annotated gene of epoxide hydrolase 1_*ephx1* (3,184,084 to 3,188,235 bp) was identified in the male-specific region Y1. There were no genes detected in region Y2 and Y3 (Fig. 3a). However, another gene, transcription factor 24_*tcf24* (3,190,353 to 3,193,193 bp), was detected between Y1 and Y2 (Fig. 3a). These two

genes, especially the *ephx1*, were considered of high interest for male function in cobia. In addition, both genes were also found in the homologous female chromosome of FChr18 (19.29 Mb). Alignment of *ephx1* and *tcf24* genomic sequences in MChr18 and FChr18 revealed a high nucleotide identity of 96.6% and 95.9%, respectively. InDel (insertion-deletion) variants with variable length and SNPs also existed in both gene (coding regions and introns) comparison groups (Supplementary File: Genomic DNA sequence alignment of *ephx1* and *tcf24*). The two genes on MChr18 were termed cobia *ephx1y* (*cephx1y*) and cobia *tcf24y* (*ctcf24y*), and *cephx1x* and *ctcf24x* for Fchr18. As nucleotide sequence divergence impacts protein sequence, gene structure predictions were performed for both genes. The results showed that *cephx1x* spans about 3.78 kb and consists of seven introns and eight exons (Fig. 4a1) encoding 455 amino acids (Fig. 4b1). However, only six introns and seven exons were detected for *cephx1y* with a total length of 4.15 kb*,* which showed one exon (VII) absent (Fig. 4a1) encoding 416 amino acid residues (Fig. 4b1).

The nucleotide identity between exon sequences of *cephx1x* and *cephx1y* ranged from 95.1% to 98.3% with an average of 97.3% (Fig. 4a1). A closer look at *cephx1x* and *cephx1y* revealed that the largest sequence differences were two InDels of 165-bp of X-specific insertion and 540-bp of Y-specific insertion in the non-coding regions (Fig. 4a1), while the remaining were randomly distributed SNPs and short InDels (Supplementary file). It is noteworthy that the Y1 region was in fact the male-specific fragment specifically inserted in the sixth intron of the *cephx1y* (Fig. 4a1 and Supplementary file). In terms of *tcf24,* the *ctcf24y* contained the same number of exons (four) and introns (three) as *ctcf24x* (Fig. 4a2), *ctcf24y* spans 2,840 bp and encodes 202 amino acids, while *ctcf24x* has 3,024 bp with a translated protein product of 204 amino acids (Fig. 4b2). The observed sequence identity in exons was 96.9 to 98.8%. There was also a large 220-bp X-specific insertion in the third intron of *ctcf24x,* and several small InDels and SNPs were also detected between them (Fig. 4b1 and Supplementary file). Moreover, we built a structural model for both genes. The Ephx1 is a protein coding gene, the Cephx1y protein folds is essentially identical to Cephx1x (Cα root-mean-square deviation of 1.108Å). While the Cephx1x had an extra 41-amnio-acid helix-turn-helix domain (missing in Cephx1y), which plays an important role in the stability of the protein (Fig. 4c1). In addition, the amino acid alignment of the two *ephx1* genes of cobia and other fish like *E. naucrates*, *E. lanceolatus* and *Seriola dumerili* revealed that the loss of the helix-

turn-helix domain existed only in the Cephx1y (Supplementary File). Both cobia *tcf24* counterparts lack a fixed or ordered three-dimensional structure, and a total of nine amino acid differences at six sites were detected between Ctcf24x and Ctcf24y (Fig. 4c2).

A further investigation of the expression pattern of *ephx1* and *tcf24* by examining the cobia gonadal transcriptome [8] showed that both *cephx1y* and *ctcf24y* were significantly differentially expressed between males and females (Fig. 4d). The *ctcf24y* was more highly expressed in testis (FPKM: 7.27) than ovaries (FPKM: 0.28), with the Log_2 (Fold Change) of ovary/testis of -4.8. In addition, the expression of *cephx1y* was observed in all five testis samples ($1.5 \leq$ FPKMs ≤ 6.3), but only in one of five ovary samples ($0 \leq$ FPKMs ≤ 0.02), with the $log_2FC = -9.5$; indicating this gene was nearly exclusively expressed in male cobia (Fig. 4d). The *cephx1x* and *ctcf24x* showed no significant differential expression between testes and ovaries. In addition, PPI network analysis showed that the *cephx1y* interacts directly and significantly with three cyp1 genes: *cyp1a1, cyp1b1* and *cyp1d1* (Fig. 4e). Testis-specific expression was observed for *cyp1a1*, while *cyp1b1* was overexpressed in the ovary, and *cyp1d1* was absent [8]. The *ephx1* gene together with *cyp1a1* and *cyp1b1* are involved in the metabolism of xenobiotics through the cytochrome P450 pathway (Supplementary Fig. S7). In addition, the *cephx1y* also interacts indirectly with *hsd17b1* and three other CYP components: *cyp19a1a* (gonadal aromatase), *cyp19a1b* (brain aromatase) and *cyp3a65* (predicted to enable steroid hydroxylase activity in zebrafish).

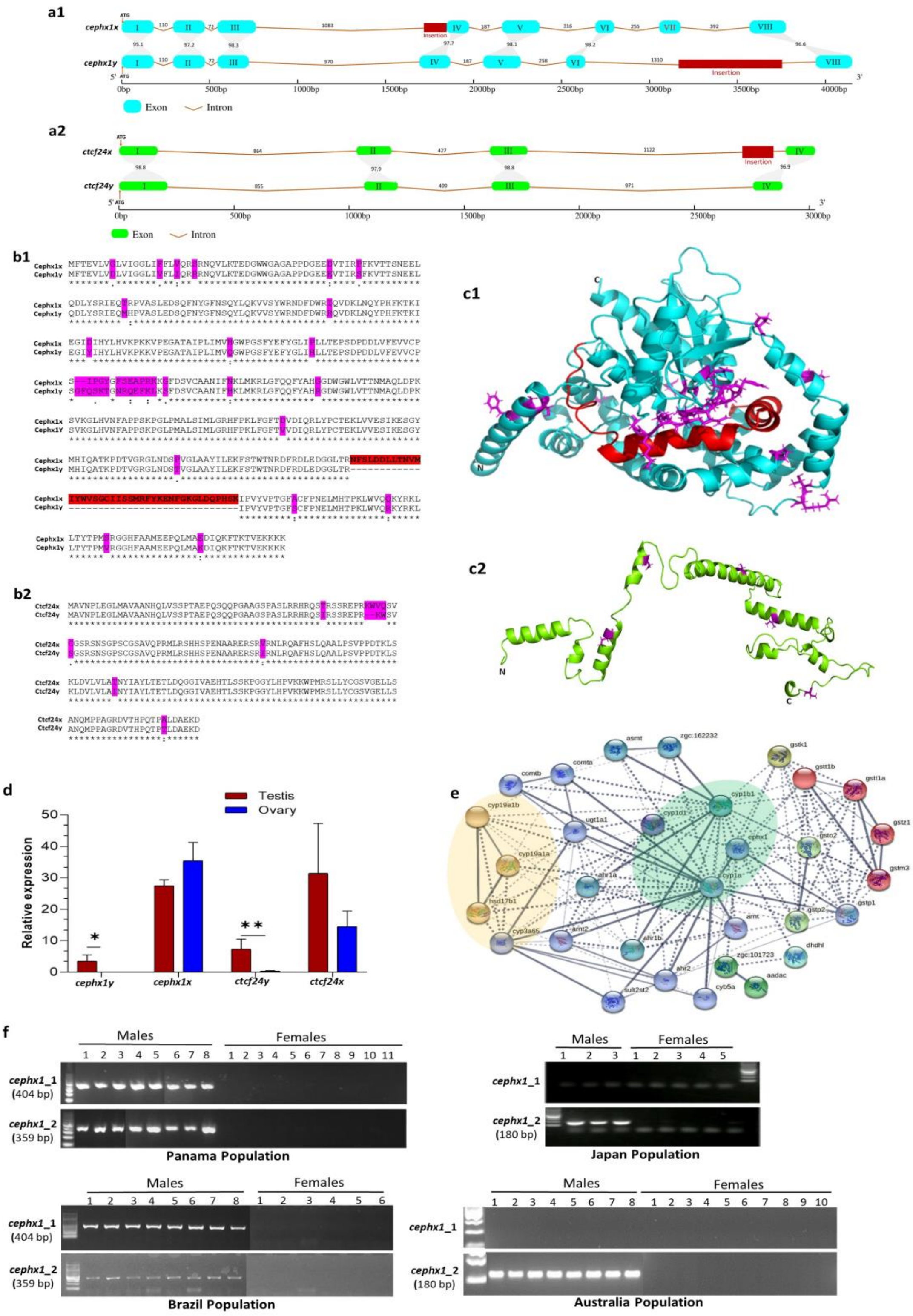

Figure 4: (a) Schematic representation gene structure of *cephx1y* and *cephx1x*, *ctcf24y* and *ctcf24x* in cobia. The red boxes indicate sex-specific insertions**.** (b) Amino acid sequence alignment of *cephx1y* and *cephx1x*, as well as *ctcf24y* and *ctcf24x* of cobia. (c) Structural model of Cobia *ephx1* (c1_Blue colour) and *tcf24* (c2_Green colour). The red color represents an extra helix-turn-helix domain in *cephx1*; the purple color indicates amino acid differences between the paralogs. N, N terminus; C, C terminus. (d) The expression profile of *ephx1* and *tcf24* in the testes and ovaries based on gonadal transcriptomic analysis. "*"and "**" indicate $p < 0.01$ and $p < 0.001$, respectively. (e) The interaction of *cephx1y* with CYP components (green and yellow shaded) and other proteins based on the STRING PPI network. Nodes represent genes, edges represent correlation between nodes. (f) Sex-specific markers in cobia. The PCR amplification in Panama, Brazil, Japan and Australia populations.

Sex specific DNA markers development and population specificity validation

To develop sex-specific markers, two forward (F) and two reverse (R) primers were designed to target the male-specific fragment insertion region within the sixth intron of *cephx1y*. The first set of primers, *cephx1*_1 (F1-R1), was designed to amplify a 404 bp region where there was an absolute deletion of the fragment for *cephx1x* in the region of FChr18, therefore the PCR would only amplify the "Y" sequence. The second set of primers, *cephx1*_2 (F2-R2), had the forward primer located in the male-specific region of *cephx1y*, while the reverse primer (R2) was situated in a region common to both *cephx1y* and *cephx1x*, resulting in a predicted PCR product size of 359 bp. Following the PCR validation of eight male and eleven female cobia individuals from Panama, both pairs of primers successfully amplified a male-specific product in all the unambiguously phenotypically sexed males (Fig. 4f).

Furthermore, distinct patterns were observed for the two markers during the analysis of cross-population validation. Similarly to Panama population, both *cephx1*_1 and *cephx1*_2 successfully amplified a PCR product from all males and was absent from females within the population of Brazil. Sanger sequencing of the PCR products, using both sets primers, confirmed the accuracy of the target sequences in both Panama and Brazilian populations. However, *cephx1*_1 did not show any amplification in both males and females in Japan and Australia populations, while *cephx1*_2 successfully amplified a shorter 180 bp product exclusively in males and females showed no amplification. In contrast, a longer 359 bp product was amplified from male DNA originating from the Brazilian and Panama populations (Fig. 4f). Subsequent Sanger sequencing of PCR products confirmed the presence of the 180 bp fragment in Japan and Australia populations. In addition, the failure of *cephx1_1* to amplify in Japan and Australia populations is due to its reverse primer being situated within the 179 bp (359 bp - 180 bp) missing region.

Discussion

The absence of a chromosome-level reference genome for cobia posed a significant challenge for in depth genomic analysis for the species, such as the investigation on its phylogenetic relationship with other teleosts and sex determination mechanisms. In the present study, we successfully obtained two high-quality genomes for both sexes of cobia using a combined strategy involving stLFR and Hi-C technologies. The high BUSCO score of 94.2% for the male and 93.8% for the female indicates the completeness of the two genome assemblies.

To our knowledge, this represents the first annotated chromosome-level reference genome of the species. These resources will provide researchers with opportunities to explore the molecular mechanisms controlling cobia's sex-determination system, and other economically important traits through genomic selection for faster growth, disease resistance and high-quality fillets. Moreover, it may also allow the development of further in-depth studies to better understand the biology of the species, such as how this large pelagic migrant inhabits all tropical and subtropical oceans of the globe and to inform more sustainable fisheries management practices.

The study of sex determination systems in teleosts can be technically challenging as most of them possess undifferentiated sex chromosomes and have various complex and diverse mechanisms for determining sex [34, 35]. Genome-wide association studies (GWAS) have gained popularity in exploring sex determination mechanisms, enabling the identification of sex-linked markers, sex determination loci and candidate genes [36-39]. In this study, we conducted GWAS to investigate the genetic basis of sex determination in cobia and identified a sex-associated region on MChr18, with 232 most significantly sex-linked SNPs that presented as heterozygote genotypes in all the males and complete homozygosity in all the females of captive Panama cobia population. The high density of sex-specific SNPs was a feature of the putative sex determination locus, which has also been observed in sex determination studies of other aquatic species [36, 40]. Moreover, this male-specific heterozygosity pattern suggested that the cobia has a male heterogametic sex determination system (XX/XY) that is consistent with being a gonochoristic species [1]. In addition, the selection signatures of Fixation index *Fst* and sequencing depth analysis served to further strengthen the evidence for the potential sex determination locus identified through GWAS. Taken together, the integration of GWAS, *Fst* scanning and sequencing coverage analysis identified a strongly sex-linked region and provided the evidence that the MChr18 is the potential undifferentiated homologue containing sex-specific loci, which is in agreement with the previous karyotype analysis where no morphologically distinct sex chromosomes for cobia were found [29]. The combination of these strategies has proven to be an effective approach for investigating the sex determination mechanism in cobia, as well as in other species [36, 41, 42]. Furthermore, it is noteworthy that the sex associated region on MChr18 identified preliminary in the male genome assembly by combining the stLFR and Hi-C reads contained

several gaps with variable length within the region and the flanking regions. The highly repeated content of this region complicated the assembly of the region. By further adding the PacBio HiFi reads, we obtained a small gap-free sex-linked region of 10.7 kb. This highlights the power of using long sequencing reads to assemble highly repetitive and complex genomic regions.

The small sex-associated region characterized on MChr18 contains two putative master sex-determining genes for cobia, *ephx1* (a short male-specific fragment insertion within Y1) and *tcf24* (between Y1 and Y2). Although *ephx1* has not previously been reported as a master sex gene or linked to sex determination, it has known to regulate endogenous steroid metabolism (i.e., androgens and estrogens), suggesting a functional role in sexual development and function in mammals [43]. A previous study in humans showed that upon treatment with an *ephx1* inhibitor, a decrease in estradiol formation was seen in ovaries [44]. In mice, *ephx1* is up-regulated in the embryo-containing oviduct and is thought to play a role in preimplantation embryo development [45]. However, its reproductive function in fish remains poorly studied [46-48]. Here, the *cepxh1y* was observed to be nearly exclusively expressed in the testes of adult fish by examining a recently published gonadal transcriptome of cobia [8]. In addition, a small male-specific insertion was detected in the sixth intron of *cephx1y,* which was in fact the identified male-specific region of Y1. Moreover, the loss of exon VII (a 41-amnio-acid helix-turn-helix domain) was only observed in the *cephx1Y* when compared to that of *cephx1x* and the other fish species and mammals. All these findings suggest that *cephx1y* could be a potential sex-determining gene in cobia. In addition, the PPI network analysis showed that *cephx1y* exhibits direct or indirect interactions with six Cyp genes and *hsd17b1.* The Cyp genes, specifically P450 aromatase (*cyp19a1a*), is known to have a crucial function in the development of gonads in various fish species [49, 50]. The *hsd17b1*, a gene involved in steroidogenic pathway, has been recognized as a master sex-determining gene in yellowtail species [26], which belong to the same order (*Carangiformes*) as cobia.

The *ephx1* gene encodes microsomal epoxide hydrolase (EPHX1), an enzyme known to be involved in the metabolism of xenobiotics and is thought to mediate functions including bioactivation and detoxification of environmental deleterious compounds [45, 51]. The occurrence of cobia intersex individuals has been reported in India [28] and Australia [3], with the latter observation (17% intersex) being attributed to increased levels of endocrine

disrupting compounds (EDC) from industrial and agricultural pollutants in local waterways. Interestingly, the EPHX1 enzyme has been reported to be involved in xenobiotic metabolism and regulates endogenous steroid metabolism [43]. Therefore, it stands to reason that EDC exposure could have an effect on EPHX1 catalytic activity, disrupting its functional associations with the cytochrome P450 family which mediate sex determination and differentiation pathways and potentially cause aberrations in gonadal development of cobia, more so given that *cephx1y* is the only male-specific gene detected in the species. Fish exhibit a wide variety of sex-determining genes [(for review see [17]], and more 'newcomers' with no previously known role in sex determination have also been discovered in recent years, such as *Paics* in blue tilapia (*Oreochromis aureus*) [52] and *bcar1* in channel catfish (*Ictalurus punctatus*) [27]. The present study has identified *cephx1y* as a novel potential sex-determination gene in cobia, offering new knowledge on the molecular mechanisms involved in teleost sex determination. Further functional experiments, such as genome-editing, are necessary to confirm and further explore these findings, as well as to clarify the complete mechanism by which *cephx1y* might modulate aromatase activity or other steps of the steroidogenic pathway in cobia and potentially other teleosts.

The transcription factor *tcf24* was first described in humans in 2002, but its functions remain largely unknown [53]. The only publication related to this factor in fish revealed that *tcf24* is upregulated in the hindbrain of individually-housed three-spine stickleback (*Gasterosteus aculeatus*) as a molecular basis for social behaviour [54]. The *ctcf24y* was significantly up-regulated in the testes by examining the data from the cobia gonadal transcriptome [8], suggesting a potential role in testicular differentiation of cobia. It's important to note that *tcf24* has a paralog, *tcf23* (also called OUT), which plays a role in mammalian reproduction. In humans, *tcf23* is a newly identified decidual mediator of progesterone action [55]. While in mice, it was expressed in adult reproductive tissues, e.g. uterus, ovaries and testes [56], indicating its potential role in male and female reproductive biology. Studies in fish have also shown that *tcf23* was highly upregulated in the ovaries of coho salmon (*Oncorhynchus kisutch*) after treatment with 11-KT (11-Ketotestosterone). In rainbow trout, *tcf23* was detected exclusively in the gonads of both sexes [57]. To date, functional studies of both *tcf24* and *tcf23* on reproductive biology are still very limited,

particularly in teleost fishes. Thus, further detailed functional characterization of *tcf24* is required to understand its potential role in sex determination and differentiation in cobia.

Identifying reliable and universally applicable sex-linked markers in fish poses challenges due to the considerable variability in sex-determination genes and systems, even among closely related species and within populations of the same species [58-60]. The current research successfully developed and validated two male-specific PCR-based markers (overlapping amplicons) targeting *cephx1* for cobia population of Panama. These two markers were validated and shown to be amplifiable only in males among individuals from Brazil. In the Japan and Australia populations, primers for *cephx1_1* did not amplify, while primers for *cephx1_2* amplified a shorter 180 bp products in males compared to the 359 bp found in the Brazil and Panama populations, revealing a shorter *cephx1y* intron 6 in the Asian and Australian populations when compared with the two populations from the Americas. Nevertheless, the absence of *cephx1y* in cobia females in far distant populations across the globe indicates a conserved role of *cephx1y* as a key putative sex-determining gene for the species. The development of this simple sex-specific PCR tool has the potential to significantly improve artificial fertilization and precise breeding in the cobia aquaculture industry, and ultimately leading to the development of monosex populations and increased productivity. Additionally, it aids non-lethal sampling and improves animal welfare in breeding programs.

The placement of the cobia (*R. canadum*) within the *Carangiformes* order and its status as the only member of the *Rachycentridae* family are well established. However, there have been divergent findings regarding its phylogenetic relationship to other species, particularly whether it is more closely related to the *Coryphaenidae* or *Echeneidae* [31-33, 61, 62]. In the current study, comparative genome analysis between *R. canadum* and one *Echeneidae* (*E. naucrates*) as well as two *Carangidaes* (*T. ovatus* and *S. lalandi*), showed that *R. canadum* and *E. naucrates* were sister groups, and the ancestor of *R. canadum* separated from the ancestor of *E. naucrates* approximately 51.4 million years ago. This investigation represents the first of its kind and provides insights into the evolutionary relationship of *R. canadum* through comparative genomic and phylogenetic analysis. Unfortunately, genomes of the only two species within *Coryphaenidae* family, the mahi-mahi or common dolphinfish (*C. hippurus*) and the pompano dolphinfish (*C. equiselis*), are not yet available. Therefore, a more complete

study of the evolution of the *Rachycentridae* genome (cobia as a single representative) needs to be further investigated when dolphinfish genomes become available.

Conclusions

We have successfully assembled and annotated high quality chromosome-level reference genomes for male and female cobia, which will provide a valuable resource for future investigations into the population structure, evolutionary history, fisheries management, and conservation of cobia and other *Carangiformes* species. Furthermore, we have discovered evidence of a male heterogametic (XX/XY) genetic sex determination system in cobia, with two genes *cephx1y* and *ctcf24y* as potential putative main drivers of cobia sex determination. Notably, *cephx1y* could represent a putative novel sex-determining gene, which further supports the rapid evolution of sex-determining mechanisms in teleost fish. Moreover, our development of a practical PCR-based method for identifying genetic sex in cobia can assist in the breeding monosex female populations in commercial farming of the species.

Materials and Methods

Experiential fish and sample collection

The majority of cobia individuals used in this study were obtained from Open Blue Sea Farms, the Republic of Panama. One male and one female adult fish at two-years-old were sampled for the whole genome *de novo* sequencing and assembly. In addition, a total of ninety-one fin clips from adult fish (thirty-one males and sixty females) were sampled for whole genome resequencing. For the development and validation of sex-specific DNA markers, the ovary and testis tissues were dissected from five male and five female fish, and nine fin clips (from the 91 referenced above) were chosen from three male and six female fish. Moreover, to validate the population specificity of the sex-specific DNA markers, fin clips from adult fish were obtained from three additional cultured populations in Japan (3 males and 5 females), Brazil (8 males and 5 females) and Australia (8 males and 10 females). Sex of fish individuals was determined through cannulation or gonadal observations.

Genome sequencing

High quality and molecular weight genomic DNA was extracted from fin clips of male and female cobia with QIAamp DNA purification kit (Qiagen) in accordance with the manufacturer's protocol. Paired-end single tube long fragment reads (stLFR) libraries [63] and Hi-C libraries were constructed using published protocols available via protocols.io

(http://dx.doi.org/10.17504/protocols.io.ss4eegw) [64], and sequenced on the BGISEQ-500 platform (BGI, Shenzhen, China) [65], yielding 100 bp paired-end (PE) reads. Barcodes were first split from stLFR raw reads and subsequently filtered by Soapfilter v2.2 (parameter: -y –p –M 2 –f -1 –Q 10) to generate high quality sequences. The genome sizes of the male and female cobia were estimated based on k-mer analysis (k=17) using Jellyfish v2.2.6 [66] and Genome Scope v1.0 [67]. Genome size was estimated with the formula genome size (Mb) = K-mer number/K-mer depth. For PacBio sequencing, high molecular weight gDNA from testis was extracted using a standard phenol/chloroform method. The testis was selected because it ensures certainty regarding the sex and it yielded high quality DNA. The integrity of the extracted DNA was assessed by 0.75% agarose gel electrophoresis, and the concentration was quantified by a Qubit 4 Fluorometer (Thermo Fisher Scientific, Inc., USA). Ten micrograms of DNA was then used to construct the library for PacBio SMRT Sequencing using the SMRTbell express template prep kit (PacBio, Menlo Park, CA, USA). The library was sequenced using the PacBio Sequel II System with CLR mode.

Genome assembly

The high quality paired-end single tube long fragment reads (stLFR), with read length of 100 bp, were used for initial genome assembly by employing the 10X Genomics software supernova [67]. First, the format of high quality reads was transformed to 10X Genomics format, and then the male and female cobia genomes were separately assembled with Supernova v2.1.1. To further improve the quality of the assembly, Gapcloser (v1.12) [68] was used with default parameters to fill gaps. Furthermore, Purge_haplotigs [69] was used to reduce redundancy of the initial assembly. The uniformity and completeness of the cobia male and female genome assemblies were evaluated by the read mapping rate as well as BUSCO (Benchmarking Universal Single-Copy Orthologs) [70]. Finally, chromosome level assemblies were constructed using Hi-C data. HiC-Pro v3.2 [71] was utilized to perform quality control of raw reads. Valid reads (the reads with contact information after processing of HiC-Pro pipeline, including read alignment, detection and filtering of valid interaction products, binning and contact map normalization) were used for assignment of contigs or scaffolds to chromosomes. Juicer v1.5 [72] and 3D-DNA (3D *de novo* assembly)[73] was used to anchor the male and female cobia genome assembly onto pseudo-chromosomes. In order to enhance the continuity of the sex-associated region, we further conducted genome assembly of a male

cobia using PacBio reads. The obtained HIFI long reads were fed to hifiasm (v0.14.1-r314) with the default parameters, and the primary assembly result p ctg.gfa file was converted into fasta format with in-house scripts.

Genome annotation

RepeatModeler v1.0.8 (http://repeatmasker.org/RepeatModeler/), LTR_FINDER v1.0.6 [74] and TRF tool v.4.09 [75] were used for *de novo* prediction of repeat elements based on the features of the repeat sequences. Homolog-based searches against the RepBase database (http://www.girinst.org/repbase (v21.01)) [76] using RepeatMasker v.3.3.0 and RepeatProteinMask v.3.3.0 were performed. Protein-coding genes were identified using a combination of homology-based and *de novo* prediction. For the homology-based gene prediction, homologous protein sequences of six well-annotated fish species including zebrafish, tongue sole, stickleback, tilapia, medaka and Japanese pufferfish were downloaded from Ensembl (release 94), while large yellow croaker was from NCBI. First, homologous proteins were aligned with the cobia genome using BLAT v319 [77], then GeneWise v2.4.1 [78] was employed to predict genes. For the *de novo* prediction, the *ab initio* gene prediction program of Augustus software v3.1 [79] was chosen, adopting zebrafish genes as a training dataset. Gene sets were integrated into a comprehensive and non-redundant gene set using GLEAN [80]. The completeness of final gene set was assessed by searching for 4,584 single-copy actinopterygian genes in BUSCO (Benchmarking Universal Single-Copy Orthologs). Non-coding RNAs (MicroRNA and rRNA) were also identified by aligning the cobia genome sequences to Rfam [81] using Infernal v1.1.1 [82]. While transfer RNAs (tRNAs) were defined using tRNAscan-SE v1.3.1 software with eukaryote default parameters. Functional annotation of the predicted protein-coding genes was conducted by aligning the predicted protein sequences to the public data base, including SwissProt, Interpro, TrEMBL, and KEGG databases using BLASTp with a maximal e-value of 1e-05.

Phylogenetic tree construction and divergence time estimation

To confirm the evolutionary status of cobia, nine other fish species including *Callorhinchus milii, E. naucrates, Seriola lalandi, Trachinotus ovatus, Epinephelus lanceolatus, Larimichthys crocea, Danio rerio, O. latipes* and *Gadus morhua* were selected to uncover orthologous gene sets and conduct genome phylogenetic analysis. The male cobia genome was chosen as the representative of *R. canadum* to define gene families. For the other nine teleosts, protein

sequences of *C. milii, E. naucrates, S. lalandi, L. crocea, D. rerio, O. latipes* and *G. morhua* were downloaded from Ensembl (release 99), the *E. lanceolatu* from NCBI (GCF_005281545.1), and *T. ovatus* from figshare (https://figshare.com/articles/dataset/Whole_genome_sequencing_of_female_pompano_Trachinotus_ovatus_/7570727). All-to-all orthologous genes were aligned using BLASTP v2.2.26 with e-value cutoff of 1e-7. Gene families were clustered by TreeFam [83] pipeline. For phylogenetic tree analysis, single-copy gene families from male cobia and nine other fish species were aligned using MUSCLE v3.8.31 [84]. Phase 1 sites were extracted and merged to a supergene as an input of MrBayes v3.1.2 [85] with *C. milii* as the outgroup. The divergence time for cobia and the other three Carangiformes (*T. ovatus*, S. *lalandi* and *E. naucrates*) was estimated by MCMCTree from the PAML v4.4 [86], package based on the HKY85 model. Correlated rates were used for a molecular clock model. Three calibration fossil evidence was found using the website TimeTree (http://www.timetree.org), including *C. milii* with other teleost fish (453-497 Mya), *O. latipes* with perciformes species (104-145 Mya) and *E. naucrates* with *S. lalandi* (70-86 Mya).

Whole-genome resequencing and identification of the sexual specific genomic region

Genomic DNA was isolated from the fin clips of individual fish (thirty-one males and sixty females) and used to construct 100 bp PE libraries, and sequenced with Dipseq-T1 platform. Raw reads that either contained more than 10% Ns, contained adaptors or had a half base quality below 12 were discarded. Filtered reads (2,680 Gbp in total) were then mapped to the male reference genome, which resulted in an average mapping rate of 99.04% and 49.42X depth. The population SNPs were called with Accelerated Sentieon node [87], and sites were filtered which matched the condition “QD < 2.0 || MQ < 40.0 || MQRankSum < -12.5 || ReadPosRankSum < -8.0 || FS>60.0 || SOR>3.0”. Finally, a merged vcf for ninety-one samples with filtered SNPs (filtering with --max-missing 0.8 --maf 0.05 --minDP 4 --min-meanDP 3) on chromosomes were generated and used for later comparative analysis. Filtered SNPs were annotated by SnpEff (v 4.3t) [88], and then classified into regions of exon, intron, splicing site, and upstream and downstream intergenic regions.

Using the male genome as reference, we employed two different strategies to identify the sex-specific region(s) in cobia. A genome-wide association study (GWAS) was first performed using EMMAX (http://genetics.cs.ucla.edu/emmax/index.html) [89], mixed linear model, to

test whether any of the SNPs identified were significantly associated with sex. The first 10 PCs of PCA from plink (v1.90b6.12) were used as concomitant variables at the same time. Secondly, we calculated depth of each site for all ninety-one samples using samtools-depth module (v-1.9, http://www.htslib.org/). The average depth distribution analysis between the male and female group (bin 50 bp, normalized per sequencing depth of each sample) was also employed by exploiting the difference in sex chromosome ploidy between males and females. The *Fst* (Wright's fixation index) between male and female groups and θπ (nucleotide diversity) of each group were calculated by vcftools (v0.1.13**,** http://vcftools.sourceforge.net/). A variant density approach was performed by searching for differences in SNP density between males and females. The protein-protein interaction (PPI) network prediction (http://cn.string-db.org) was adopted for the identified putative master sex-determining gene for cobia.

Development of sex-specific markers and population specificity validation

Sex-specific primers were designed using Primer3 [90] in Geneious Prime 2021.2.2 (Biomatters). Two sets of primers (*cephx1_1*) (Forward: 5′-ATCCAACATTTCAAGATCAACAGGTT-3′; Reverse: 5′-GGGGACATCCTGATATCTAACCAATA-3′) (*cephx1_2*) (Forward: 5′-GCTAGTTTAGAAAATGACAGCTCACA-3′; Reverse: 5′-GTAAAATTCCAAGATGTGAACAAGCC-3′) for c*ephx1* were designed based on a 540 bp continuous fragment insertion in males where there is an absolute deletion for the gene in females. PCR conditions were first tested on two individual samples (one male and one female) to verify PCR amplification and presence (in males)/absence (in females) polymorphism, then further screened on more sexed fish from Panama, Brazil, Japan, and Australia. For the Panama population, PCR was carried out using Taq PCR Core KitTM (QIAGEN), in 20 μL reaction that contained 2.08 μL 10X Taq Buffer, 0.42 μl of dNTPs (10 μM), 0.67 μL of each primer (10 μM), 0.17 μL of Taq DNA polymerase (5 units/μL), 30 ng of extracted DNA, and made up to final volume with nuclease-free water. The PCR cycling conditions included an initial incubation for 3 min at 94 °C followed by 30 cycles that included denaturation for 1 min at 94 °C, annealing for 1 min at 60°C (c*ephx1_1*) and 61°C (c*ephx1_2*), and extension for 1 min at 72°C. Samples were incubated for 10 min at 72°C for a final extension. For the Brazil population, PCR was performed under conditions similar to those in Panama, with the exception of using 35 ng as the template, an annealing temperature of 66

°C for 30 cycles for *cephx1_1*, and 20 ng, 25 cycles with an annealing temperature of 65°C for *cephx1_2*. For the Australia population, PCR was carried out using PlatinumTM Taq DNA Polymerase High Fidelity (Invitrogen), in 20 µl reaction mixtures that contained 2 µL 10X Buffer, 0.4 µL of dNTPs (10 µM), 0.8 µl of $MgSO_4$, 0.4 µL of each primer (10 µM), 0.08 µL of Taq DNA polymerase (5 units/µL) and 32 ng of DNA. The PCR cycling conditions included an initial incubation for 2 min at 94 °C followed by 30 cycles that included denaturation for 15 sec at 94 °C, annealing for 30 sec at 61 °C (*cephx1_1*) and 62°C (*cephx1_2*), and extension for 1 min at 72°C. Samples were incubated for 10 min at 72°C for a final extension. For the Japan population, PCR was carried out using Q5 High-Fidelity 2X master Mix, in a 25 µL reaction that contained 12.5 µL Q5, 1 µL of each primer (10 µM), 15 ng of extracted DNA, and made up to final volume with nuclease-free water. The cycling conditions included an initial incubation for 30 s at 98°C followed by 30 cycles for both set of primers that included denaturation for 10 s at 98°C, annealing for 30 s at 61°C, and extension for 30 s at 72°C. Samples were incubated for 2 min at 72°C for a final extension. PCR products were analysed in 2% agarose using gel electrophoresis, and then several selected products were purified and Sanger sequenced.

Abbreviations

stLFR: single tube long fragment reads; BUSCO: Benchmarking Universal Single-Copy Orthologs; Gb: gigabase; GWAS: genome-wide association study; SNP: single-nucleotide polymorphism

Authors' Contributions

X. S.: project coordination and data analysis, wrote the first draft of the manuscript. J.D.: conceived and managed the project, obtained funding and revised the manuscript. J.Y.: conceived the project, obtained funding and revised the manuscript. G.BG.: sample logistics, DNA extractions and quality controls, revised the manuscript. Z.P.: developed and validated sex-specific PCR assays and revised the manuscript. J.H.: genome assembly of Pacbio data, GWAS and sequencing depth analysis and writing. L.S.: population genomics and GWAS. X.G.: genome assembly of stLFR and Hi-C reads and genome annotation. Y.S.: sequencing and project coordination. G.F.: conceived the project and revised the manuscript. D.F. and J.A.: carried out the growth experiments and sampling in Panama. R.H. and C.L.: Validation of sex-specific DNA markers through sample collection and cross-population analysis in Japan. E.G.

and K.A.: validation of sex-specific DNA markers through sample collection and cross-population analysis in Australia. All authors have contributed to and revised the final version.

Funding

This work was funded by the Cobia Genome Consortium established between Open Blue Sea Farms Panama S.A. Cobia (OBSF), James Cook University Singapore (JCUS) and University of Chile (UoC).

Data Availability

The raw read data used to generate the genome assembly, and the whole genome resequencing data have been deposited in the NCBI BioProject database under accession code PRJNA864890 (https://dataview.ncbi.nlm.nih.gov/object/PRJNA864890?reviewer=p71gunhibl9uu8l8i9bc44m6nr). The final assembly data of the male and female cobia genome have been submitted to NCBI (data to be released at the time of acceptance). Hence, we generate a private/personal link for both the assembly files and annotation files for editors and reviewers (https://pan.genomics.cn/ucdisk/s/36Fjqi).

Competing Interests The authors declare no competing interests.

Ethics Declarations

All fish samples and data reported in this study were historical datasets and preserved samples of harvested (dead) fish from commercial production destined for human consumption. The samples used in this study were not derived from experimentally manipulated animals. Research on harvested (dead) commercially farmed animals do not require approval from James Cook University's Institutional Animal Care and Use Committee (IACUC).

Acknowledgements

The authors are grateful to Hiroyuki Nakamura at the Okinawa Prefectural Sea Farming Center, who provided us with the samples of cobia from Japan. We are also grateful to Kazuo Tonack (Maricultura Costa Verde, Angra dos Reis, Brazil), Klaudia Kerber and Pedro Kerber (Redemar Alevinos, Ilhabela, Brazil), Angelica Bastos Leite, Natalia Domingos Bento, Mariana Santos Domingos Barletta and Antonio Ricardo da Silva Domingos for the samples obtained from Brazil.

References

1. Schaffer RV and Nakamura EL. Synopsis of biological data on the cobia Rachycentron canadum (Pisces: Rachycentridae). 1989.
2. Benetti DD, Suarez J, Camperio J, Hoenig RH, Tudela CE, Daugherty Z, et al. A review on cobia, Rachycentron canadum, aquaculture. Journal of the World Aquaculture Society. 2021.
3. Dutney L, Elizur A and Lee P. Analysis of sexually dimorphic growth in captive reared cobia (Rachycentron canadum) and the occurrence of intersex individuals. Aquaculture. 2017;468:348-55.
4. Liao I-C, Leaño E, Hsu C and Ku C. Marine cage culture of cobia in Taiwan. 2007.
5. Sampaio LA, Moreira CB, Miranda‐Filho KC and Rombenso AN. Culture of cobia Rachycentron canadum (L) in near‐shore cages off the Brazilian coast. Aquaculture Research. 2011;42 6:832-4.
6. Molina WF, Benetti DD, Fiorentino JN, de Lima-Filho PA, Alencar CERD, da Costa GWWF, et al. Early sex shape dimorphism (SShD) in Rachycentron canadum (Linnaeus, 1766) and its applications for monosex culture. Aquaculture. 2018;495:320-7.
7. Díaz-Muñoz DN, Díaz N, Torres O, Leiva JC, Palacios H, Romero F, et al. Culture of cobia Rachycentron canadum in a recirculation aquaculture system in northern Chile. Latin american journal of aquatic research. 2019;47 5:733-42.
8. Shen X, Yáñez JM, Bastos Gomes G, Poon ZWJ, Foster D, Alarcon JF, et al. Comparative gonad transcriptome analysis in cobia (Rachycentron canadum). Frontiers in Genetics. 2023;14:1128943.
9. Figueras A, Costa M and Novoa B. Applications of functional genomics in molluscs aquaculture. Functional genomics in aquaculture. 2012:377.
10. Mank JE, Promislow DE and Avise JC. Evolution of alternative sex-determining mechanisms in teleost fishes. Biological Journal of the Linnean Society. 2006;87 1:83-93.
11. Oliveira C and Toledo LFdA. Evidence of an XX/XY sex chromosome system in the fish Dormitator maculatus (Teleostei, Eleotrididae). Genetics and Molecular Biology. 2006;29 4:653-5.
12. Chen S, Zhang G, Shao C, Huang Q, Liu G, Zhang P, et al. Whole-genome sequence of a flatfish provides insights into ZW sex chromosome evolution and adaptation to a benthic lifestyle. Nature genetics. 2014;46 3:253-60.
13. Kelley JL, Yee M-C, Brown AP, Richardson RR, Tatarenkov A, Lee CC, et al. The genome of the self-fertilizing mangrove rivulus fish, Kryptolebias marmoratus: a model for studying phenotypic plasticity and adaptations to extreme environments. Genome biology and evolution. 2016;8 7:2145-54.
14. Piferrer F. Epigenetics of sex determination and differentiation in fish. Sex control in aquaculture. 2018:65-83.
15. Penman DJ and Piferrer F. Fish gonadogenesis. Part I: genetic and environmental mechanisms of sex determination. Reviews in Fisheries Science. 2008;16 sup1:16-34.
16. Domingos JA, Budd AM, Banh QQ, Goldsbury JA, Zenger KR and Jerry DR. Sex-specific dmrt1 and cyp19a1 methylation and alternative splicing in gonads of the protandrous hermaphrodite barramundi. PLoS One. 2018;13 9:e0204182.
17. Chen J, Zhu Z and Hu W. Progress in research on fish sex determining genes. Water Biology and Security. 2022;1 1:100008.
18. Yano A, Guyomard R, Nicol B, Jouanno E, Quillet E, Klopp C, et al. An immune-related gene evolved into the master sex-determining gene in rainbow trout, Oncorhynchus mykiss. Current Biology. 2012;22 15:1423-8.
19. Nanda I, Kondo M, Hornung U, Asakawa S, Winkler C, Shimizu A, et al. A duplicated copy of DMRT1 in the sex-determining region of the Y chromosome of the medaka, Oryzias latipes. Proceedings of the National Academy of Sciences. 2002;99 18:11778-83.

20. Matsuda M, Nagahama Y, Shinomiya A, Sato T, Matsuda C, Kobayashi T, et al. DMY is a Y-specific DM-domain gene required for male development in the medaka fish. Nature. 2002;417 6888:559-63.
21. Hattori RS, Murai Y, Oura M, Masuda S, Majhi SK, Sakamoto T, et al. A Y-linked anti-Müllerian hormone duplication takes over a critical role in sex determination. Proceedings of the National Academy of Sciences. 2012;109 8:2955-9.
22. Li M, Sun Y, Zhao J, Shi H, Zeng S, Ye K, et al. A tandem duplicate of anti-Müllerian hormone with a missense SNP on the Y chromosome is essential for male sex determination in Nile tilapia, Oreochromis niloticus. PLoS genetics. 2015;11 11:e1005678.
23. Peichel CL, McCann SR, Ross JA, Naftaly AF, Urton JR, Cech JN, et al. Assembly of the threespine stickleback Y chromosome reveals convergent signatures of sex chromosome evolution. Genome Biology. 2020;21 1:1-31.
24. Jeffries DL, Mee JA and Peichel CL. Identification of a candidate sex determination gene in Culaea inconstans suggests convergent recruitment of an Amh duplicate in two lineages of stickleback. Journal of evolutionary biology. 2022;35 12:1683-95.
25. Pan Q, Feron R, Yano A, Guyomard R, Jouanno E, Vigouroux E, et al. Identification of the master sex determining gene in Northern pike (Esox lucius) reveals restricted sex chromosome differentiation. PLoS genetics. 2019;15 8:e1008013.
26. Koyama T, Nakamoto M, Morishima K, Yamashita R, Yamashita T, Sasaki K, et al. A SNP in a steroidogenic enzyme is associated with phenotypic sex in Seriola fishes. Current Biology. 2019;29 11:1901-9. e8.
27. Bao L, Tian C, Liu S, Zhang Y, Elaswad A, Yuan Z, et al. The Y chromosome sequence of the channel catfish suggests novel sex determination mechanisms in teleost fish. BMC biology. 2019;17 1:1-16.
28. Sakthivel M, Tamilmani G, Jayakumar R, AK AN, Sankar M, Anikuttan K, et al. First report of intersex in Cobia, Rachycentron canadum reared in Gulf of Mannar, India—A case study. Aquaculture Reports. 2021;19:100587.
29. Jacobina UP, Cioffi MdB, Souza LGR, Calado LL, Tavares M, Manzella J, et al. Chromosome mapping of repetitive sequences in Rachycentron canadum (Perciformes: Rachycentridae): implications for karyotypic evolution and perspectives for biotechnological uses. Journal of Biomedicine and Biotechnology. 2011;2011.
30. Nelson JS, Grande TC and Wilson MV. Fishes of the World. John Wiley & Sons; 2016.
31. Johnson GD. Percoidei: development and relationships. Ontogeny and systematics of fishes. 1984.
32. O'Toole B. Phylogeny of the species of the superfamily Echeneoidea (Perciformes: Carangoidei: Echeneidae, Rachycentridae, and Coryphaenidae), with an interpretation of echeneid hitchhiking behaviour. Canadian Journal of Zoology. 2002;80 4:596-623.
33. Wang Z, Guo Y, Liu C and Liu Y. The complete mitochondrial DNA of cobia (Rachycentron canadum) and phylogenetics of carangoid. Acta Hydrobiologica Sinica. 2011;35 2:229-37.
34. Rajendiran P, Jaafar F, Kar S, Sudhakumari C, Senthilkumaran B and Parhar IS. Sex determination and differentiation in teleost: roles of genetics, environment, and Brain. Biology. 2021;10 10:973.
35. Kuhl H, Guiguen Y, Höhne C, Kreuz E, Du K, Klopp C, et al. A 180 My-old female-specific genome region in sturgeon reveals the oldest known vertebrate sex determining system with undifferentiated sex chromosomes. bioRxiv. 2020:2020.10. 10.334367.
36. Wang Y, Yang Y, Li Y and Chen M. Identification of sex determination locus in sea cucumber Apostichopus japonicus using genome-wide association study. BMC genomics. 2022;23 1:391.
37. Petit J, Salentijn EM, Paulo M-J, Denneboom C and Trindade LM. Genetic architecture of flowering time and sex determination in hemp (Cannabis sativa L.): a genome-wide association study. Frontiers in plant science. 2020;11:569958.

38. Wang Q, Liu Y, Wang Y, Jiang S, Zhang C and Li B. GWAS Reveal Novel Sex-Related Markers and Candidate Genes in Sea Urchin Mesocentrotus nudus. Marine Biotechnology. 2021:1-8.
39. Luo H, Xiao J, Jiang Y, Ke Y, Ke C and Cai M. Mapping and marker identification for sex-determining in the Pacific abalone, Haliotis discus hannai Ino. Aquaculture. 2021;530:735810.
40. Li YL, Xing TF and Liu JX. Genome‐wide association analyses based on whole‐genome sequencing of Protosalanx hyalocranius provide insights into sex determination of Salangid fishes. Molecular ecology resources. 2020;20 4:1038-49.
41. Lin H, Zhou Z, Zhao J, Zhou T, Bai H, Ke Q, et al. Genome-wide association study identifies genomic loci of sex determination and gonadosomatic index traits in large yellow croaker (Larimichthys crocea). Marine Biotechnology. 2021;23:127-39.
42. He L, Jia KH, Zhang RG, Wang Y, Shi TL, Li ZC, et al. Chromosome‐scale assembly of the genome of Salix dunnii reveals a male‐heterogametic sex determination system on chromosome 7. Molecular Ecology Resources. 2021;21 6:1966-82.
43. Fändrich F, Degiuli B, Vogel-Bindel U, Arand M and Oesch F. Induction of rat liver microsomal epoxide hydrolase by its endogenous substrate 16α, 17α-epoxyestra-1, 3, 5-trien-3-ol. Xenobiotica. 1995;25 3:239-44.
44. Hattori N, Fujiwara H, Maeda M, Fujii S and Ueda M. Epoxide hydrolase affects estrogen production in the human ovary. Endocrinology. 2000;141 9:3353-65.
45. Cheong AW, Lee Y-L, Liu W-M, Yeung WS and Lee K-F. Oviductal microsomal epoxide hydrolase (EPHX1) reduces reactive oxygen species (ROS) level and enhances preimplantation mouse embryo development. Biology of reproduction. 2009;81 1:126-32.
46. Blay C, Haffray P, Bugeon J, D'ambrosio J, Dechamp N, Collewet G, et al. Genetic parameters and genome-wide association studies of quality traits characterised using imaging technologies in Rainbow trout, Oncorhynchus mykiss. Frontiers in Genetics. 2021;12:219.
47. Yan Y-L, Batzel P, Titus T, Sydes J, Desvignes T, BreMiller R, et al. A hormone that lost its receptor: anti-Müllerian hormone (AMH) in zebrafish gonad development and sex determination. Genetics. 2019;213 2:529-53.
48. Firmino JP, Vallejos-Vidal E, Sarasquete C, Ortiz-Delgado JB, Balasch JC, Tort L, et al. Unveiling the effect of dietary essential oils supplementation in Sparus aurata gills and its efficiency against the infestation by Sparicotyle chrysophrii. Scientific reports. 2020;10 1:17764.
49. Bertho S, Herpin A, Branthonne A, Jouanno E, Yano A, Nicol B, et al. The unusual rainbow trout sex determination gene hijacked the canonical vertebrate gonadal differentiation pathway. Proceedings of the National Academy of Sciences. 2018;115 50:12781-6.
50. Guiguen Y, Fostier A, Piferrer F and Chang C-F. Ovarian aromatase and estrogens: a pivotal role for gonadal sex differentiation and sex change in fish. General and comparative endocrinology. 2010;165 3:352-66.
51. Gautheron J and Jéru I. The multifaceted role of epoxide hydrolases in human health and disease. International Journal of Molecular Sciences. 2020;22 1:13.
52. Tao W, Xu L, Zhao L, Zhu Z, Wu X, Min Q, et al. High‐quality chromosome‐level genomes of two tilapia species reveal their evolution of repeat sequences and sex chromosomes. Molecular ecology resources. 2021;21 2:543-60.
53. Dolmatov IY. Molecular Aspects of Regeneration Mechanisms in Holothurians. Genes. 2021;12 2:250.
54. Greenwood AK and Peichel CL. Social regulation of gene expression in threespine sticklebacks. PLoS One. 2015;10 9:e0137726.
55. Kommagani R, Szwarc MM, Kovanci E, Creighton CJ, O'Malley BW, DeMayo FJ, et al. A murine uterine transcriptome, responsive to steroid receptor coactivator-2, reveals transcription factor 23 as essential for decidualization of human endometrial stromal cells. Biology of reproduction. 2014;90 4:75, 1-11.

56. Narumi O, Mori S, Boku S, Tsuji Y, Hashimoto N, Nishikawa S-I, et al. OUT, a novel basic helix-loop-helix transcription factor with an Id-like inhibitory activity. Journal of Biological Chemistry. 2000;275 5:3510-21.
57. Antoine R, Jean-Jacques L, Anne-Sophie G, Jé M, Marie-Jo R, Karine H, et al. Expression profiling of rainbow trout testis development identifies evolutionary conserved genes involved in spermatogenesis. 2009.
58. Pavlova A, Harrisson KA, Turakulov R, Lee YP, Ingram BA, Gilligan D, et al. Labile sex chromosomes in the Australian freshwater fish family Percichthyidae. Molecular Ecology Resources. 2022;22 4:1639-55.
59. Faggion S, Vandeputte M, Chatain B, Gagnaire P-A and Allal F. Population-specific variations of the genetic architecture of sex determination in wild European sea bass Dicentrarchus labrax L. Heredity. 2019;122 5:612-21.
60. Ferraresso S, Bargelloni L, Babbucci M, Cannas R, Follesa MC, Carugati L, et al. fshr: a fish sex-determining locus shows variable incomplete penetrance across flathead grey mullet populations. Iscience. 2021;24 1.
61. Near TJ, Eytan RI, Dornburg A, Kuhn KL, Moore JA, Davis MP, et al. Resolution of ray-finned fish phylogeny and timing of diversification. Proceedings of the National Academy of Sciences. 2012;109 34:13698-703.
62. Gray KN, McDowell JR, Collette BB and Graves JE. A molecular phylogeny of the remoras and their relatives. Bulletin of Marine Science. 2009;84 2:183-97.
63. Wang O, Chin R, Cheng X, Wu MKY, Mao Q, Tang J, et al. Efficient and unique cobarcoding of second-generation sequencing reads from long DNA molecules enabling cost-effective and accurate sequencing, haplotyping, and de novo assembly. Genome research. 2019;29 5:798-808.
64. Belton J-M, McCord RP, Gibcus JH, Naumova N, Zhan Y and Dekker J. Hi–C: a comprehensive technique to capture the conformation of genomes. Methods. 2012;58 3:268-76.
65. Huang J, Liang X, Xuan Y, Geng C, Li Y, Lu H, et al. BGISEQ-500 WGS library construction. protocols io. 2018:1-10.
66. Marçais G and Kingsford C. A fast, lock-free approach for efficient parallel counting of occurrences of k-mers. Bioinformatics. 2011;27 6:764-70.
67. Zheng GX, Lau BT, Schnall-Levin M, Jarosz M, Bell JM, Hindson CM, et al. Haplotyping germline and cancer genomes with high-throughput linked-read sequencing. Nature biotechnology. 2016;34 3:303-11.
68. Luo R, Liu B, Xie Y, Li Z, Huang W, Yuan J, et al. SOAPdenovo2: an empirically improved memory-efficient short-read de novo assembler. Gigascience. 2012;1 1:2047-217X-1-18.
69. Roach MJ, Schmidt SA and Borneman AR. Purge Haplotigs: allelic contig reassignment for third-gen diploid genome assemblies. BMC bioinformatics. 2018;19 1:1-10.
70. Simão FA, Waterhouse RM, Ioannidis P, Kriventseva EV and Zdobnov EM. BUSCO: assessing genome assembly and annotation completeness with single-copy orthologs. Bioinformatics. 2015;31 19:3210-2.
71. Servant N, Varoquaux N, Lajoie BR, Viara E, Chen C-J, Vert J-P, et al. HiC-Pro: an optimized and flexible pipeline for Hi-C data processing. Genome biology. 2015;16 1:1-11.
72. Durand NC, Shamim MS, Machol I, Rao SS, Huntley MH, Lander ES, et al. Juicer provides a one-click system for analyzing loop-resolution Hi-C experiments. Cell systems. 2016;3 1:95-8.
73. Dudchenko O, Batra SS, Omer AD, Nyquist SK, Hoeger M, Durand NC, et al. De novo assembly of the Aedes aegypti genome using Hi-C yields chromosome-length scaffolds. Science. 2017;356 6333:92-5.
74. Xu Z and Wang H. LTR_FINDER: an efficient tool for the prediction of full-length LTR retrotransposons. Nucleic acids research. 2007;35 suppl_2:W265-W8.
75. Benson G. Tandem repeats finder: a program to analyze DNA sequences. Nucleic acids research. 1999;27 2:573-80.

76. Bao W, Kojima KK and Kohany O. Repbase Update, a database of repetitive elements in eukaryotic genomes. Mobile Dna. 2015;6 1:1-6.
77. Kent WJ. BLAT—the BLAST-like alignment tool. Genome research. 2002;12 4:656-64.
78. Birney E, Clamp M and Durbin R. GeneWise and genomewise. Genome research. 2004;14 5:988-95.
79. Stanke M, Diekhans M, Baertsch R and Haussler D. Using native and syntenically mapped cDNA alignments to improve de novo gene finding. Bioinformatics. 2008;24 5:637-44.
80. Elsik CG, Mackey AJ, Reese JT, Milshina NV, Roos DS and Weinstock GM. Creating a honey bee consensus gene set. Genome biology. 2007;8 1:1-8.
81. Kalvari I, Argasinska J, Quinones-Olvera N, Nawrocki EP, Rivas E, Eddy SR, et al. Rfam 13.0: shifting to a genome-centric resource for non-coding RNA families. Nucleic acids research. 2018;46 D1:D335-D42.
82. Nawrocki EP and Eddy SR. Infernal 1.1: 100-fold faster RNA homology searches. Bioinformatics. 2013;29 22:2933-5.
83. Li H, Coghlan A, Ruan J, Coin LJ, Heriche J-K, Osmotherly L, et al. TreeFam: a curated database of phylogenetic trees of animal gene families. Nucleic acids research. 2006;34 suppl_1:D572-D80.
84. Edgar RC. MUSCLE: multiple sequence alignment with high accuracy and high throughput. Nucleic acids research. 2004;32 5:1792-7.
85. Huelsenbeck JP and Ronquist F. MRBAYES: Bayesian inference of phylogenetic trees. Bioinformatics. 2001;17 8:754-5.
86. Yang Z. PAML 4: phylogenetic analysis by maximum likelihood. Molecular biology and evolution. 2007;24 8:1586-91.
87. Freed D, Aldana R, Weber JA and Edwards JS. The Sentieon Genomics Tools-A fast and accurate solution to variant calling from next-generation sequence data. BioRxiv. 2017:115717.
88. Cingolani P, Platts A, Wang LL, Coon M, Nguyen T, Wang L, et al. A program for annotating and predicting the effects of single nucleotide polymorphisms, SnpEff: SNPs in the genome of Drosophila melanogaster strain w1118; iso-2; iso-3. Fly. 2012;6 2:80-92.
89. Kang HM, Sul JH, Service SK, Zaitlen NA, Kong S-y, Freimer NB, et al. Variance component model to account for sample structure in genome-wide association studies. Nature genetics. 2010;42 4:348-54.
90. Rozen S and Skaletsky H. Primer3 on the WWW for general users and for biologist programmers. Bioinformatics methods and protocols. Springer; 2000. p. 365-86.

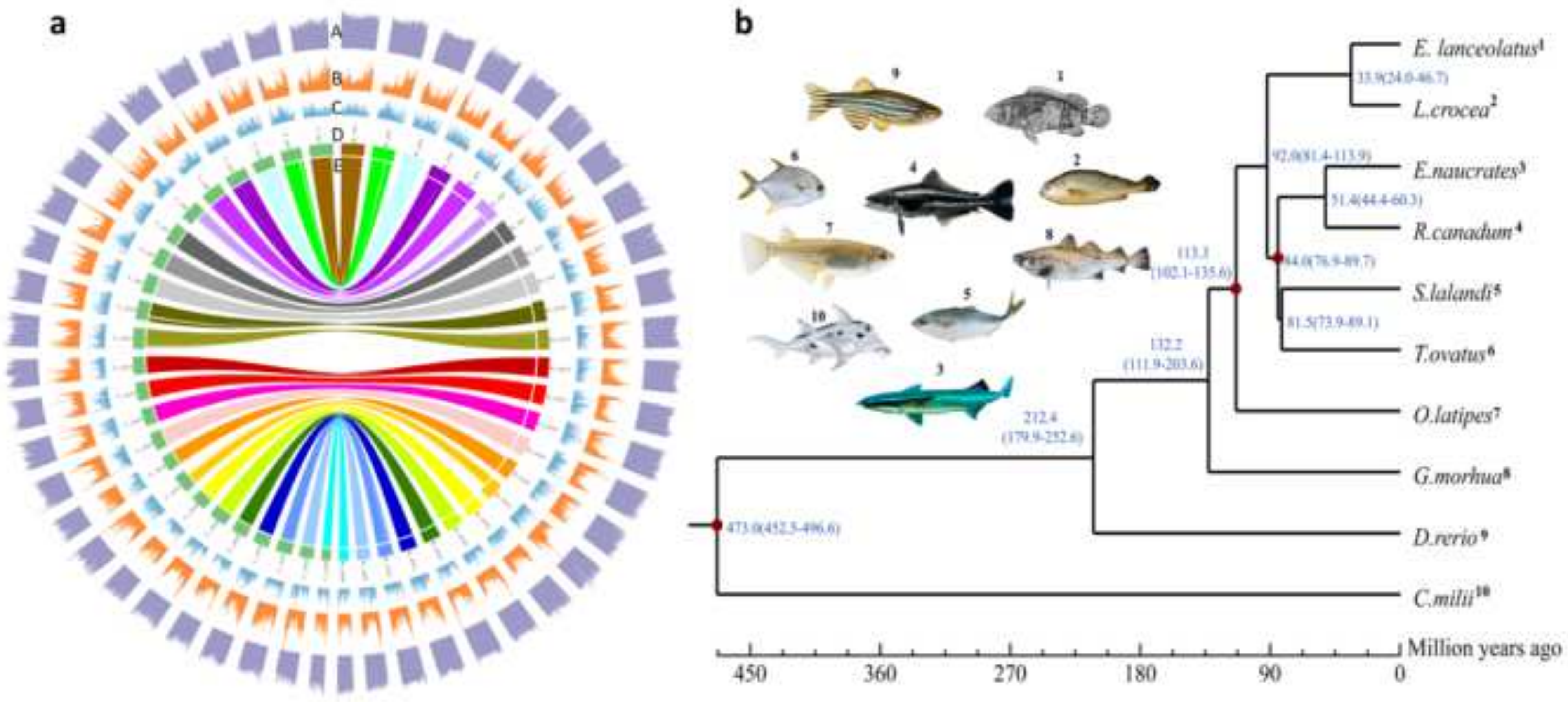
a
A
B
C
D
E
b
E. lanceolatus1
L.crocea2
E.naucrates3
R.canadum4
S.lalandi5
T.ovatus6
O.latipes7
G.morhua8
D.rerio9
C.milii10
113.3 (102.1-135.6)
132.2 (111.9-203.6)
212.4 (179.9-252.6)
51.4(44.4-60.3)
81.5(73.9-89.1)
Million years ago
450
360
270
180
90
0

Figure
Click here to access/download;Figure;Figure 2.TIF

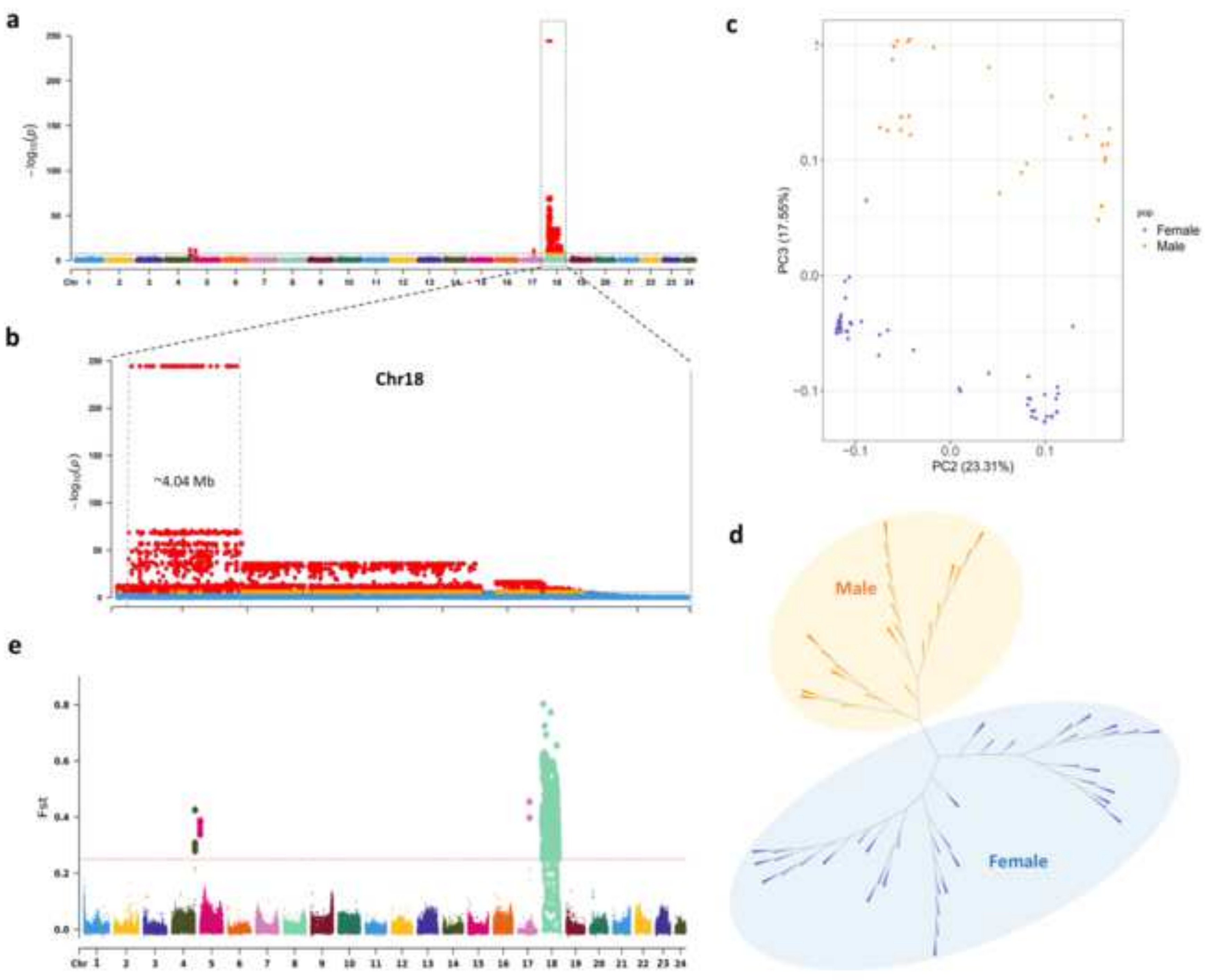

Figure

Click here to access/download;Figure;Figure 3.TIF

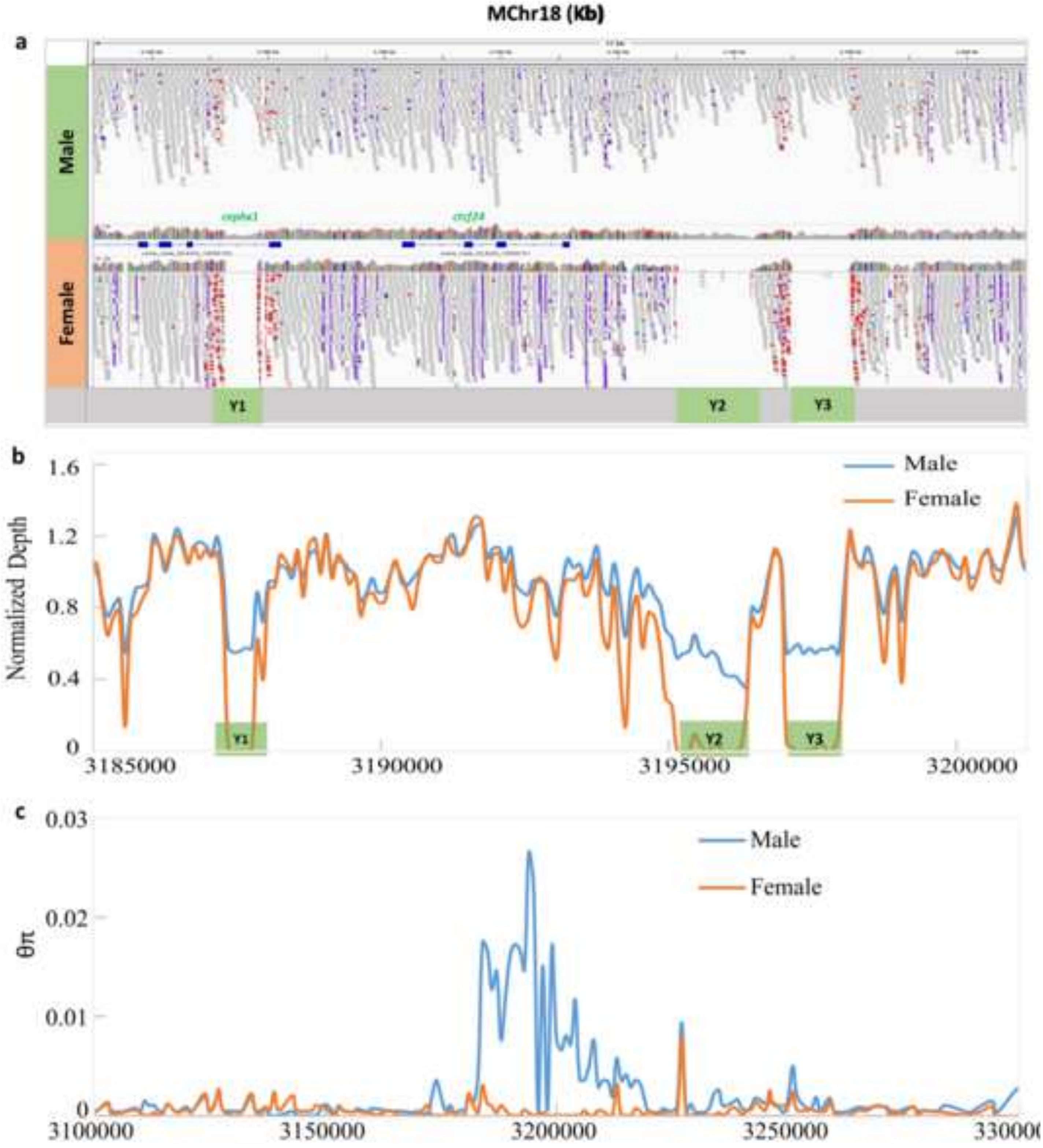

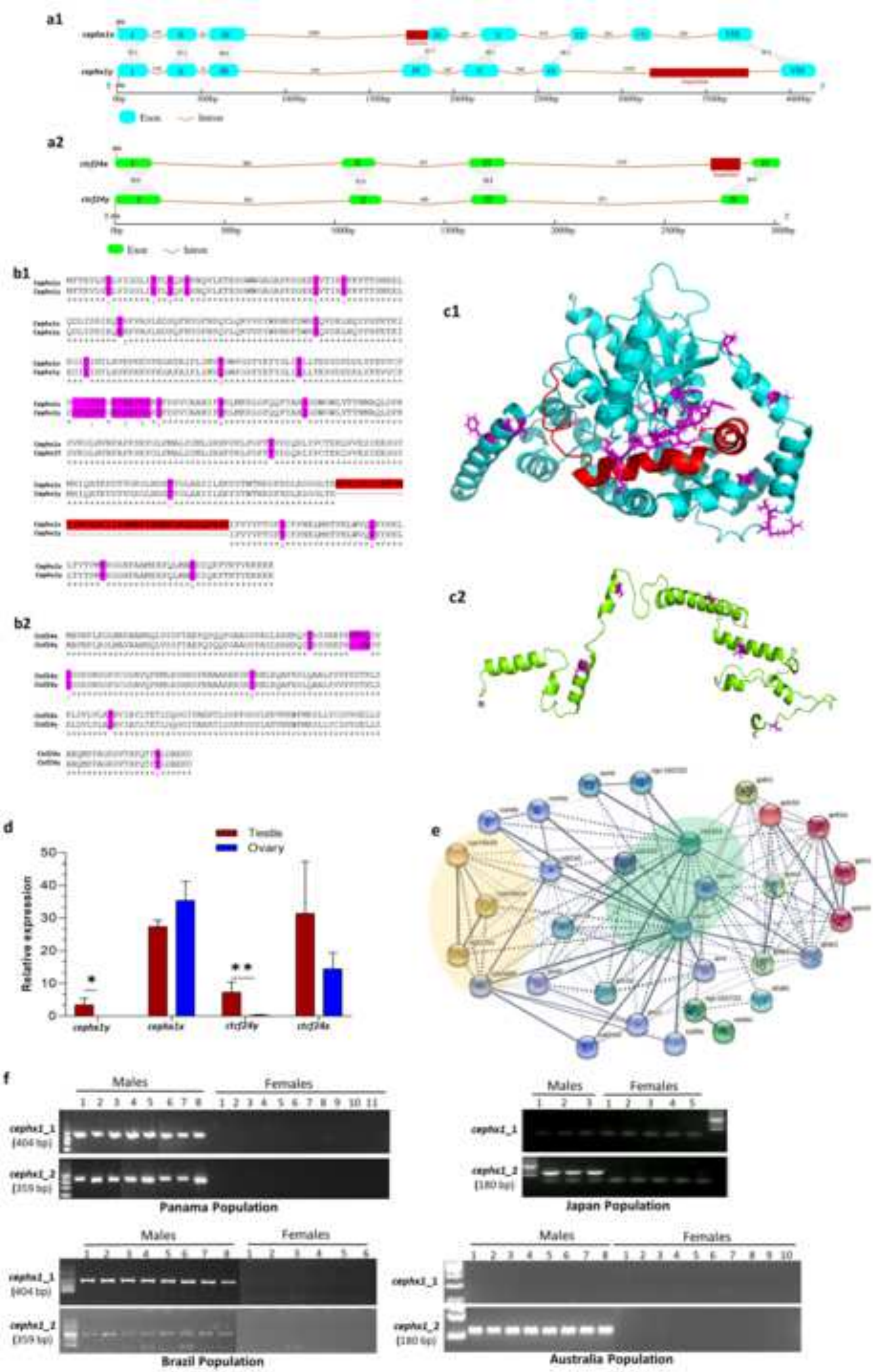
a1
a2
b1
b2
c1
c2
d
Testis
Ovary
Relative expression
cephx1y
cephx1x
ctcf24y
ctcf24x
e
f
Males
Females
Panama Population
Japan Population
Brazil Population
Australia Population
cephx1_1
cephx1_2
(404 bp)
(359 bp)
(180 bp)

Click here to access/download
Supplementary Material
Supplementary File.docx

Click here to access/download
Supplementary Material
Supplementary Tables.xlsx
