# Supplementary material for: Exploring the cobia (Rachycentron canadum) genome: unveiling putative male heterogametic regions and identification of sex-specific markers
Source: Gigascience. 2024 Jul 12;13:giae034. doi: 10.1093/gigascience/giae034 (PMC11240236; doi:10.1093/gigascience/giae034)
Supplement: giae034_Supplemental_Files [file giae034_supplemental_files.zip › Revised Supplementary File (Clean version).docx]

**Supplementary Information for the Paper Entitled**

**Exploring** the cobia (Rachycentron canadum)

**genome: Unveiling putative male heterogametic regions and identification sex-specific markers**

**Table of Contents**

**1. Supplementary notes (Pages 2)**

***Estimation of nenome size (Page 2)***

**2. Supplementary figures for the main text (Pages 3 - 9)**

**3. Supplementary tables for the main text (Pages 10 - 19)**

**4. References (Page 20)**

**5. Genomic DNA sequence alignment of *ephx1* and *tcf24* (Pages 21 - 29)**

**6. Protein sequence alignment of Ephx1 (Pages 30 - 33)**

**1. Supplementary Note**

***Estimation of genome size***

The genome size of the male and female Cobia was estimated using k-mer analysis based calculation by Jellyfish v2.2.6^1^ and GenomeScope v1.0^2^. Distribution of K-mer depth can be used to estimate genome size, heterozygosity and repeat rate.

**Table N1 Summary statistics of the genome size estimate based on** **K-mer analysis.** In male cobia, the K_depth was 117. The genome size of male cobia is 585.72 Mb. In female cobia, the K_depth was 104 and the genome size of female cobia is 588.46 Mb.

| **Sample** | **K-mer** | **K-mer num** | **Genome size (Mb)** | **Clean data (Gb)** | **Heterozygous rate (%)** | **Repeat rate (%)** |
| --- | --- | --- | --- | --- | --- | --- |
| Male | 17 | 68,529,613,815‬ | 585.72 | 87.07 | 0.116 | 32.3 |
| Female | 17 | 61,200,088,248 | 588.46 | 78.12 | 0.101 | 32.7 |

**2. Supplementary Figures for the Main Text**

b)

a)


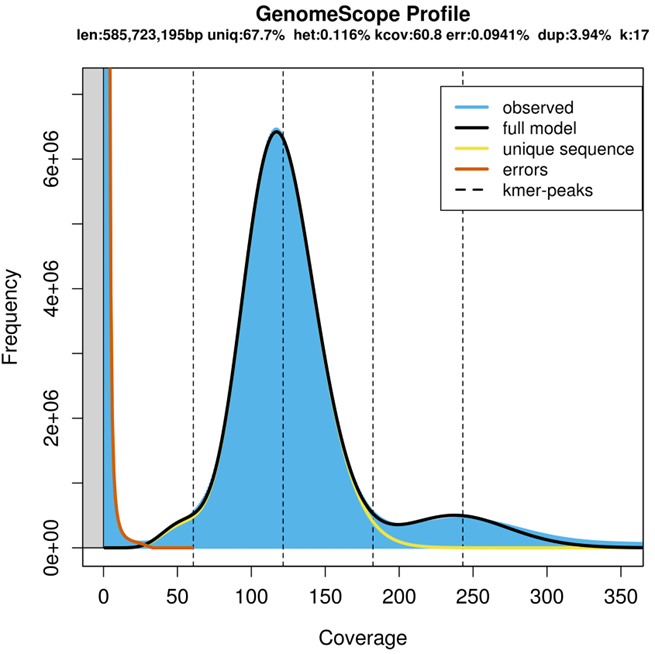

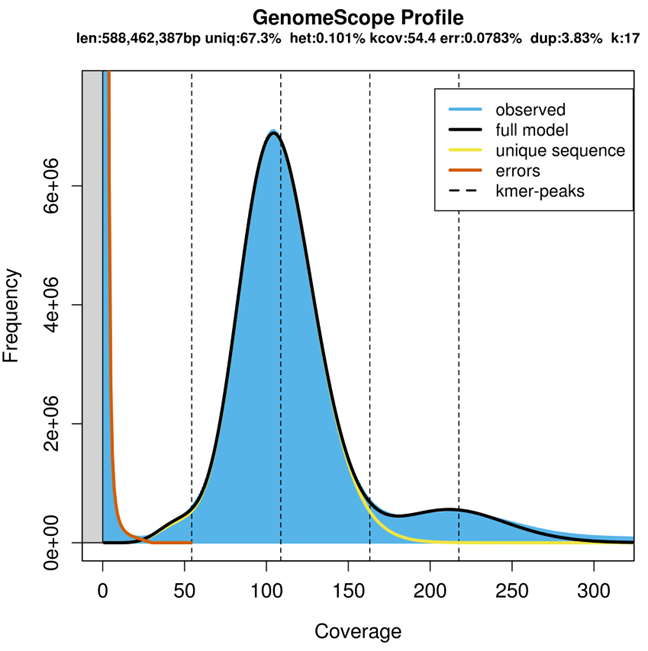


**Figure S1 K-mer depth distribution curve (a: male, b: female).** Horizontal axis: K-mer depth. Vertical axis: frequency of K-mer at specified depth. Blue area represents observed K-mer depth distribution. Area under red line represents low frequency K-mers which are identified as sequencing errors. Reliable K-mers are shown under the black line, which are used to estimate genome size. Vertical dashed lines show peak positions of K-mer depth distribution. Non-repeat fraction is shown under yellow line.

b)

a)


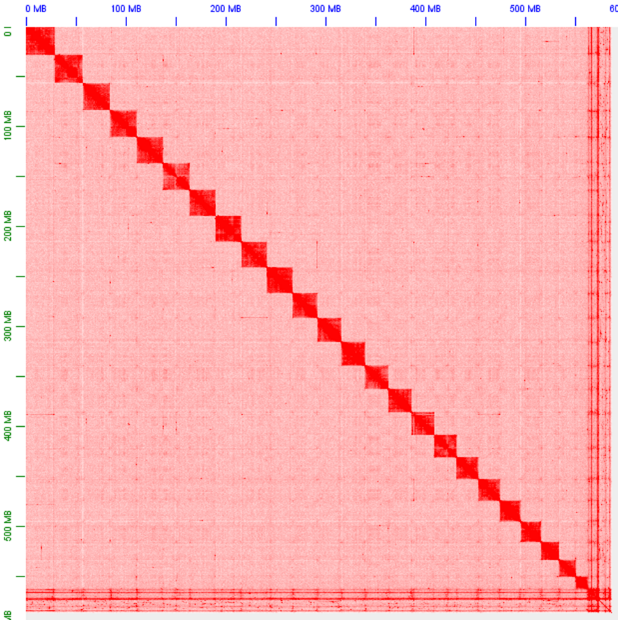

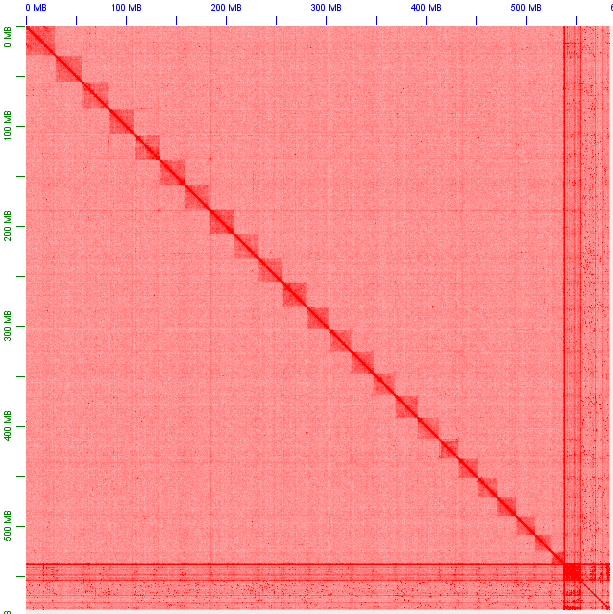


**Figure S2 Chromosome-level assembly of the male and female Cobia genome using Hi-C data (a: male, b: female).** Heatmap of contact metrics generated from mapping of Hi-C reads to genome sequences. The darker the red, the stronger the interaction. Interaction intensity of intra-chromosome are stronger than that of inter-chromosome. Boundaries of chromosomes are obvious.


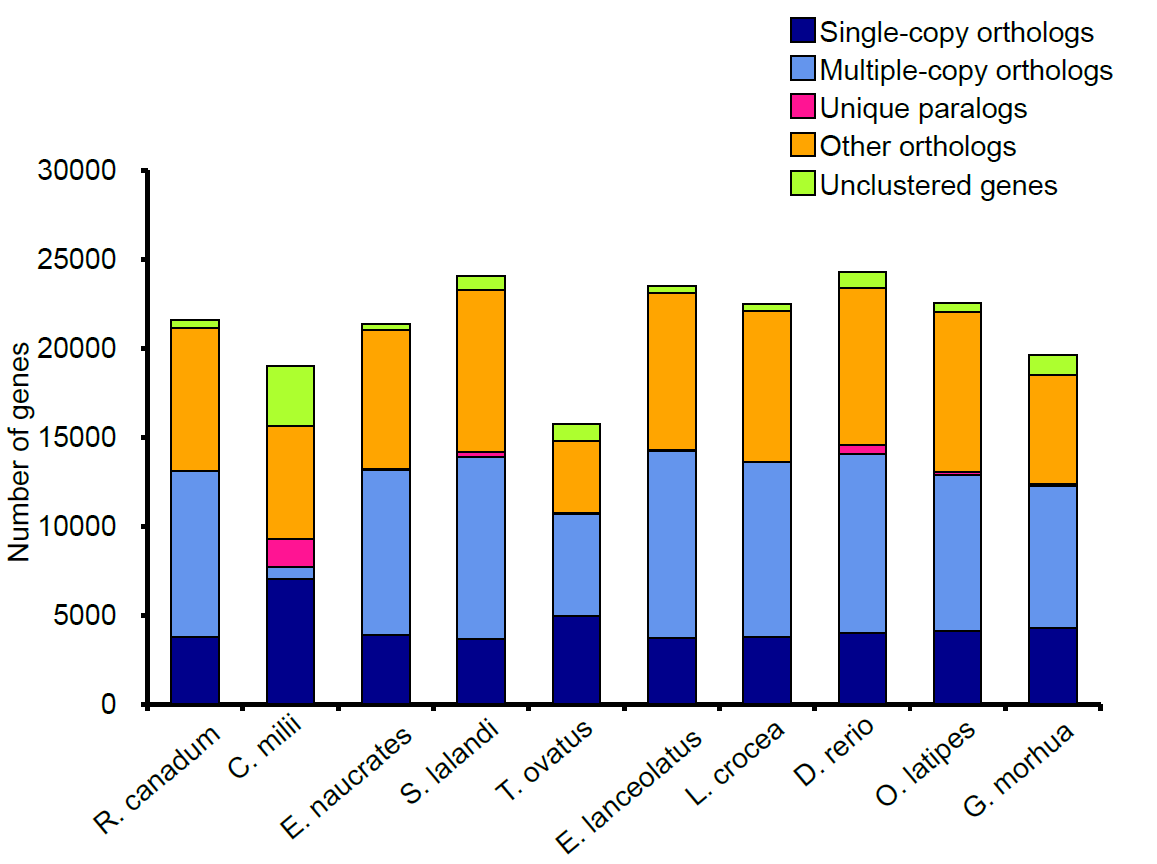


**Figure S3.** Gene family comparison of Cobia and other fish, and single-copy orthologues were used to construct the phylogenetic tree. Statistics of orthologous families for *R.* *canadum* (cobia), *T. ovatus* (Pompano), *S.* lalandi (Yellowtail amberjack), *E. naucrates* (Live sharksucker), *C. milii* (Elephant shark), *L. crocea* (Large yellow croker), *D. rerio* (Zebrafish), *O. latipes* (Medaka), *G. morhua* (Atlantic cod)*,* and *E. lanceolatus* (Giant grouper). Single-copy orthologs represent single-copy genes in the family. Multiple-copy orthologs represent genes with multiple copies in the family. Other orthologs represent the gene families not in all species. Unique paralogs mean that genes belong to the families which existed in only one species. Unclustered genes represent the genes that could not be clustered into gene families.


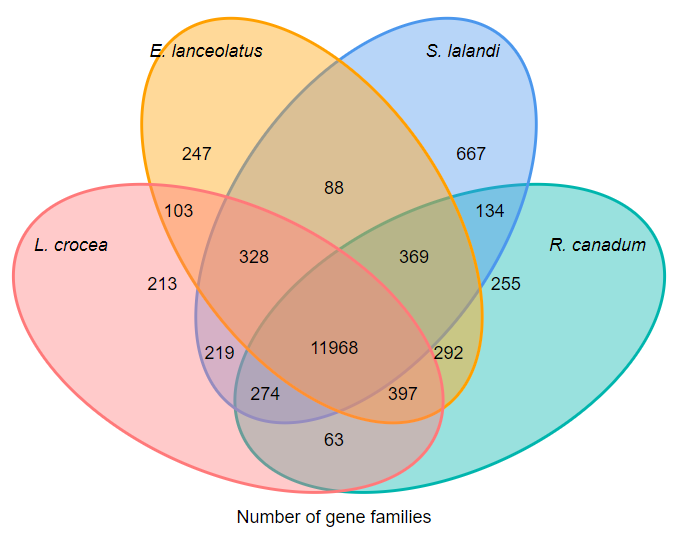


**Figure S4. Venn diagram showing shared orthologous groups for** *R. canadum (Cobia)*, *S. lalandi*, *L. crocea* and *E. lanceolatus*. A total of 11,968 gene families were shared by the four species. And 255 gene families were specific in *R. canadum*.


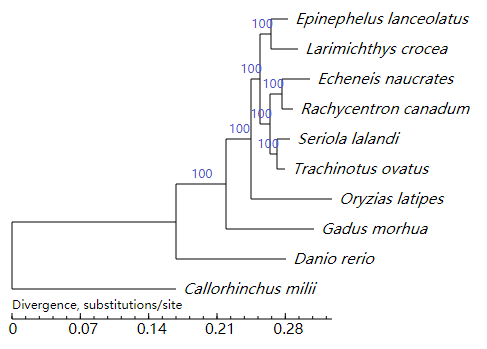


**Figure S5 Phylogenetic tree.** Single-copy families were chosen to construct a phylogenetic tree. *C. milii* as the outgroup.


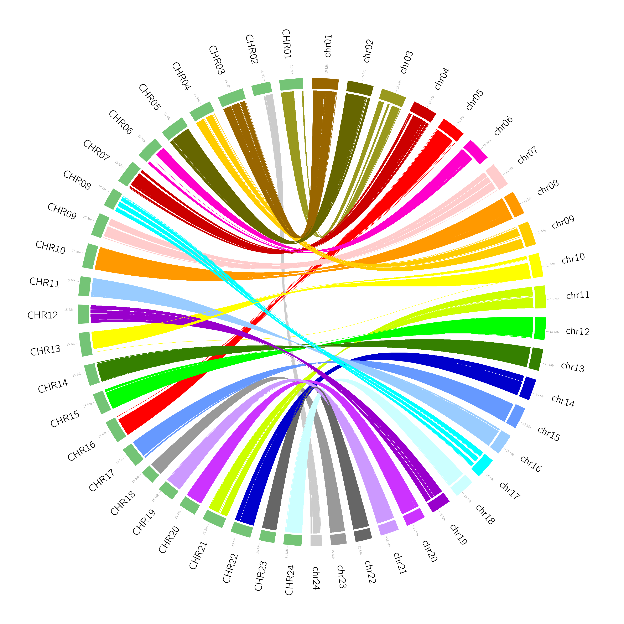

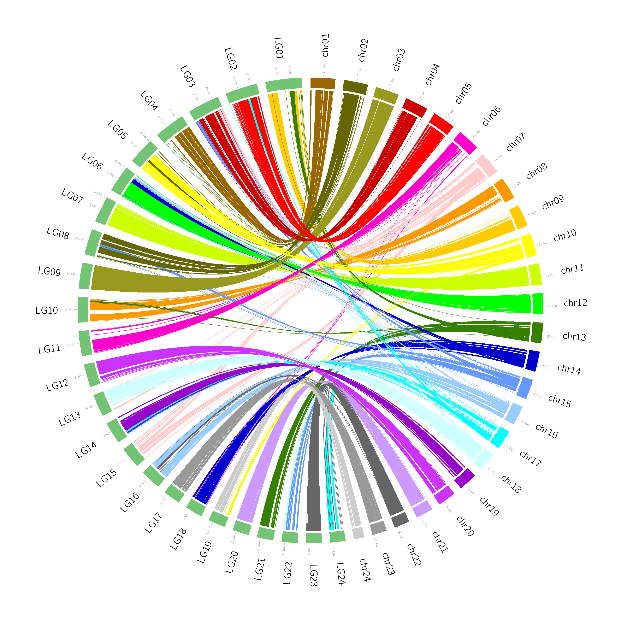


a)

b)

**Figure S6** **Genome comparisons shows 24 chromosomes of *R. canadum* with a one-to-one relationship with 24 chromosomes of the other three** Carangiformes including two Carangidaes of *T. ovatus* and *S.* lalandi, as well as one Echeneidae of *E. naucrates*. **a)** *R. canadum and E. naucrates* (Live sharksucker); **b)** *R. canadum and T. ovatus* (Pompano). Right half round: chromosomes of *R. canadum*. Left half round: chromosomes of *E. naucrates* and *T. ovatus*.


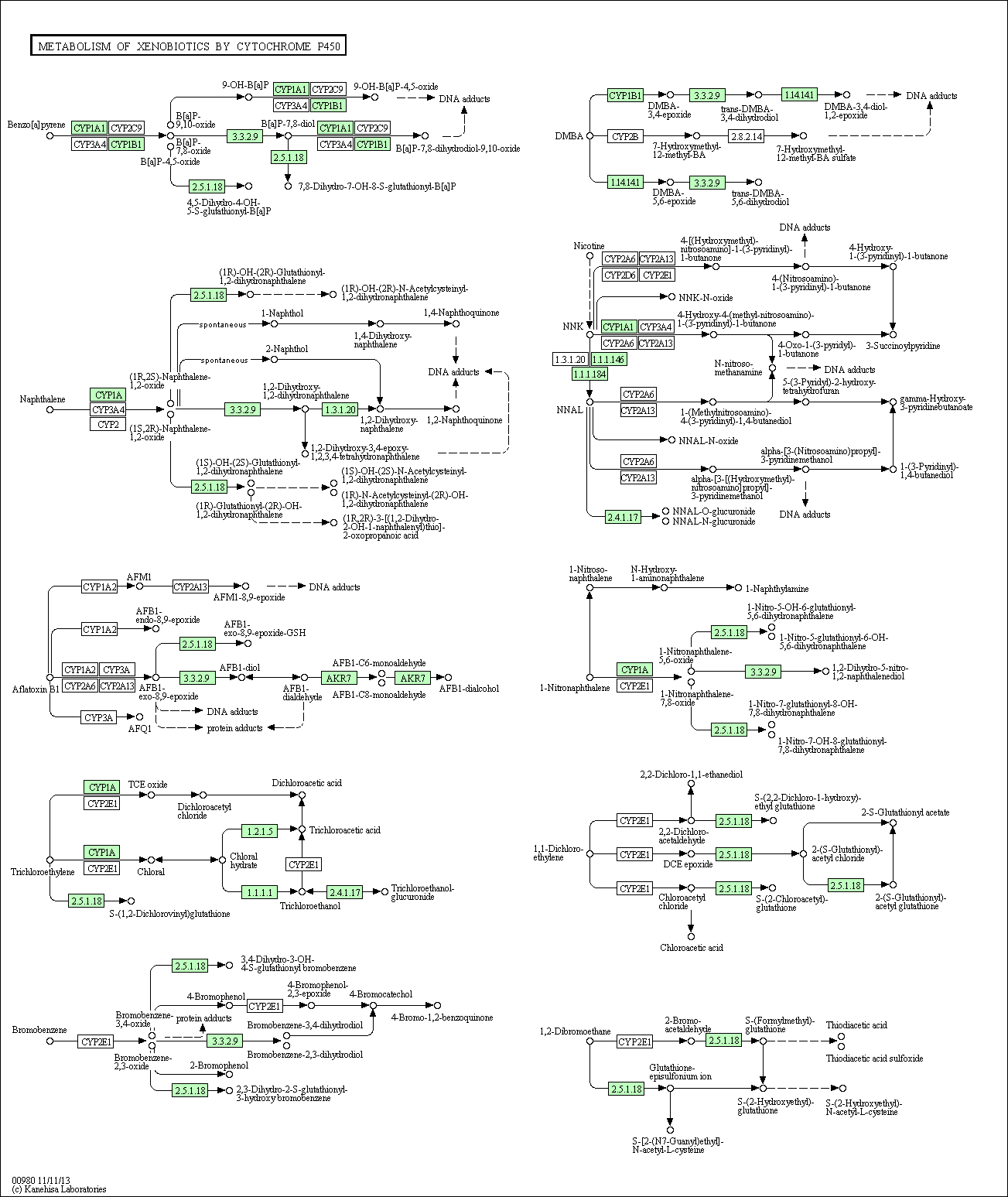


*Cephx1Y*

*Cephx1Y*

*Cephx1Y*

*Cephx1Y*

*Cephx1Y*

*Cephx1Y*

*Cephx1Y*

**Fig. S10**

**Figure S7 The metabolism xenobiotics by cytochrome P450 pathway.**

**3. Supplementary Tables for the Main Text**

**Table S1. Statistics of raw reads and clean reads generated from stLFR**

| Sample | Raw reads (Mb) | Read length (bp) | Clean reads (Mb) | Clean bases (Gb) | Clean Reads Ratio (%) | Depth (X) |
| --- | --- | --- | --- | --- | --- | --- |
|  |  |  |  |  |  |  |
| Male | 662.01 | 100_100 | 435.35 | 87.07 | 34 | 148 |
| Female | 684.32 | 100_100 | 390.6 | 78.12 | 43 | 132 |

Note: Clean Reads Ratio (%): ratio of reads that were filtered.

**Table S2. Statistics of raw reads and clean reads generated from Hi-C**

|  | Male | | Female | |
| --- | --- | --- | --- | --- |
|  | **Reads 1** | **Reads 2** | **Reads 1** | **Reads 2** |
| Total Reads | 403,029,719 | 403,029,719 | 510,961,552 | 510,961,552 |
| Mapped Reads | 358,818,554 | 338,266,081 | 465,404,342 | 454,463,172 |
| Mapping Ratio (%) | 89.03% | 83.93% | 91.08% | 88.94% |
| Valid Pairs | 24,712,101 | | 48,132,481 | |
| Percentage | 6.13% | | 9.42% | |
| Total bases (Gb) | 81.5 | | 103.1 | |
| Depth (X) | 137 | | 173 | |

**Table S3. Summary of male and female cobia genome assembly and annotation**

|  | Draft assembly based on stLFR for Male Cobia | Draft assembly based on stLFR for Female Cobia | Chromosome-Level Scaffolds Based on Hi-C for Male Cobia | Chromosome-Level Scaffolds Based on Hi-C for female Cobia |
| --- | --- | --- | --- | --- |
| Genome Assembly | | | | |
| Length of genome (bp) | 586,231,776 | 583,560,666 | 586,509,684 | 583,884,166 |
| Number of contigs | 17,158 | 18,195 | 17,168 | 18,197 |
| Contigs N50 (bp) | 86,022 | 79,906 | 86,012 | 79,906 |
| Number of scaffold | 6,089 | 6,940 | 5,368 | 6,295 |
| Scaffold N50 (bp) | 10,320,226 | 6,273,840 | 24,262,886 | 22,506,988 |
| GC content is (%) | 40.76 | 40.76 | _a | _a |
| Genome Annotation | | | | |
| Protein-coding gene number | *_a* | | 21,604 | 21,688 |
| Mean transcript length (bp) |  |  | 12,874.91 | 12,466.79 |
| Mean exons per gene |  |  | 10.25 | 10.14 |
| Mean exon length (bp) |  |  | 173.91 | 173.79 |
| Mean intron length (bp) |  |  | 1,198.74 | 1,171.70 |

Note: *_a No data available*

**Table S4. Scaffolding statistics based on Hi-C data for each chromosome of male and female Cobia genome**

| Chromosome ID | Male | | Female | | Matched Percentage (Male Vs Female) |
| --- | --- | --- | --- | --- | --- |
|  | **Length (bp)** | **Percentage** | **Length (bp)** | **Percentage** |  |
| 1 | 28,888,748 | 4.93 | 29,415,270 | 5.04 | 93.26 |
| 2 | 28,004,846 | 4.77 | 25,360,364 | 4.34 | 92.50 |
| 3 | 27,191,039 | 4.64 | 26,824,573 | 4.59 | 94.22 |
| 4 | 26,800,651 | 4.57 | 26,540,736 | 4.55 | 88.32 |
| 5 | 26,557,569 | 4.53 | 25,512,035 | 4.37 | 92.28 |
| 6 | 26,375,439 | 4.50 | 24,371,393 | 4.17 | 91.50 |
| 7 | 26,197,041 | 4.47 | 24,539,579 | 4.20 | 91.48 |
| 8 | 25,890,154 | 4.41 | 24,497,685 | 4.20 | 94.08 |
| 9 | 25,708,093 | 4.38 | 24,105,854 | 4.13 | 91.97 |
| 10 | 25,637,099 | 4.37 | 24,073,601 | 4.12 | 94.31 |
| 11 | 24,442,091 | 4.17 | 25,494,556 | 4.37 | 88.97 |
| 12 | 24,262,886 | 4.14 | 21,752,547 | 3.73 | 91.87 |
| 13 | 23,743,953 | 4.05 | 22,506,988 | 3.85 | 92.37 |
| 14 | 23,386,791 | 3.99 | 21,488,317 | 3.68 | 90.98 |
| 15 | 23,317,458 | 3.98 | 22,276,717 | 3.82 | 92.96 |
| 16 | 22,784,374 | 3.88 | 18,586,124 | 3.18 | 86.74 |
| 17 | 22,257,943 | 3.79 | 22,455,485 | 3.85 | 93.36 |
| 18 | 21,929,055 | 3.74 | 19,294,779 | 3.30 | 91.22 |
| 19 | 21,586,757 | 3.68 | 22,067,774 | 3.78 | 92.23 |
| 20 | 21,093,211 | 3.60 | 19,193,771 | 3.29 | 91.88 |
| 21 | 20,109,627 | 3.43 | 19,314,306 | 3.31 | 95.20 |
| 22 | 18,054,798 | 3.08 | 19,547,493 | 3.35 | 88.24 |
| 23 | 16,763,352 | 2.86 | 16,756,968 | 2.87 | 90.60 |
| 24 | 12,081,543 | 2.06 | 11,291,295 | 1.93 | 91.88 |
| Total anchorded | 563,064,518 | 96.00 | 537,268,210 | 92.02 | - |
| Unkown | 23,445,166 | 4.00 | 46,615,956 | 7.98 | - |
| Total | 586,509,684 | - | 583,884,166 | - | - |

**Note:** Alignment length: 24 chromosomes between females and males

**Table S5. Quality assessment of female and male genome assembly completeness and gene sets with the BUSCO tool**

**(Using Actinopterygii gene set of BUSCO database**^3^.

| Statistical level | Male | | Female | |
| --- | --- | --- | --- | --- |
|  | **Genome assembly** | **Gene set** | **Genome assembly** | **Gene set** |
| Complete BUSCOs (C) | 4,414 | 4412 | 4,395 | 4393 |
| Complete Single-Copy BUSCOs (S) | 4,316 | 4,269 | 4,298 | 4,245 |
| Complete Duplicated BUSCOs (D) | 98 | 143 | 97 | 148 |
| Fragmented BUSCOs (F) | 83 | 121 | 100 | 137 |
| Missing BUSCOs (M) | 87 | 51 | 89 | 54 |
| Total BUSCO groups searched | 4,584 | 4,584 | 4,584 | 4,584 |
| Total | C:96.3% (S:94.2%, D: 2.1%) | C:96.2% (S:93.1%, D:3.1%) | C:95.9% (S:93.8%, D:2.1%) | C:95.8% (S:92.6%, D:3.2%) |

Note: BUSCO version is: 2.0; The lineage dataset is: actinopterygii_odb9 (Creation date: 2016-02-13, number of species: 20, number of BUSCOs: 4,584.

**Table S6. Functional annotation of predicted protein-coding genes of the male and female Cobia genome**

| Values | Male | | Female | |
| --- | --- | --- | --- | --- |
|  | **Number** | **Percentage** | **Number** | **Percentage** |
| Total | 21,604 | 100% | 21,688 | 100% |
| Swissprot-Annotated | 20,561 | 95.17% | 20,700 | 95.44% |
| KEGG-Annotated | 18,600 | 86.10% | 19,375 | 89.34% |
| TrEMBL-Annotated | 21,430 | 99.19% | 21,543 | 99.33% |
| Interpro-Annotated | 19,984 | 92.50% | 20,965 | 96.67% |
| GO-Annotated | 15,673 | 72.55% | 15,743 | 72.59% |
| Overall | 21,446 | 99.27% | 21,555 | 99.39% |

**Table S7. Statistics of repeat elements in the male cobia genome.**

| Type | Repbase TEs | | *De novo* TEs | | TE Proteins | | Combined TEs | |
| --- | --- | --- | --- | --- | --- | --- | --- | --- |
|  | **Length (bp)** | **% of genome** | **Length (bp)** | **% of genome** | **Length (bp)** | **% of genome** | **Length (bp)** | **% of genome** |
| DNA | 19,348,175 | 3.30 | 36,688,646 | 6.26 | 2,617,224 | 0.45 | 44,763,144 | 7.63 |
| LINE | 5,494,546 | 0.94 | 13,015,341 | 2.22 | 2,618,115 | 0.45 | 17,505,532 | 2.98 |
| SINE | 1,552,188 | 0.26 | 1,356,848 | 0.23 | 0 | 0.00 | 2,511,622 | 0.43 |
| LTR | 5,078,740 | 0.87 | 10,433,850 | 1.78 | 506,762 | 0.09 | 14,906,352 | 2.54 |
| Others | 4,101 | 0.00 | 0 | 0.00 | 0 | 0.00 | 4,101 | 0.00 |
| Unknown | 0 | 0.00 | 6,894,148 | 1.18 | 0 | 0.00 | 6,894,148 | 1.18 |
| Total | 26,896,533 | 4.59 | 57,074,416 | 9.73 | 5,650,785 | 0.96 | 64,980,418 | 11.08 |

**Table S8. Statistics of repeat elements in the female cobia genome**

| Type | Repbase TEs | | *De novo* TEs | | TE Proteins | | Combined TEs | |
| --- | --- | --- | --- | --- | --- | --- | --- | --- |
|  | **Length (bp)** | **% of genome** | **Length (bp)** | **% of genome** | **Length (bp)** | **% of genome** | **Length (bp)** | **% of genome** |
| DNA | 19,424,351 | 3.33 | 38,662,382 | 6.62 | 2,587,774 | 0.44 | 46,125,413 | 7.90 |
| LINE | 5,478,858 | 0.94 | 14,029,631 | 2.40 | 2,606,193 | 0.45 | 17,828,304 | 3.05 |
| SINE | 1,553,700 | 0.27 | 566,915 | 0.10 | 0 | 0.00 | 2,038,845 | 0.35 |
| LTR | 5,115,636 | 0.88 | 9,462,010 | 1.62 | 523,089 | 0.09 | 13,993,564 | 2.40 |
| Others | 3,901 | 0.00 | 0 | 0.00 | 0 | 0.00 | 3,901 | 0.00 |
| Unknown | 0 | 0.00 | 10,210,768 | 1.75 | 0 | 0.00 | 10,210,768 | 1.75 |
| Total | 26,971,669 | 4.62 | 60,028,745 | 10.28 | 5,583,227 | 0.96 | 67,448,012 | 11.55 |

**Table S9. Non-coding RNA prediction in the male and female cobia genome**

| Type | | Copy(w*) | | Average length(bp) | | Total length(bp) | | % of genome | |
| --- | --- | --- | --- | --- | --- | --- | --- | --- | --- |
|  |  | **Male** | **Female** | **Male** | **Female** | **Male** | **Female** | **Male** | **Female** |
| miRNA | | 263 | 248 | 82.441065 | 82.32258 | 21,682 | 20,416 | 0.003697 | 0.003497 |
| tRNA | | 704 | 715 | 76.316761 | 76.20559 | 53,727 | 54,487 | 0.00916 | 0.009332 |
| rRNA | **rRNA** | 95 | 100 | 116.863158 | 136.96 | 11,102 | 13,696 | 0.001893 | 0.002346 |
|  | **18S** | 56 | 57 | 105.125 | 105.24561 | 5,887 | 5,999 | 0.001004 | 0.001027 |
|  | **28S** | 26 | 30 | 164.923077 | 215.9 | 4,288 | 6,477 | 0.000731 | 0.001109 |
|  | **5.8S** | 3 | 3 | 43.333333 | 82 | 130 | 246 | 0.000022 | 0.000042 |
|  | **5S** | 10 | 10 | 79.7 | 97.4 | 797 | 974 | 0.000136 | 0.000167 |
| snRNA | **snRNA** | 242 | 226 | 125.640496 | 125.71681 | 30,405 | 28,412 | 0.005184 | 0.004866 |
|  | **CD-box** | 107 | 108 | 100.485981 | 96.90741 | 10,752 | 10,466 | 0.001833 | 0.001792 |
|  | **HACA-box** | 70 | 67 | 147.757143 | 151.56716 | 10,343 | 10,155 | 0.001763 | 0.001739 |
|  | **splicing** | 57 | 43 | 133.122807 | 141.13954 | 7,588 | 6,069 | 0.001294 | 0.001039 |
| Total | | 1,304 | 1,289 | _a | _a | 116,916 | 117,011 | _a | _a |

**Table S10. Statistics of male and female cobia resequencing**

**See separate excel sheet**

**Table S11. 162 SNPs strongly associated with sex at the peak region on Chr18 detected by genome wide association studies**

**See separate excel sheet**

**Table S12. GWAS detected suggestive signals of sex association at Chr4, Chr5 and Chr17**

**Table S13. Genes annotated in reassembled Male chromosome 18 (MChr18)**

**See separate excel sheet**

**Table S14. 231 SNPs strongly associated with sex at the peak region on Chr18 detected by genome wide association studies**

**See separate excel sheet**

**References**

1. Marçais G, Kingsford C. A fast, lock-free approach for efficient parallel counting of occurrences of k-mers. *Bioinformatics* **27**, 764-770 (2011).

2. Vurture GW*, et al.* GenomeScope: fast reference-free genome profiling from short reads. *Bioinformatics* **33**, 2202-2204 (2017).

3. Simão FA, Waterhouse RM, Ioannidis P, Kriventseva EV, Zdobnov EM. BUSCO: assessing genome assembly and annotation completeness with single-copy orthologs. *Bioinformatics* **31**, 3210-3212 (2015).

***cephx1y* and *cephx1x* Genomic Sequence Alignment**

*cephx1y* ATGTTCACAGAGGTCCTGGTTGATCTAGTGATTGGAGGACTTATCGTCTTTCTGATTCAG

*cephx1x* ATGTTCACAGAGGTCCTGGTTGGTCTAGTGATTGGAGGACTTATCTTCTTTCTGGTTCAG

********************** ********************** ******** *****

*cephx1y* AGGAGAAGGAACCAGGTTCTGAAGACAGAGGATGGCTGGTGGGGGGCTGGAGCACCCCCT

*cephx1x* AGGAGCAGGAACCAGGTTCTGAAGACAGAGGATGGCTGGTGGGGGGCTGGGGCACCGCCT

***** ******************************************** ***** ***

*cephx1y* GATGGCGAGGAGGAGGTCACCATCCGTTCATTTAAAGTCACCACTAGTAATGAAGAGCTG

*cephx1*x GATGGCGAGGAGGACGTCACTATCCGTCCATTTAAAGTCACCACTAGTAATGAAGAGCTG

************** ***** ****** ********************************

*cephx1y* CAGGTCAGATACCCTCATAGTTTGATAGTCCAGATAGATCTATTATGAGGTAGTTCCACT

*cephx1*x CAGGTCAGATTCCCTTATAGTTTGATAGTCCAGATAGATCTAGTATGAGGTAGATCCACT

********** **** ************************** ********** ******

*cephx1y* GAACAGTCTAAAAAATTGCAGTAGTCCTCACATTTTTTCTCTTGCCTGTCAGGACCTATA

*cephx1*x GAACAGTCTAAAAGATTGCAGTAGGCCTCACATTTTTCCTCTTGCCTGTCAGGACCTATA

************* ********** ************ **********************

*cephx1y* TAGTCGGATAGAGCAGATGCACCCTGTTGCCTCATTGGAGGACAGTCAGTTCAATTATGG

*cephx1*x TAGTCGGATAGAGCAGACGCGCCCTGTTGCCTCATTGGAGGACAGCCAGTTCAATTATGG

***************** ** ************************ **************

*cephx1y* TTTCAACTCCCAGTATCTGCAGAAGGTGGTCTCTTACTGGAGAAATGACTTTGACTGGAG

*cephx1*x CTTCAACTCCCAGTATCTGCAGAAGGTGGTCTCTTACTGGAGAAATGACTTTGACTGGAG

***********************************************************

*cephx1y* AAGACAAGTTGACAAACTCAACCAGTACCCCCACTTTAAAACTAAAATTGAAGGTGAGGC

*cephx1*x AATACAAGTTGACAAACTCAACCAGTACCCCCACTTTAAAACTAAAATTGAAGGTGAGGC

** *********************************************************

*cephx1y* TGCATTGATGACAAGTTGATGAAGAACATATTGTCCTGTTCTTACTTATTGAACCTCCAC

*cephx1*x TGCATTGATGACAAGTTGATGAAGAACATATTGTCCTGTTCTTACTTATTGAACCTCCAC

************************************************************

*cephx1y* ACAGGCATTTATATCCATTACCTGCATGTGAAGCCTAAGAAGGTGCCAGAGGGAGCTACT

*cephx1*x ACAGGCATTGATATCCATTACCTGCATGTGAAGCCTAAGAAGGTGCCAGAGGGAGCTACT

********* **************************************************

*cephx1y* GCTATTCCTCTGATAATGGTCCAAGGCTGGCCTGGCTCCTTCTATGAGTTCTATGGGTTG

*cephx1*x GCTATTCCTCTGATAATGGTCCACGGCTGGCCTGGCTCCTTCTATGAGTTCTATGGGTTG

*********************** ************************************

*cephx1y* ATCCACCTGCTGACAGAACCATCAGACCCAGATGACCTTGTGTTTGAGGTGGTGTGTCCC

*cephx1*x ATCCCCCTGCTGACAGAACCATCAGACCCAGATGACCTTGTGTTTGAGGTGGTGTGTCCC

**** *******************************************************

*cephx1y* TCC--------GTATGGCTTCTCTGAAGCACCACGTAAGAAAGGTGAGTCTGGGAGGTTT

*cephx1*x TCCATACCAGGGTATGGCTTCTCTGAAGCACCACGTAAGAAAGGTGAGTCTGGGAGGTTT

*** *************************************************

*cephx1y* CTTGCAGAAAGGAAGGAAGTTGGACTTTCCCTATAAAACCATAGAGTTCAGCATACAGCT

*cephx1*x CTTGCAG---GACATGAAGTTGGACTTTCCCTATAAAACCATAGAGTTCAGCATACAGCT

******* * * *********************************************

*cephx1y* GAATCAGTCAATTAGGATAGAAGGAGTCTAGGAAACAGATAACATGTACAGTATGTGCAA

*cephx1*x GAATCAGTCAATTAGGAT---AGGAGTCTAGGAAACAGATAACATGTACAGTATGTGCAA

****************** ***************************************

*cephx1y* GGCAAGT-GCAAAACAGCACTGATCAGAAAAGTAATGGTTCCAGATCAACTCCAAACAGA

*cephx1*x GGCTACTGGAAAAACAGCACTGATGAGAAGAGTAATGGTTCCAGATCAGCTCCAAACAGA

*** * * * ************** **** ****************** ***********

*cephx1y* TTTCTTTGAATGACTAATCCCAGGTTTCAACTGAACAGATATGGTGAGATGGAAACGTCC

*cephx1*x TTTCTTTGAATGACCAATCCCAGGTTTCAACTGAACAGATATGGTGAGATGGAAATGTCC

************** **************************************** ****

*cephx1y* TTTAAAAAAAAAACCAAAAAACAAAAAAAAAAAACACATAAATTCATCCATCTATTATTT

*cephx1*x TTTAAAAAAAAA---AAAAAAC----AACAACAACACATAAATTCATCCATCTATTATTT

************ ******* ** ** ****************************

*cephx1y* ATACCACATATACTTATAGGAGCCACTCCCAGCTAACAGTGGGTGACGGTGGGATACACC

*cephx1*x ATACCACATATACTTATAGGAGCCACTCCCAGCTAACAGTGGGTGATGGTGGGATACACC

********************************************** *************

*cephx1y* CTGGACAGGTCACCAAACAACCTCACATTCACACCTTTGGGCGTTCTTAGAGTCACCAGT

*cephx1*x CTGGACAGGTCACCAAACAACCTCACAT------------------------TCACCAGT

**************************** ********

*cephx1y* TACTCGAACATGCATGTCTTTAGACTGTGGGAGGAAGCTGTAGTACCCGGAGGCAACCCT

*cephx1*x TACTCGAACCTGCAGTCTTTAGACTGTGGGAGGAAGCTTTAGTACCCGGAGGCAACCCT

********* ***************************** ********************

*cephx1y* CTCAGGCACAGGGAGAAACATGAAACTCTACATAAAAAGGCC----------CTTGCTGT

*cephx1*x CTCAGGCACAGGGAGAAACATGAAACTCTACATAAAAAGGCCCCAGAACCTTCTTGCTGT

****************************************** ********

*cephx1y* GAGGCCA----------CACTGCACCACTGAACCACTTTTTGTACCAAGTTC----GTGC

*cephx1*x GAGGCCACAGTGCTAACCACTGCACCACTGAACCACTTTCTGTACCAAGTTCATGGGTGC

******* ********************** ************ ****

*cephx1y* CTTAAAAACCAATGACAAAGTCACGATTAAATGCAGATCACGGAAGCAGAGCTGGTTCCA

*cephx1*x CTTAAAAACCAGTGACAAAGTCACGTTTAAATGCAGATCACGGAAGCAGAGCTGGTTCAA

*********** ************* ******************************** *

*cephx1y* TCCAAAGATTTGTTCCACAAAGAGAAAAACAAACAAACAAAGCAAAACACATTCTGTCTC

*cephx1*x TCCAAAGATTTGTTCCACAAAGAAAAA-----------AAAACAAAACACATTCTGT---

*********************** *** *** ***************

*cephx1y* ACTTAAGGGGAGAGGCAAAGTCTCAACCAGAGCAAAAAGAAACCCAAAATCCATAAAGAA

*cephx1*x ------------------------------------------CATAAAATCCATAAAGAA

* ***************

*cephx1y* GACAACTAGTCCAGAAACACAATGGAAAACACAGGGAAACGAACAGGAGCAAAAAACAGA

*cephx1*x GACAACTAGTCCAGAAACACAATAGAAAACACAGGGAAACGAACAGGAGCAAAAAATAGA

*********************** ******************************** ***

*cephx1y* TAAAGGGACAATTGCACACAGGTGAGACACATTAGGGCAGGGCAGACTCACAATGGAGGG

*cephx1*x TAAAGGGACAATTGAGCACAGGTGAGACACATTAGGGCAGGGCAGACTCACAATGGAGGG

************** ********************************************

*cephx1y* AAACGGGACAGACA-GAAGTGAAACAACCAAGACACACAAGGGAAGGGATTTCAAAGTAA

*cephx1*x AAACAGGACAGACAGGAAGTAAAACAACCAAGACACACAAGGGAAGGGATTTCAAAGTAA

**** ********* ***** ***************************************

*cephx1y* AACAGGAAATCGGCAAGAATTCAAACTAAAGTC---------------------------

*cephx1*x AACAGGAAATCAGCAAGAATTCAAACAAAAGTCTCTTCAAATAGTCCTATCAGGGTCAGG

*********** ************** ******

*cephx1y* ------------------------------------------------------------

*cephx1*x AATATTGCAGGGCTATTGCATTAGCTCATTTTATATTTAGAAAATTAAAGGTATAAACTG

*cephx1y* ------------------------------------------------------------

*cephx1*x ACTACACTTGTGTCTATACGATCTAGATACAAAGAAATAAAAATGATACAATTCTAAAGT

*cephx1y* -------------------TTTTGATTCGGTTTGTGCAGCAAACATCTTCCACAAACTTA

*cephx1*x GTGTTTGTGTGTGTGCAGGTTTTGATTCGGTTTGTGCAGCAAACATCTTCAACAAACTTA

******************************* *********

*cephx1y* TGAAGCGTCTGGGCTTCCAGCAGTTCTACGCTCACAGAGGAGACTGGGGCTGGCTGGTCA

*cephx1*x TGAAGCGTCTGGGCTTCCAGCAGTTCTACGCTCACGGAGGAGACTGGGGCTGGCTGGTCA

*********************************** ************************

*cephx1y* CCACCAACATGGCTCAGCTGGACCCCAAGTAAAATACACAAGAACTGCCTCATCAACCGA

*cephx1*x CCACCAACATGGCTCAGCTGGACCCCAAGTAAAATACACAAGAACTGCCTCATCACCCAA

******************************************************* ** *

*cephx1y* AAATGTTTATTGTGCTTATAGTTTCACTAACACAACCACTGGAGACACCAGACCTTTGAT

*cephx1*x AAATGTTTATTGTGCTTATAGTTTCACTAACACAACCACTGGAGACACCAGACCTTTGAT

************************************************************

*cephx1y* GATATGTAAACGTATGTGTACACTGTCTTTTGTAAATGCTTATATAATGTAGCACTACCT

*cephx1*x GATATGTATATGTATGTGTACACTGTCTTTTGTAAATGCTTATATAATGTAGCACTACCT

******** * *************************************************

*cephx1y* TGTGTCTTGCTTTTGTTTTTTTCTCCACACCCAGGTCAGTCAAAGGCTTGCATGTGAACT

*cephx1*x TGTGTCTTGCTTTTATGTTTTTCTCCACACCCAGGTCAGTCAAAGGCTTGCATGTGAACT

************** * *******************************************

*cephx1y* TTGCTCCACCCTCCAAGCCTGGTCTGCCCATGGCTTTATCTATCATGCTTGGCCGCCACT

*cephx1*x TTGCTCCACCCTCCAAGCCTGGTCTGCCCATGGCTTTATCCATCATGCTTGGCCGGCACT

**************************************** ************** ****

*cephx1y* TCCCTAAGCTCTTTGGCTTCACTGTCGTAGATATTCAGCGTCTCTACCCTTGCACAGAGA

*cephx1*x TCCCTAAGCTCTTTGGCTTCACTGACGTAGATATTCAGCGTCTCTACCCTTGCACAGAAA

************************ ********************************* *

*cephx1y* AACTGGTGGTGGAGTCCATCAAAGAGTCTGGCTACATGCACATCCAGGCCACCAAGCCTG

*cephx1*x AACTGGTGGTGGAGTCCATCAAAGAGTCTGGCTACATGCACATCCAGGCCACCAAGCCTG

************************************************************

*cephx1y* ACACAGTGGGTAAGGACCTGGGGCCCATTTCACCAAAGTTTCTGCATGCTAATCAAAAAA

*cephx1*x ACACAGTGGGTAAGGACCTGGGGCCCATTTCACCAAAGTTTCTGCATGCCAATCAAAAAA

************************************************* **********

*cephx1y* AAGCTTTTATTTGGTACCTTACTCAAGCAAGCAAATGCAACAGTTGAGCACTTATAAAAT

*cephx1*x AAGCTTTTATTTGGTACCTTACTCAAGCAAGCAAATGCAACAGTTGAGTACTGGTAAAAT

************************************************ *** ******

*cephx1y* GACATTGATTTCAGTGATGATAACTAAAACTACAACCAGCCAGCACAG------------

*cephx1*x GACATTGATTTCAGTGATGATAACTAAAACTACAACCAGCCAGCGCAGATGTTTGTAGTC

******************************************** ***

*cephx1y* -----------------------------------------------ATCACTGATTTAC

*cephx1*x TCCCACACTGGTTATAGTAAGGTTTAAAAAGATTAATGGGATCTTCAATCACTGATTTAC

*************

*cephx1y* AATCACAACAAAACATGTAAATAGTTTTTATTGATCAACAAATCCATATCAATTATACAC

*cephx1*x AATCAC-ACGAAATGTGTAAATAGTTTTTATTGATCAACAAATCCATATCAGTCATACAC

****** ** *** ************************************ * ******

*cephx1y* TTTGTGACTGTGTGTTTTCCCTGAGGTCGAGGACTGAATGACTCTACAGTGGGTCTGGCA

*cephx1*x TTTGTGACTGTGTGTTTTCCCTGAGGTCGAGGACTGAATGACTCTCCAGTGGGTCTGGCT

********************************************* *************

*cephx1y* GCCTACATCCTGGAGAAGTTCTCCACATGGACGAACCGTGACTTCAGGGACCTGGAGGAT

*cephx1*x GCCTACATCCTGGAGAAGTTCTCCACATGGACGAACCGTGACTTCAGGGACCTGGAGGAT

************************************************************

*cephx1y* GGAGGACTCACCAGGTAAACAGTGTAGAACAGGAGTCAGGTGTGATTGGTGCCATCACTA

*cephx1*x GGAGGACTCACCAGGTAAACAGTGTAGAACAGGGGTCAGTGCTGATTGGTGCCATCACCA

********************************* ***** **************** *

*cephx1y* CATCCTTCATATCAATATGTGGTATCAAAC--TCATTTACTAGTAATAATACTAGTAGTA

cephx1x CATCCTTCATATCAAGATGTGGTATCAAACTATCATTTACTAGTAATAATAGTAGTAGTA

*************** ************** ******************* ********

*cephx1y* GCATATGTATTAGCAGTGGCCCCCAGGACTGTTTGTCCATAGTAGGAAAATGCG------

*cephx1*x GCATATGTATTAGCAGTGGCCCCCAGGACTGTTTGTCCATAGTAGGAAAATGCGTCTGTT

******************************************************

*cephx1y* ---------------------GACCATA----GGAATCTCTATGGTCATTACTGTAATAT

*cephx1*x CCTCCAGTATCTCAGCTCAGTGACCTCAAAGCAGAATCTCTATGGTCATTACTGTAATAT

**** * ***************************

*cephx1y* TACCTCTTGTTTGTGCTTTGTGTTGAAGGAATTTCTCTCTGGACGACCTGCTGACAAATG

*cephx1*x TACCTCTTGTTTGTGCTTTGTGTTGAAGGAATTTCTCCCTGGACGACCTGCTGACTAATG

************************************* ***************** ****

*cephx1y* TTATGATCTATTGGGTGTCTGG-------ATCTCATCTATGAGGTTCTACAAGGATAACT

*cephx1*x TCATGATCTATTGGGTGTCTGGATGCATCATCTCATCTATGAGGTTCTACAAGGAAAACT

* ******************** ************************** ****

*cephx1y* TTGGCAAAGGGCTTGACCAGCTGCACTCTAAGTGAGTGACAACCAAAGACTTAAATGCAA

*cephx1*x TTGGCAAAGGGCTTGACCAGCCGCACTCTAAGTGAGTGACAACTAAAGACTTAAATGCAA

********************* ********************* ****************

*cephx1y* GCAACATGTTACACTGAGGCTACGTCCACAACACAATGCTTTAGTTTTAAAATGTATCAG

*cephx1*x GCAACATGTTACACTGAGGCTACGTCCACACCACAATGCTTTAGGTTTAAAATGTATCAG

****************************** ************* ***************

*cephx1y* CTATTGATGGTCCACAGCACCCACTGCAGTGTCGGAGAGCAACAACAATTGATCGGCTAG

*cephx1*x CTATTGATGGTCCACAGCACCCACTGCAGTGTTGGAGAGCAACAACAATTGATCGGCTCG

******************************** ************************* *

*cephx1y* CAAATATCAATCTGTGTCGCTCTTGGTTGTTATATGTAGCATGTTGTTTATAGATCAGAA

*cephx1*x CAAATATCAATCTGTGTCGCTCTGGGTTGTTATATGTAGCATGTTGTTTATAGATCAGAA

*********************** ************************************

*cephx1y* AAAATCTAATTTACTACATATGTAGTGAAGAAACATAAGCACTAGATGTGACTGATAAAG

*cephx1*x AAAATCTAATTTAC----------------------------------------------

**************

*cephx1y* TCACATATAATCCTTACAAAGATGTGAAAAAAATCCAACATTTCAAGATCAACAGGTTGT

*cephx1*x ------------------------------------------------------------

*cephx1y* GTGGCGTCCATAGGCTGTATATAAAGATGGACATAGATTGTACATCTATCAGATTGTACT

*cephx1*x ------------------------------------------------------------

*cephx1y* GGAGTCTATGTGAAAATGTCCCTACTTCTTACTTGATTTATTACCTCAATAAAGGTTTTC

*cephx1*x ------------------------------------------------------------

*cephx1y* CTAATGAGTTTATGATCCACTAATCAGCTGTACCATCTCCAAATATGGTTTCTTCTGGTT

*cephx1*x ------------------------------------------------------------

*cephx1y* TCAAAAACCCAAAATGTGATGGCTAGTTTAGAAAATGACAGCTCACAAACTAATGATGCC

*cephx1*x ------------------------------------------------------------

*cephx1y* ATCATAAAGATGTATTAGTTTCTTAGAGGGTACTTTCATTATTTCATGATTGGATGACTT

*cephx1*x ------------------------------------------------------------

*cephx1y* TGAAAGAAATAATTTTATGAATACAAAAATACTTTTGAATCTTACGTGTTTATTGGTTAG

*cephx1*x ------------------------------------------------------------

*cephx1y* ATATCAGGATGTCCCCATGAAGTGGTGACTGGGCATTGATGGCATTTCTTTTAACTGTAA

*cephx1*x ------------------------------------------------------------

*cephx1y* GTTGCTATTTGTGATGATCTATACTTTACAGTATATCATCCTGGTTTAGACAACTAATAT

*cephx1*x -----------------TCTATAGCTTACAGTATATCATCCTGGTTTAGACACTTAATAT

****** *************************** ******

*cephx1y* AAACTGAACCTAAATATTATTGGCTTTTCTAACAGTTGCATCACACCAAGGGGCGGCTTG

*cephx1*x AAACTGAACCTAAATATTATTGGCTTTTCTAACAGTTGCATCACACCAATGGGCGG----

************************************************* ******

*cephx1y* TTCACATCTTGGAATTTTACATAATCCAGAAGCTTGTGACAGTAATGATACTGGTTTCAT

*cephx1*x ----------------------------------TGTGACAGTAATGACACTGGTTTCAT

************** ***********

*cephx1y* GCAGTAAAGGCTCTTAAATAACATCATCACCATCTCTTCCCAGGATACCGGTCTATGTTC

*cephx1*x GCAGTAAAGGCTCCTAAATAACATCATCACCATCTCTTCCCAGGATACCGGTCTATGTTC

************* **********************************************

*cephx1y* CCACTGGGTTTTCCTGCTTCCCCAATGAGCTGATGCACACACCCAAACTGTGGGTCCAAC

*cephx1*x CCACTGGGTTTGCCTGCTTCCCCAACGAGCTGATGCACACACCCAAACTGTGGGTCCAAC

*********** ************* **********************************

*cephx1y* GGAAATACCGTAAACTCCTCACCTATACTCCCATGGTCCGCGGTGGCCATTTTGCTGCCA

*cephx1*x AGAAATACCGTAAACTCCTCACCTATACACCCATGAGCCGCGGTGGCCATTTTGCTGCCA

*************************** ****** ***********************

*cephx1y* TGGAGGAGCCCCAGCTGATGGCCAAGGACATCCAGAAGTTCACCAAGACAGTGGAGAAGA

*cephx1*x TGGAGGAGCCCCAGCTGATGGCCGAGGACATCCAGAAGTTCACCAAGACAGTGGAGAAGA

*********************** ************************************

*cephx1y* AAAAGAAATAG

*cephx1*x AAAAGAAATAG

***********

***ctcf24x* and *ctcf24y* Genomic Sequence Alignment**

*ctcf24x* ATGGCGGTTAATCCTCTGGAAGGTCTCATGGCAGTTGCAGCGAACCACCAGCTGGTGTCC

*ctcf24y* ATGGCGGTTAATCCTCTGGAAGGTCTCATGGCAGTTGCAGCGAACCACCAGCTGGTGTCC

************************************************************

*ctcf24x* AGCCCCACAGCGGAGCCTCAGAGCCAGCAGCCCGGCGCAGCCGGCAGCCCAGCATCCCTG

*ctcf24y* AGCCCCACAGCGGAGCCTCAGAGCCAGCAGCCCGGCGCAGCCGGCAGCCCAGCGTCCCTG

***************************************************** ******

*ctcf24x* CGCAGACACAGGCAGAGCACACGCAGCAGCCGGGAGCCCAGGAAGTGGGTGAGTCTGTTA

*ctcf24y* CGCAGACACAGGCAGAGCATACGCAGCAGCCGGGAGCCCAGGAAGTGGGTAAGTCTGTTA

******************* ****************************** *********

*ctcf24x* ACGGTTCAATTTAAAGACAGTGGGGTACAACAGGAAGCAAAGTATAGAAACCAAAT----

*ctcf24y* ACGGTTCAATTTAAAGACAGTGGGGTACAACAGGAAACAAAGTATATAAGCCAAATTTTC

************************************ ********* ** ******

*ctcf24x* -------AAAATCTCATGTGGGATGTGACACTTTGGCATCAGAGAGCAGCTTACAAAGTT

*ctcf24y* CATTAAGAAAAC----TGAGGGATGTGGCACTTCGGCATCAGAGAGTAGCTTACAAAGTT

**** ** ******** ***** ************ *************

*ctcf24x* AGTGAGGACATACTTTTCCAACGGACTGATGCAAATATTGTTAGACTGTATTGAAGCCCT

*ctcf24y* AGTGAGGACATACTTTTCCAACGGACTGATGCACACATTGTTAGACTTTATTGAAGCCCT

********************************* * *********** ************

*ctcf24x* GAGTTTTCCTGCAGCAGGATAAGTGAAGGATGAGGGATGTATCTGTCCTTGATCCATGAA

*ctcf24y* GAGTTTTCCTGCAGCAGGATAAGTGAAGGATGAAGGATGTATCTGCCCTTGATCCATGAA

********************************* *********** **************

*ctcf24x* GATTTCTAGCAGTCAGACGAAGCTCCCCGACTGTACCTGTTTAGTATGATGTGGAAAGCT

*ctcf24y* GATTTCTAGCAGTCAGATGAAGCTCCCTGACTGTACCTGTTTAGTATGATGTGGAAAGCT

***************** ********* ********************************

*ctcf24x* GAGGATGAGTGAAAAGGACAACAGGTCTGATGATCTTTGACGAATCTTTGCATTAAGGAT

*ctcf24y* GAGGATGAGTGAAAAGGACAACAAGTCTGATGATCTTTGATGAATCTTTGCATTAAGGAT

*********************** **************** *******************

*ctcf24x* TTGATGGACCTAGTAACCAGACACATTCTAGTGGTAAAGAAGACGTGTCGATCATGCACG

*ctcf24y* TTGATGGACCTAGTAACCAGACAAATTCTAGTGATAAAGAAGACATGTCGGTCATGCACA

*********************** ********* ********** ***** ********

*ctcf24x* TGTCCTTATTGTTGCCTGTTCACAAGTATAATAACAGGCAGTCAGTCCTGAGGAGTGATG

*ctcf24y* TGTTCTTATTGTTGCCCGTTCGCAAGTATATCAACAGG----CAGTCCTGAGGAGTGATG

*** ************ **** ******** ****** ******************

*ctcf24x* GCTGGGTTAAAGCTGTTCACTTTGGGGCTTTTTTTTTTTTCACAATGCATAATATTCTAA

*ctcf24y* GCTGGGTTAAATCTGTTCACTTTGGGGC---TTTTTTTTCCACAATGCATAATATTCTAA

*********** **************** ******** ********************

*ctcf24x* TTTATCTTAGTGCCACGAATATTAGTGGATGCCCCTCAGACAGAACACACTCAAATTAAT

*ctcf24y* TTTATCTTAGTGCCACGAATATTAGTGGATGCCCCTCAGACAGAACACACTCAAATTAAG

***********************************************************

*ctcf24x* GTAACAATAATGAAAGGGGTCTTAAATGATGTAAGTAGCAGTATTGCAAGTATTGATTTT

*ctcf24y* GTAACAATAATGAAAAGGGTCTTAAATGATGTAAGTAGCAGTATTGCAAGTATTGATTTT

*************** ********************************************

*ctcf24x* CACATTTTCACCAGCCGAATCAATCAATTTACAGAATATGATTAATCTTGTTCTGCAAAA

*ctcf24y* CACATTTTCACCAGCCGAAACAATCAATTTAC----------------TGTTCTGCCAAA

******************* ************ ******** ***

*ctcf24x* G-TTTTCATGCTGGTGTAGTAAAAAATATGTGTACTTAATGGTCTGCTCAGATTTCCTCT

*ctcf24y* GTTTTTCATGCTGGTGTAGTAAAAAATATGTGTACTTAATGGTCTGCTCAGATTTTCTCT

* ***************************************************** ****

*ctcf24x* CCAGACTCAAGGTAAACCCTCAGGCAACATTTAAAAAAGGTTTGAGCAGAAGTGTCCAGC

*ctcf24y* CCAGACTCAAGGTAAACCCTCAGGCAACATTTTAAACAGGTTTGAGCAGAAGTGTCCAGC

******************************** *** ***********************

*ctcf24x* TCTAAACACCTCCTCCACTGCAGGTACAGTCTGTGTGTGGGTCCAGGTCCAACTCCGGTC

*ctcf24y* TCTAAACACCTCCTCCACTGTAGGTACAGTCTGTGTCTGGGTCCAGGTCCAACTCCGGTC

******************** *************** ***********************

*ctcf24x* CCAGCTGTGGTTCTGCGGTCCAGCCCAGGATGCTGAGGAGTCACCACTCCCCAGAAAACG

*ctcf24y* CCAGCTGTGGTTCTGCGGTCCAGCCCAGGATGCTGAGGAGTCACCACTCCCCAGAAAATG

********************************************************** *

*ctcf24x* CAGCCAGAGAGAGGAGCCGCGTACGCAACCTGCGACAGGCCTTTCACAGTCTGCAGGTAA

*ctcf24y* CAGCCAGAGAGAGGAGCCGCATACGCAACCTGCGACAGGCCTTTCACAGTCTGCAGGTAA

******************** ***************************************

*ctcf24x* CAACGCAAACGTTTCATTTCCCACCAGCTCATTGAGTTAACAATGAACACAAACTGTCGT

*ctcf24y* CAA-GCAAACGTTTCATTTCCCACCAGCTCACTGAGTTAACAATGAACACAAACTGTTGT

*** *************************** ************************* **

*ctcf24x* AGTTCTCATGTTCTCACTAACTGGATATTTGAATTTAACCACCTGTTTCCTGTTTGTGTG

*ctcf24y* GGTTCTCATGTTCTCACTAACTGGATATTTAAATTTAACCACCTGTTTCCTGTTTGTGTA

***************************** ****************************

*ctcf24x* TTGAATTCAGTCTGATGAATTTGTTTTGCTTTTGCATTGCATTCAGTGAAATGTCACGCC

*ctcf24y* TTGAATTCCGTTTGATGAATTTGTTTTGCTTTTGCA-TGCATTCAGTGAAATGTCATGCC

******** ** ************************ ******************* ***

*ctcf24x* TTTCAAAAGAATAAAAAAAAAATCATCTCTGCTGCTATTTCCTTCTAAAAAGATCATATT

*ctcf24y* TTTCAAAAGAAT--AAAAAAAATAATCTCTGCTGCTATTTCCTTCTAAAAAGAACATATT

************ ********* ***************************** ******

*ctcf24x* ACAGTCGGACCTATAACCATATAGCAACCCATTTGTCAACCAAACACACAGGACGCATAT

*ctcf24y* ACAGTCTGACCTATAACCATATAGCAACCCATTTGTCAACCAAACACACAGGACGCATAT

****** *****************************************************

*ctcf24x* TTGTGTTATGCAAAAAAACCAAACTTTAATGTTAAAGACTTTAATGTTAATAGGCTGAGG

*ctcf24y* TTGTGTTATGCAAA-----------CTACTTTT---GACTTTAATATTAATAAACTGAGG

************** ** * ** ********* ****** ******

*ctcf24x* TCAGTGCAGAGAGCTGAATTAGAGCTGATCCAAACTTGTCCACCCTCCTCCTCTCTCCAC

*ctcf24y* TCAGTGTAGAAAGCTGAATTAGAGCTGATCCAAACTTGTTCACCCTCCTCCTCTCTCAAC

****** *** **************************** ***************** **

*ctcf24x* AGGCTGCTCTGCCTTCAGTCCCGCCTGACACCAAGCTCTCCAAGCTGGATGTTCTGGTGC

*ctcf24y* AGGCTGCTCTGCCTTCAGTCCCGCCTGACACCAAGCTCTCCAAGCTGGATGTTCTGGTGC

************************************************************

*ctcf24x* TGGCTACCAACTACATAGCCTACCTAACAGAGACCCTGGATCAGGGGGGGATTGTGGCAG

*ctcf24y* TGGCTATCAACTACATAGCCTACCTAACAGAGACCCTGGATCAGGGAGGGATTGTGGCAG

****** *************************************** *************

*ctcf24x* AGCACACCCTGTCGTCCAAGCCGGGTGGATACCTGCATCCTGTTAAGGTGAGGCTGGGAG

*ctcf24y* AGCACACCCTGTCGTCCAAGCCGGGTGGATACCTGCATCCTGTTAAGGTGAGGCTGGGAA

***********************************************************

*ctcf24x* C-------------CGTGCAGAGTAACTTACACTGGCTGCTACTGACGTACATTACATGG

*ctcf24y* CCAGTTAGTCTAATCGTGCAGAGCAACTTACACTGTCTGCTACTGATGTACATTACATGG

* ********* *********** ********** *************

*ctcf24x* CCTGCTGGACACATCGATGGTCATTTGGGTGCTACACCTAAAGCCCTGTGTCCCTCTGGG

*ctcf24y* CCTGCTGGACACATCGATGGTCATTTAGGTGCTACA-CTAAAGTCCTGTGTCCCTCTGGG

************************** ********* ****** ****************

*ctcf24x* GGCCTCCCCCAGAAACAGAAGCAGATTTTGAGACAAAGTGTTGTGTAATGTTGTGATATG

*ctcf24y* GGCCGCCCCCAGAAACAGAAGCAGATTTTGAGACAAGTTGTTGTGTAATGTTGTGATATG

**** ******************************* **********************

*ctcf24x* AGAACTGAAAAAAT-TTTTGTGTAGCTGGCTTTTTTTTTTTGTTTAACTATTTTTAGCTT

*ctcf24y* AGTACTGAAAAAATGTTTTGTGTAGCTGGCTTTTTTTCAAGG--TAACTATTTTTAGC--

** *********** ********************** * **************

*ctcf24x* GTTGTAAGTGCATACAACCACCGTTATGATGTTGTGTCACCTATGTTACCTTTAGACAAC

*ctcf24y* -TTGTAAGTGCATACAACTGCTGTTATGATGTTGTGTCACCTATGTTACCTTTAGACAAC

***************** * **************************************

*ctcf24x* TATCTATTT---------------TAGATAATAATCCGTTTTGAGCTTAGCTTGAGCTAG

*ctcf24y* TATCTATTTTAATAATCTAAAAAATAGCTAACTAGCCCTTTTGAGCTTAGCTTGAGCTAG

********* *** *** * ** **********************

*ctcf24x* CTAACCATCTGAAATGTCGTCTGTTGATGAAAATGCCATATAAAAGTCAATTAAACAAAA

*ctcf24y* CTAACCATCTGAAATGTCTTCTGTTGATGAAAATGCCATATAAAAGTCAATTAAACAAAA

****************** *****************************************

*ctcf24x* TATAGCT------------TTGTTATTGTTATATATTATTTTTAATGTATAGTATTATTA

*ctcf24y* TATTGCTTAATGACATGACTTATAATTGTTATATACTATTTTTAATGTATAGTATTATTA

*** *** ** * *********** ************************

*ctcf24x* TATTGACCATGATTTATTTGGTTATAATAGTGCATTTATATACTGCAATGACAAATAATT

*ctcf24y* TATTGACCATGATTTATTTGGCTATAATGGTGCATTTATACACTGCAGTGACAAATAATT

********************* ****** *********** ****** ************

*ctcf24x* TAATATGGCTCTAAAGAATAAGAACATGGTTTCAATTATATTATAATTACTTAGTGACAT

*ctcf24y* TAATATGGCTCTAAAGAATAAGAGCACGA-TTAAATTATTTTATAATTACTTAGTGACAT

*********************** ** * ** ****** ********************

*ctcf24x* TAGTTTCATTGTTATATAGTTGTTACATCATTACTGTTATTTGTTGAGATAACAGCTGTT

*ctcf24y* TAG--TCATTGTTATATAGTTGTTACATCATTACTGTTATTTGTTGAGATAACAGCTGTT

*** *******************************************************

*ctcf24x* TAAAGTGACTGTTTATATAATATTGACGTCATGTGCAGTATTTAATATATTACAGTCTGT

*ctcf24y* TAAAGTGACTGTTTATATAATATTGACGTC--GTGCAGTATTTAATAAATTACAGTCTGT

****************************** *************** ************

*ctcf24x* TCAGTGGAATGTTAAGTACATGTTCTCTGTCAGTTATTACATTATCAACACACGTGACAC

*ctcf24y* TCAGTGGAATGTTAAGTACATGTTCTCTGTCAGTTATTACATTATCAACACACATGACAC

***************************************************** ******

*ctcf24x* ACACACAAAGCTTTTTTAAGGTAATGGTCAAAGTCAGGACATCAGTGATCATCATATCAA

*ctcf24y* ACACACAAAGCTTTTTTAAGCTAATGGTCAAAGTCAGGACATCAGTGATCATCATATTAA

******************** ************************************ **

*ctcf24x* TTAGAAAACATAATCCAATCATTTGAAGTGGGACTTTTGTTTTGTTTGTGCAGTTTTCAC

*ctcf24y* AT----------------------------------------------------------

*

*ctcf24x* TGTTTGTTCCAGCCAAATCGATCCAGAAGATGAAAGCTTTCAGGTGACAGCTCTAAATAG

*ctcf24y* ------------------------------------------------------------

*ctcf24x* GCTGTTATCATTTCGTTTATCATCTTACAGCGAAATATTGAACAAATACTTCTCATTTAG

*ctcf24y* ------------------------------------------------------------

*ctcf24x* GAGGCTTAACAAAGAGGAAATCCTGACTAACAAAAGAGCGATGAAAGCCAACGTAGCCAA

*ctcf24y* -------------------------------------------AAAGCCAACATAGCCAA

********* *******

*ctcf24x* CATTGGTCTCATTGTATCATTTTAAAGAAATG----------------------------

*ctcf24y* CATTGGGCTCATTGTATCATTTTAAAGAAATGACTGGTAACATGACCTCAACACATAAGT

****** *************************

*ctcf24x* -------ACTGAGATGGGCTTTGCTTTGCTGGT-----TGTCTCCTCAGAAGTGGCCCAT

*ctcf24y* CACTTTTACTGAGATGGGCTTTGCTTTGCTGGTTGTCCTGTCTCCTCAGAAGTGGCCCAT

************************** **********************

*ctcf24x* GCGTTCCCTGCTCTACTGTGGCAGCGTGGGAGAGCTGCTCTCAGCCAATCAGATGCCCCC

*ctcf24y* GCGTTCCCTGCTCTACTGTGGCAGCGTGGGAGAGCTGCTCTCAGCCAATCAGATGCCCCC

************************************************************

*ctcf24x* GGCTGGACGAGATGTGACACACCCTCAGACACCCGCCTTAGACGCAGAAAAAGACTGAGA

*ctcf24y* AGCTGGACGAGATGTGACACACCCTCAGACACCCACCTTAGATGCAGAAAAAGATTGA--

********************************* ******* *********** ***

*ctcf24x* TCTTGG

*ctcf24y* ------

**CLUSTAL 2.1 multiple sequence alignment of Cephx1 protein**

Echeneis naucrates_isoform1 MLTGVLVGLVIGGIIFFLVQRNRSQVLKVEDGWWGAGVQPDSEEDVTIRL

Echeneis naucrates_Isoform2 MLTGVLVGLVIGGIIFFLVQRNRSQVLKVEDGWWGAGVQPDSEEDVTIRL

Rachycentron canadum Cephx1x MFTEVLVGLVIGGLIFFLVQRSRNQVLKTEDGWWGAGAPPDGEEDVTIRP

Rachycentron canadum Cephx1y MFTEVLVDLVIGGLIVFLIQRRRNQVLKTEDGWWGAGAPPDGEEEVTIRS

Seriola dumerili MFTEVLLGLVIGGLIFFLVQRSRNQVLKIEDGWWGAGAPPDGEEDVTIRP

Seriola lalandi dorsalis MFIEVLLGLVIGGLIFFLVQRSRNQVLKIEDGWWGAGAPPDGEEDVTIRP

Epinephelus lanceolatus MFTEVLVALVIGGLIFFLVQRSRTQVLKTEDGWWGAGAPPEGGEDVTIRP

Gasterosteus aculeatus MFTEVLVALAIGGLIFFLVQKSRTPVLKTEDGWWGAGAPPDGVEDISIRP

Oryzias latipes MLAELLVAVVTAGLVLFLVQRSRNPVLKAEDGWWGPGVAPHAEEDVSLRP

Danio rerio MYLEVIVALGLGLVVALIFLKKRKTVLKVQDGWWGVGTCPQGPEDDSIRP

Mus musculus MWLELILASVLGFVIYWFVSRDKEETLPLEDGWWGPGSKPSAKEDESIRP

Homo sapiens MWLEILLTSVLGFAIYWFISRDKEETLPLEDGWWGPGTRSAAREDDSIRP

* ::: . : :. : : .* :***** * . . *: ::*

Echeneis naucrates_isoform1 FEVTTSDEELKDLYSRIDQTRPVASLEDSQFNYGFNSQYLQNVISYWRND

Echeneis naucrates_Isoform2 FEVTTSDEELKDLYSRIDQTRPVASLEDSQFNYGFNSQYLQNVISYWRND

Rachycentron canadum Cephx1x FKVTTSNEELQDLYSRIEQTRPVASLEDSQFNYGFNSQYLQKVVSYWRND

Rachycentron canadum Cephx1y FKVTTSNEELQDLYSRIEQMHPVASLEDSQFNYGFNSQYLQKVVSYWRND

Seriola dumerili FRVTTSDEELKDLYSRIDQTRPVASLEDSQFNYGFNSHYLLKVVSYWRND

Seriola lalandi dorsalis FRVTTSDEELKDLYSRIDQTRPVASLEDSQFNYGFNSHYLLKVVSYWRND

Epinephelus lanceolatus FKVSTSDEELEDLYMRIDQTRPVPSLEDSQFHYGFNSQYLQKVVSYWRND

Gasterosteus aculeatus FKVTTSDEELEDLNRRIDQTRPTPSLEDSQFNYGFNSQYLQEVVSYWRND

Oryzias latipes FEVRTSEEELEDLYSRMDRTRPVPSVEDSRFHYGFNSQHLQDVVSYWRKD

Danio rerio FKVETTPEEIEDLHRRLDQTRSFPSLEDSQFNYGFNSKYLEKVVSYWRKD

Mus musculus FKVETSDEEIKDLHQRIDRFRASPPLEGSRFHYGFNSSYLKKVVSFWRNE

Homo sapiens FKVETSDEEIHDLHQRIDKFRFTPPLEDSCFHYGFNSNYLKKVISYWRNE

*.* *: **:.** *::: : ..:*.* *:***** :* .*:*:**::

Echeneis naucrates_isoform1 FDWKRQVDKLNQYPHFKTKIEGIDIHYLHVKPKKVPEGTTAIPLIMVHGW

Echeneis naucrates_Isoform2 FDWKRQVDKLNQYPHFKTKIEGIDIHYLHVKPKKVPEGTTAIPLIMVHGW

Rachycentron canadum Cephx1x FDWRIQVDKLNQYPHFKTKIEGIDIHYLHVKPKKVPEGATAIPLIMVHGW

Rachycentron canadum Cephx1y FDWRRQVDKLNQYPHFKTKIEGIYIHYLHVKPKKVPEGATAIPLIMVQGW

Seriola dumerili FDWGRQVDKLNQYPHFKTKIEGIDIHYLHVKPKKVPDGTTAIPLIMVHGW

Seriola lalandi dorsalis FDWGRQIDKLNQYPHFKTKIEGIDIHYLHVKPKKVPDGTTAIPLIMVHGW

Epinephelus lanceolatus FDWRRQVDKLNQYTHFKTNIEGIDVHYLHVKPKNVPEGATAIPLIMVHGW

Gasterosteus aculeatus FDWRRQVDKLNRYSHFKTNIEGIDVHYLHVKPRNVPEGSTAVPLIMVHGW

Oryzias latipes FDWRRQVHGLNRYPHFRTRIEGIDVHYVHVKPRRVAEGAPALPIILVHGW

Danio rerio FNWGKQLDKLNKYPHFKTKIEGIDIHYVHVKPKNLPEGTRAVPLMMVHGW

Mus musculus FDWRKQVEILNQYPHFKTKIEGLDIHFIHVKPPQLPSGRTPKPLLMVHGW

Homo sapiens FDWKKQVEILNRYPHFKTKIEGLDIHFIHVKPPQLPAGHTPKPLLMVHGW

*:* *:. **:*.**:*.***: :*::**** .:. * . *:::*:**

Echeneis naucrates_isoform1 PGSFYEFYGLIPMLTEPSDP---DDLVFEVVCPS--IPGYGFSEAPHKKG

Echeneis naucrates_Isoform2 PGSFYEFYGLIPMLTEPSDP---DDLVFEVVCPS--IPGYGFSEAPHKKG

Rachycentron canadum Cephx1*x*  PGSFYEFYGLIPLLTEPSDP---DDLVFEVVCPS--IPGYGFSEAPRKKG

Rachycentron canadum Cephx1y PGSFYEFYGLIHLLTEPSDP---DDLVFEVVCPSGFQSKTGNRQEFKLKS

Seriola dumerili PGSFYEFYGLIPLLTEPSDP---DDLVFEVVCPS--IPGYGFSEAPNKKG

Seriola lalandi dorsalis PGSFYEFYGLIPLLTEPSDP---DDLVFEVVCPS--IPGYGFSEAPHKKG

Epinephelus lanceolatus PGSFYEFYRLIPLLTEPSNP---HDLVFEVVCPS--IPGYGFSEAPHKKG

Gasterosteus aculeatus PGSFYEFYGLIPLLTEPSDP---QDLVFEVVCPS--IPGYGFSEAPHKKG

Oryzias latipes PGSFYEFYRLIPLLTEPENP---EEPVFEVVCPS--IPGYGFSEAPHKRG

Danio rerio PGSFYEFYGIISLLTEPSNP---DDITFEVICPS--IPGYGFSEASHKKG

Mus musculus PGSFYEFYKIIPLLTDPKTHGLSDEHVFEVIFPS--IPGYGFSEASSKKG

Homo sapiens PGSFYEFYKIIPLLTDPKNHGLSDEHVFEVICPS--IPGYGFSEASSKKG

******** :* :**:*. .: .***: ** . * : :.

Echeneis naucrates_isoform1 FDSVCAANIFHKLMRRLGFQQFYAHGGDWGWLVTTNMAQLDPKSVKGLHV

Echeneis naucrates_Isoform2 FDSVCAANIFHKLMRRLGFQQFYAHGGDWGWLVTTNMAQLDPKSVKGLHV

Rachycentron canadum Cephx1x FDSVCAANIFNKLMKRLGFQQFYAHGGDWGWLVTTNMAQLDPKSVKGLHV

Rachycentron canadum Cephx1y FDSVCAANIFHKLMKRLGFQQFYAHRGDWGWLVTTNMAQLDPKSVKGLHV

Seriola dumerili FDSVCAANIFHKLMKRLGFQQFYAHGGDWGWLVTTNMAQLEPKSVKGLHV

Seriola lalandi dorsalis FDSVCAANIFHKLMKRLGFQQFYAHGGDWGWLVTTNMAQLDPKSVKGLHV

Epinephelus lanceolatus FDSVCAAHIFHKLMKRLGFQQFYAHGGDWGWLVTTNMAQLEPKTVKGLHV

Gasterosteus aculeatus FDSVCAARIFHKLMKRLGFHKFYAHGGDWGWLVTTNMAQLQPGVVKGLHV

Oryzias latipes FNSVCAANVFQKLMKRLGFQTFFAHGGDWGWQITTNMAQLDPGSVRGLHL

Danio rerio FDTVCAAHIFNKLMKRLGFTQYYVQGGDWGSMITTNMAQLEPNAVKGLHI

Mus musculus LNSVATARIFYKLMSRLGFQKFYIQGGDWGSLICTNIAQMVP--------

Homo sapiens FNSVATARIFYKLMLRLGFQEFYIQGGDWGSLICTNMAQLVPSHVKGLHL

:::*.:*.:* *** **** :: : **** : **:**: *

Echeneis naucrates_isoform1 NFAPPSQHSVHMLLSIMLGRRFPKLFGFTDVDIQRLYPCKEKLLVEPIKE

Echeneis naucrates_Isoform2 NFAPPSQHSVHMLLSIMLGRRFPKLFGFTDVDIQRLYPCKEKLLVEPIKE

Rachycentron canadum Cephx1x NFAPPSKPGLPMALSIMLGRHFPKLFGFTDVDIQRLYPCTEKLVVESIKE

Rachycentron canadum Cephx1y NFAPPSKPGLPMALSIMLGRHFPKLFGFTVVDIQRLYPCTEKLVVESIKE

Seriola lalandi dorsalis NFAPPSKPGLPMTLSILLGRRFPKLFGFTDVDIQRLYPCMEKLVVESIKE

Epinephelus lanceolatus NFAPPSKPGLPMTLSILLGRRFPKLFGFTDVDIQRLYPCMEKLVVESIKE

Gasterosteus aculeatus NFAPPSKPGLTMVLSIMLGRRFPKLFGFTDMDIQHLYPAMERQVVEPIKE

Gasterosteus aculeatus NFAPNSRPGLSMVLSVMLGRRFPKMFGFTDFDVRRLFPCVDKLVVEAVKE

Oryzias latipes SFAPPSKPGLLVALSLMLGRLFPTLFGFTEADRRLLYPCTEKLLLESMKE

Danio rerio NFAPPAQGSVLMGLSLIFGRRFPKLFGFTEHDVKRLFPCMDKLVVDALRE

Mus musculus --------KNIYSLTPLLGQRFGRFLGYTEKDLELLYPFKEKVFYNIMRE

Homo sapiens NMA--LVLSNFSTLTLLLGQRFGRFLGLTERDVELLYPVKEKVFYSLMRE

*: ::*: * ::* * * . *:* :: . . ::*

Echeneis naucrates_isoform1 SGYMHIQATKPDTVGRGLNDSPVGLAAYILEKFSTWTNVDFRDLDDGGLT

Echeneis naucrates_Isoform2 SGYMHIQATKPDTVGRGLNDSPVGLAAYILEKFSTWTNVDFRDLDDGGLT

Rachycentron canadum Cephx1x SGYMHIQATKPDTVGRGLNDSPVGLAAYILEKFSTWTNRDFRDLEDGGLT

Rachycentron canadum Cephx1y SGYMHIQATKPDTVGRGLNDSTVGLAAYILEKFSTWTNRDFRDLEDGGLT

*Seriola dumerili* SGYMHIQATKPDTVGRGLNDSPVGLAAYILEKFSTWTDRDFRNLEDGGLT

*Seriola lalandi dorsalis* SGYMHIQATKPDTVGRGLNDSPVGLAAYILEKFSTWTDRDFRNLEDGGLT

*Epinephelus lanceolatus* SGYMHIQATKPDTVGRGLNDSPVGLAAYILEKFSTWTSRDFRNLEDGGLT

*Gasterosteus aculeatus* TGYMHIQATKPDTAGRGLNDSPVGLAAYILEKFSTWTSPDFRNLEDGGLT

*Oryzias latipes* SGYLHIQATKPDTAGRALNDSPVGLAAYILEKFSTWTNQNFRDLEDGGLT

*Danio rerio* TGYMHIQATKPDTAGRGLNDSPVGLAAYILEKFSTWTDPEFRKLEDGGLE

*Mus musculus* SGYLHIQATKPDTVGCALNDSPVGLAAYILEKFSTWTKSEYCELEDGGLE

*Homo sapiens* SGYMHIQCTKPDTVGSALNDSPVGLAAYILEKFSTWTNTEFRYLEDGGLE

:**:***.*****.* .****.***************. :: *:****

*Echeneis naucrates_isoform1* RKFSLDDLLTNVMIYWVSGCIISSMRFYKENFGK-GLNQPHSKIPVYVPT

*Echeneis naucrates_Isoform2* RKFSLDDLLTNVMIYWVSGCIISSMRFYKENFGK-GLNQPHSKIPVYVPT

Rachycentron canadum Cephx1x RNFSLDDLLTNVMIYWVSGCIISSMRFYKENFGK-GLDQPHSKIPVYVPT

Rachycentron canadum Cephx1y R------------------------------------------IPVYVPT

*Seriola dumerili* RKFSLDDLLTNVMIYWVSGCIISSMRFYKENFGK-GLDQPHSKIPVYVPT

*Seriola lalandi dorsalis* RKFSLDDLLTNVMIYWVSGCIISSMRFYKENFGK-GLDQPHSKIPVYVPT

*Epinephelus lanceolatus* RKFSLDDLLTNVMIYWTSGCIISSMRFYKENFGK-GLNQPHAKIPVYVPT

*Gasterosteus aculeatus* RKFSLDDLLTNVMIYWTSGCIVSSMRFYKENFGR-GLNQPHSRVPVYVPT

*Oryzias latipes* RKFSLDDLLTNVMIYWTSGCITSSMRFYKENFGK-GLDQPHSRIPVNVPT

*Danio rerio* RKYSIDDLLTNVMIYWTSGCIISSMRFYKENFGK-GLNQPHAKLPVHVPT

*Mus musculus*  RKFSLEDLLTNIMIYWTTGTIVSSQRFYKENLGQGVMVHRHEGMKVFVPT

*Homo sapiens* RKFSLDDLLTNVMLYWTTGTIISSQRFYKENLGQGWMTQKHERMKVYVPT

* : * ***

Echeneis naucrates_isoform1 GFACFPNELMHTPKLWVQQKYRKLLTYTPMAHGGHFAAMEEPSLMAEDIQ

Echeneis naucrates_Isoform2 GFACFPNELMHTPKLWVQQKYRKLLTYTPMAHGGHFAAMEEPSLMAEDIQ

Rachycentron canadum Cephx1x GFACFPNELMHTPKLWVQQKYRKLLTYTPMSRGGHFAAMEEPQLMAEDIQ

Rachycentron canadum Cephx1y GFSCFPNELMHTPKLWVQRKYRKLLTYTPMVRGGHFAAMEEPQLMAKDIQ

Seriola dumerili GFACFPNELMHTPKLWVTQKYRKLVTYTPMVRGGHFAAMEEPQLMAEDIQ

Seriola lalandi dorsalis GFACFPNELMHTPKLWVTQKYRKLATYTPMVRGGHFAAMEEPQLMAEDIQ

Epinephelus lanceolatus GFACFPDELMHTPQLWVKQKYRKLVTFTPMPRGGHFAAMEEPQLMAEDIQ

Gasterosteus aculeatus GYACFPNELMHTPKLWVEHKYRKLLTYSPMARGGHFAAMEEPRLMAEDIQ

Oryzias latipes GFACFPNDVMHSPKLWVKQKYRNLIRYTPMPRGGHFAAMEEPQLMAEDIQ

Danio rerio GVASFPNELLHTPKLWVTDKYRNLKSYTPIARGGHFAAMEEPQLLAEDVQ

Mus musculus GYSAFPSEILHAPEKWVKVKYPKLISYSYMERGGHFAAFEEPKLLAQDIR

Homo sapiens GFSAFPFELLHTPEKWVRFKYPKLISYSYMVRGGHFAAFEEPELLAQDIR

* :.** :::*:*: ** ** :* :: : :******:*** *:*:*::

Echeneis naucrates_isoform1 KFTKTVEEKKK-

Echeneis naucrates_Isoform2 KFTKTVEEKKK-

Rachycentron canadum Cephx1x KFTKTVEKKKK-

Rachycentron canadum Cephx1y KFTKTVEKKKK-

Seriola dumerili KFTKTVEKKKQ-

Seriola lalandi dorsalis KFTKTVEKKKQ-

Epinephelus lanceolatus NFTKTVEKKIK-

Gasterosteus aculeatus NFTKTVEKKKK-

Oryzias latipes EFTKTVEKMIKL

Danio rerio NFVKIVENRKRK

Mus musculus KFVSLAELQ---

Homo sapiens KFLSVLERQ---

:* . *
