# Supplementary material for: Exploring the cobia (Rachycentron canadum) genome: unveiling putative male heterogametic regions and identification of sex-specific markers
Source: Gigascience. 2024 Jul 12;13:giae034. doi: 10.1093/gigascience/giae034 (PMC11240236; doi:10.1093/gigascience/giae034)
Supplement: giae034_Response_to_Reviewer_Comments_Original_Submission [file giae034_response_to_reviewer_comments_original_submission.pdf]

Author's Response To Reviewer Comments

Date: 20 Apr 2024
Xueyan Shen
Subject: Response to the referees

Dear Dr Zhou,
First of all, thank you for your kind assistance in the process of publishing “Genomic investigation of the cobia (Rachycentron canadum) reveals putative male heterogametic regions on chromosome 18" to GigaScience.
The title has now been amended per the suggestions of reviewers: “Exploring the cobia (Rachycentron canadum) genome: unveiling putative male heterogametic regions and identification of sex-specific markers". We would like to thank the three reviewers for their valuable and constructive criticisms. We have revised the MS based on their requests and suggestions. We feel that the revised version shows marked improvements compared to the original submission and we hope that the editor and reviewers share our opinion.

Detailed response to the specific questions (line numbers refer to the revised MS):

Reviewer Reports
Reviewer #1: Shen et al. present their results on the identification of a sex locus in cobia. They compiled an impressive amount of data. Assembled male and female genomes using short stLFR read data (which is similar to 10XGenomics linked reads). Re-sequenced 91 individual fish samples. Analysis is straight forward and follows a clear path down to the discovery of two genes that might be candidates for novel master sex determination genes. PCR sexing assays were designed and enabled sexing in different populations of cobia. The manuscript is scientifically sound, well written and in principle deserves publication in Gigascience. I have just one major comment:

Major comments:
The genome assemblies from stLFR data have relatively low continuity (contig N50 only 80 - 86Kbp). This is not state-of-the-art anymore. Seemingly, there were some issues with the stLFR short read assemblies at least for the SD region and the authors also performed PACBIO HIFI data to compensate for this.
Why do they not publish the full genome assembly based on the long reads? They just used the PACB data to get a better resolution of the sex-specific region and chromosome, but it is unclear if this chromosome is completely phased or a chimera of Y and X sequences.
It should be possible to create a fully phased diploid genome assembly from the data the authors already have. This would provide full separation of the X and Y chromosomes. I think the authors should not miss the opportunity to publish a state-of-the-art assembly.
Reply: We greatly appreciate the reviewer's insightful comments and suggestions. Indeed, we acknowledge the challenges posed by the continuity of the genome assemblies derived from stLFR data, and therefore had closed gaps in the sex-specific region of MChr18 with PACBIO (known for its highly accurate long-read data surpassing 99.9% accuracy). As per the suggestion, we have now completed the full assembly of the male genome with PACBIO and and submitted the data to GigaDB. We will also make it publicly available by submitting it to the NCBI once the Manuscript is accepted. This indeed increased the quality of the publication, in addition to the full genome annotation (first for the species), syntheny study of close related species, re-sequencing of 91 individuals and GWAS which allowed the identification of sex-related region in MChr18, identification of two putative novel sex-genes, development of PCR sex-markers; and proof-of-concept of conservation of this genome feature across geographically distinct populations of Panama, Brazil, Australia and Japan.
In regard to the suggestion to create a fully diploid genome with separation of X and Y chromosomes, this spawns beyond the objectives of the research, and we have toned down on the X and Y Chr (as suggested by other reviewers). Additionally, the title has been revised to: “Exploring the cobia (Rachycentron canadum) genome: Unveiling putative male heterogametic regions and identification sex-specific markers”.
The comprehensive work done by the cobia genome consortium which culminated in the work presented here, and which involved several institutions and authors from across the globe where this large marine

species is found, and the funding for this research have come to an end, and it will not be possible to continue with further analysis. The legacy and breadth of this work on cobia Rachycentron canadum, which lacks on genomic resources, and the vast amount of sequencing data will be made publicly accessible for future works shall funding becomes available.
Minor comments:
L.246: The authors state that they have PACBIO HIFI reads, while in the Methods section they talk about sequencing CLR mode not HIFI (L. 538). This needs to be clarified.
Reply: Thanks to the reviewer for pointing out this oversight. We have now made the necessary correction.
Line538 “The library was sequenced using the PacBio Sequel II System with HiFi sequencing modes.”

L.411: “it has known” should be “it is known”
Reply: Changed as suggested by the reviewer.

I can see that read data has been submitted to NCBI.
I can not access the link “https://pan.genomics.cn/ucdisk/s/36Fjqi”
Reply: Thanks for the Reviewer’s feedback. We have previously submitted all the data to NCBI and GigaDB as requested by the GigaDB Team. They have confirmed this via email “It appears everything required for peer review is available from the following locations, and your manuscript has therefore been forwarded to the next stage of the review process”.

I can not access data provided by Gigascience “ftp://user92@parrot.genomics.cn”
Reply: Same reply as above.

Reviewer #2: The manuscript titled “Genomic investigation of the cobia (Rachycentron canadum) reveals putative male heterogametic regions on chromosome 18” presents a significant study in the field of genomic research. It focuses on the cobia fish, highlighting its unique sexual dimorphism in growth rate and the unknown molecular mechanisms of sex determination in this species. The research includes the assembly of high-quality chromosome-level annotated male and female cobia genomes, revealing three short male-specific regions on male chromosome 18, suggesting a potential XX/XY mode of sex determination. The study identifies two genes, ephx1 and tcf24, as being crucial in the sex determination process and demonstrates their specific expression in male tissues. The findings offer valuable insights for future studies on cobia population structure, conservation, and evolutionary history.

A scientific Issue noted in the manuscript is the interpretation of the genomic data to draw the conclusion of sex chromosome identification and male heterogametic sex determination system (XX/XY) for cobia. Sex chromosome have clear definition that hardly to identify merely by genomic data. Again, it is dangerous to jump to the conclusion of sex determination system (XY or ZW) using genomic data without any population experiment validation. I would like to suggestion author should weaken their interpretation to draw such a conclusion.
Reply: We thank the reviewer for the valuable feedback. We recognize the crucial importance of precision in interpreting sex chromosomes identification and understanding sex determination systems. We acknowledge the inherent challenges in defining sex chromosomes only through genomic data. Given the focus of this current work on identifying sex-associated regions and sex-related markers, we have tempered our interpretation regarding sex chromosome identification and male heterogametic sex determination system (XX/XY) for cobia (Lines 49, 58, 222, 400, 409, 510).
Furthermore, we have revised the title to: “Exploring the cobia (Rachycentron canadum) genome: Unveiling putative male heterogametic regions and identification of sex-specific markers”.

Also, the manuscript contains some minor issues such as inconsistent tense usage, occasional awkward phrasing, and punctuation errors. These do not detract significantly from the overall quality of the work but should be addressed in a minor revision.

Here are some examples, but I suggested that author should address issues through the whole manuscript:

Inconsistent Tense Usage:
Example: "grow faster than males both in body length and weight creating considerable differences

between sexes in which females can be double the size of males at similar developmental stages".
Suggested correction: This sentence should maintain consistent tense usage. For instance, "females grow faster than males in both body length and weight, creating considerable differences between the sexes. At similar developmental stages, females can be double the size of males."
Reply: Changed as suggested by reviewer. Lines76-78.

Awkward Phrasing:
Example: "About 11.08% and 11.55% of the assembled male and female genome was annotated and identified as repetitive elements".
Suggested correction: The phrase could be more concise and clear. For example, "Approximately 11.08% of the male genome and 11.55% of the female genome were annotated as repetitive elements."
Reply: Changed as suggested by reviewer. Line 173 to 175.

Punctuation Errors:
Example: "Using the male genome as reference we employed two different strategies to identify the sex-specific region(s) in cobia".

Suggested correction: A comma is needed after 'reference' for better readability.
Reply: Thanks for the suggestion. We found there is already a comma after ‘reference’ in this sentence. Line 625.

Example: "the latter observation (17% intersex) being attributed to increased levels of endocrine disrupting compounds (EDC) from industrial and agricultural pollutants in local waterways".
Suggested correction: This sentence could be restructured for clarity. For instance, "The observation of 17% intersex individuals is attributed to increased levels of endocrine disrupting compounds (EDC) from industrial and agricultural pollutants in local waterways."
Reply: Changed as suggested by reviewer. Line 442.

Sentence Fragment:
Example: "Consequently it's improbable that these regions play an important role in sex determination".
Suggested correction: This fragment could be integrated into a complete sentence for better flow. "Consequently, we conclude that it is improbable these regions play an important role in sex determination."
Reply: Changed as suggested by reviewer. Lines 239 to 240.

Reviewer #3: In this manuscript, Shen and colleagues reported the male and female genomic resources of cobia Rachycentron canadum, and narrowed down potential male-specific genomic region via resequencing male and female individuals. The authors also validated the sex-specific DNA markers with individuals coming from other populations. The introduction is adequate, and overall the manuscript is also well written. I have two minor comments for the authors to consider to improve the manuscript.

1) Title: The current title is "Genomic investigation of the cobia (Rachycentron canadum) reveals putative male heterogametic regions on chromosome 18". My understanding is that there were previous studies working on the karyotypes of Rachycentron canadum based on shape of metaphase chromosomes. Should I get it correctly, the authors assigned the number of chromosomes based on descending order of sizes (Table S4). Thus chromosome 18 named in previous karyotype study and the chromosome 18 named in this study can not be judged as if they are the same or not. In this case, "on chromosome 18" should be removed from the title.
Reply: The title has now been revised to: “Exploring the cobia (Rachycentron canadum) genome: Unveiling putative male heterogametic regions and identification of sex-specific markers”. In addition, we have incorporated pertinent information into the figure legend of Figure 1. Line 180 to 182 “In the male genome, chromosome numbering is organized in descending order according to chromosome assembly size. Whereas the female genome's chromosome numbering follows a one-to-one syntenic relationship with the male genome.”
2) GWAS and Fst analyses - The authors also detected potential association signals of GWAS on three other chromosomes (MChr4, 5, 17). The authors wrote in line 228-231 as follows: "a more detailed examination revealed that the SNP genotypes within these regions did not consistently exhibit heterozygosity in males

and homozygosity in females. Furthermore, the resequencing data showed comparable coverage of these regions in both males and females". I think the authors should at least: i) show all these results in the supplementary information, and ii) at least write in the main text what protein coding genes are included in the genomic regions.

Reply: We sincerely appreciate the valuable suggestions provided by the reviewer. Within the Supplementary Table file (in Excel format), we have incorporated Supplementary Table S12 (GWAS-detected suggestive signals of sex association at Chr4, Chr5, and Chr17). Additionally, we have also included the text 'Supplementary Table S12' in the main paper, line 231-233: 'a more detailed examination revealed that the SNP genotypes within these regions did not consistently exhibit heterozygosity in males and homozygosity in females (Supplementary Table S12).' Consequently, the original Supplementary Table S12 and Table S13 have been re-designated as Table S13 and Table S14, respectively, in both the Supplementary Tables (Excel files) and the main paper. Regarding the request for supplementary information pertaining to the statement 'Furthermore, the resequencing data showed comparable coverage of these regions in both males and females', we regret to inform that the original "bam files" utilized for calculating coverage of each sample have been removed due to prolonged non-usage, as all analyses were conducted between 2021 and 2022. Should the reviewer deem additional proof necessary, we are open to removing the sentence accordingly.

Furthermore, as requested by the reviewer, we have elaborated in the main paper on the inclusion of protein-coding genes within the three genomic areas (MChr4, MChr5, and MChr17). Lines 235 to 240, "Additionally, three genes were identified on MChr4: mdn1 (Midasin AAA ATPase 1), trm6 (tRNA Methyltransferase 6 Non-Catalytic Subunit), and fermt1 (FERM Domain Containing Kindlin 1), while no genes were detected on Chr5. On Chr17, ostm1 (Osteoclastogenesis Associated Transmembrane Protein 1) was identified. However, these genes have not previously been reported to have a functional role in sexual development and function."
