# Supplementary material for: Exploring the cobia (Rachycentron canadum) genome: unveiling putative male heterogametic regions and identification of sex-specific markers
Source: Gigascience. 2024 Jul 12;13:giae034. doi: 10.1093/gigascience/giae034 (PMC11240236; doi:10.1093/gigascience/giae034)
Supplement: giae034_Reviewer_1_Report_Original_Submission — Chris Armit -- 2/7/2024 Reviewed [file giae034_reviewer_1_report_original_submission.pdf]

Reviewer Report

Title: Exploring the cobia (Rachycentron canadum)

genome: Unveiling putative male heterogametic regions and identification sex-specific markers

Version: Original Submission **Date:** 1/2/2024

Reviewer name: Heiner Kuhl

Reviewer Comments to Author:

Shen et al. present their results on the identification of a sex locus in cobia. They compiled an impressive amount of data. Assembled male and female genomes using short stLFR read data (which is similar to 10XGenomics linked reads). Re-sequenced 91 individual fish samples. Analysis is straight forward and follows a clear path down to the discovery of two genes that might be candidates for novel master sex determination genes. PCR sexing assays were designed and enabled sexing in different populations of cobia. The manuscript is scientifically sound, well written and in principle deserves publication in Gigascience. I have just one major comment:
Major comments
The genome assemblies from stLFR data have relatively low continuity (contig N50 only 80 - 86Kbp). This is not state-of-the-art anymore. Seemingly, there were some issues with the stLFR short read assemblies at least for the SD region and the authors also performed PACBIO HIFI data to compensate for this.
Why do they not publish the full genome assembly based on the long reads? They just used the PACB data to get a better resolution of the sex-specific region and chromosome, but it is unclear if this chromosome is completely phased or a chimera of Y and X sequences.
It should be possible to create a fully phased diploid genome assembly from the data the authors already have. This would provide full separation of the X and Y chromosomes. I think the authors should not miss the opportunity to publish a state-of-the-art assembly.
Minor comments:
L.246: The authors state that they have PACBIO HIFI reads, while in the Methods section they talk about sequencing CLR mode not HIFI (L. 538). This needs to be clarified.
L.411: "it has known" should be "it is known"
I can see that read data has been submitted to NCBI.
I can not access the link "https://pan.genomics.cn/ucdisk/s/36Fjqi"
I can not access data provided by Gigascience "ftp://user92@parrot.genomics.cn"

Methods

Are the methods appropriate to the aims of the study, are they well described, and are necessary controls included? Choose an item.

Conclusions

Are the conclusions adequately supported by the data shown? Choose an item.

Reporting Standards

Does the manuscript adhere to the journal's guidelines on minimum standards of reporting? Choose an item.

Choose an item.

Statistics

Are you able to assess all statistics in the manuscript, including the appropriateness of statistical tests used? Choose an item.

Quality of Written English

Please indicate the quality of language in the manuscript: Choose an item.

Declaration of Competing Interests

Please complete a declaration of competing interests, considering the following questions:

- Have you in the past five years received reimbursements, fees, funding, or salary from an organisation that may in any way gain or lose financially from the publication of this manuscript, either now or in the future?
- Do you hold any stocks or shares in an organisation that may in any way gain or lose financially from the publication of this manuscript, either now or in the future?
- Do you hold or are you currently applying for any patents relating to the content of the manuscript?
- Have you received reimbursements, fees, funding, or salary from an organization that holds or has applied for patents relating to the content of the manuscript?
- Do you have any other financial competing interests?
- Do you have any non-financial competing interests in relation to this paper?

If you can answer no to all of the above, write 'I declare that I have no competing interests' below. If your reply is yes to any, please give details below.

I declare that I have no competing interests.

I agree to the open peer review policy of the journal. I understand that my name will be included on my report to the authors and, if the manuscript is accepted for publication, my named report including any attachments I upload will be posted on the website along with the authors' responses. I agree for my report to be made available under an Open Access Creative Commons CC-BY license (http://creativecommons.org/licenses/by/4.0/). I understand that any comments which I do not wish to

be included in my named report can be included as confidential comments to the editors, which will not be published.

Choose an item.

To further support our reviewers, we have joined with Publons, where you can gain additional credit to further highlight your hard work (see: https://publons.com/journal/530/gigascience). On publication of this paper, your review will be automatically added to Publons, you can then choose whether or not to claim your Publons credit. I understand this statement.

Yes Choose an item.
