# Supplementary material for: Exploring the cobia (Rachycentron canadum) genome: unveiling putative male heterogametic regions and identification of sex-specific markers
Source: Gigascience. 2024 Jul 12;13:giae034. doi: 10.1093/gigascience/giae034 (PMC11240236; doi:10.1093/gigascience/giae034)
Supplement: giae034_Reviewer_2_Report_Original_Submission — Shangpeng Sun -- 2/26/2024 Reviewed [file giae034_reviewer_2_report_original_submission.pdf]

Reviewer Report

Title: Exploring the cobia (Rachycentron canadum)

genome: Unveiling putative male heterogametic regions and identification sex-specific markers

Version: Original Submission **Date:** 2/1/2024

Reviewer name: shijun xiao

Reviewer Comments to Author:

The manuscript titled "Genomic investigation of the cobia (Rachycentron canadum) reveals putative male heterogametic regions on chromosome 18" presents a significant study in the field of genomic research. It focuses on the cobia fish, highlighting its unique sexual dimorphism in growth rate and the unknown molecular mechanisms of sex determination in this species. The research includes the assembly of high-quality chromosome-level annotated male and female cobia genomes, revealing three short male-specific regions on male chromosome 18, suggesting a potential XX/XY mode of sex determination. The study identifies two genes, ephx1 and tcf24, as being crucial in the sex determination process and demonstrates their specific expression in male tissues. The findings offer valuable insights for future studies on cobia population structure, conservation, and evolutionary history.

A scientific issue noted in the manuscript is the interpretation of the genomic data to draw the conclusion of sex chromosome identification and male heterogametic sex determination system (XX/XY) for cobia. Sex chromosome have clear definition that hardly to identify merely by genomic data. Again, it is dangerous to jump to the conclusion of sex determination system (XY or ZW) using genomic data without any population experiment validation. I would like to suggestion author should weaken their interpretation to draw such a conclusion.

Also, the manuscript contains some minor issues such as inconsistent tense usage, occasional awkward phrasing, and punctuation errors. These do not detract significantly from the overall quality of the work but should be addressed in a minor revision.

Here are some examples, but I suggested that author should address issues through the whole manuscript:

Inconsistent Tense Usage:

Example: "grow faster than males both in body length and weight creating considerable differences between sexes in which females can be double the size of males at similar developmental stages"â€‹â€‹.

Suggested correction: This sentence should maintain consistent tense usage. For instance, "females grow faster than males in both body length and weight, creating considerable differences between the sexes. At similar developmental stages, females can be double the size of males."

Awkward Phrasing:

Example: "About 11.08% and 11.55% of the assembled male and female genome was annotated and identified as repetitive elements"â€‹â€‹.

Suggested correction: The phrase could be more concise and clear. For example, "Approximately 11.08% of the male genome and 11.55% of the female genome were annotated as repetitive elements."
Punctuation Errors:
Example: "Using the male genome as reference we employed two different strategies to identify the sex-specific region(s) in cobia"â€‹â€‹.
Suggested correction: A comma is needed after 'reference' for better readability.
Example: "the latter observation (17% intersex) being attributed to increased levels of endocrine disrupting compounds (EDC) from industrial and agricultural pollutants in local waterways"â€‹â€‹.
Suggested correction: This sentence could be restructured for clarity. For instance, "The observation of 17% intersex individuals is attributed to increased levels of endocrine disrupting compounds (EDC) from industrial and agricultural pollutants in local waterways."
Sentence Fragment:
Example: "Consequently it's improbable that these regions play an important role in sex determination"â€‹â€‹.
Suggested correction: This fragment could be integrated into a complete sentence for better flow. "Consequently, we conclude that it is improbable these regions play an important role in sex determination."

Methods

Are the methods appropriate to the aims of the study, are they well described, and are necessary controls included? Choose an item.

Conclusions

Are the conclusions adequately supported by the data shown? Choose an item.

Reporting Standards

Does the manuscript adhere to the journal's guidelines on minimum standards of reporting? Choose an item.

Choose an item.

Statistics

Are you able to assess all statistics in the manuscript, including the appropriateness of statistical tests used? Choose an item.

Quality of Written English

Please indicate the quality of language in the manuscript: Choose an item.

Declaration of Competing Interests

Please complete a declaration of competing interests, considering the following questions:

- Have you in the past five years received reimbursements, fees, funding, or salary from an organisation that may in any way gain or lose financially from the publication of this manuscript, either now or in the future?
- Do you hold any stocks or shares in an organisation that may in any way gain or lose financially from the publication of this manuscript, either now or in the future?
- Do you hold or are you currently applying for any patents relating to the content of the manuscript?
- Have you received reimbursements, fees, funding, or salary from an organization that holds or has applied for patents relating to the content of the manuscript?
- Do you have any other financial competing interests?
- Do you have any non-financial competing interests in relation to this paper?

If you can answer no to all of the above, write 'I declare that I have no competing interests' below. If your reply is yes to any, please give details below.

I declare that I have no competing interests

I agree to the open peer review policy of the journal. I understand that my name will be included on my report to the authors and, if the manuscript is accepted for publication, my named report including any attachments I upload will be posted on the website along with the authors' responses. I agree for my report to be made available under an Open Access Creative Commons CC-BY license (http://creativecommons.org/licenses/by/4.0/). I understand that any comments which I do not wish to be included in my named report can be included as confidential comments to the editors, which will not be published.

Choose an item.

To further support our reviewers, we have joined with Publons, where you can gain additional credit to further highlight your hard work (see: https://publons.com/journal/530/gigascience). On publication of this paper, your review will be automatically added to Publons, you can then choose whether or not to claim your Publons credit. I understand this statement.

Yes Choose an item.
