# Supplementary material for: Exploring the cobia (Rachycentron canadum) genome: unveiling putative male heterogametic regions and identification of sex-specific markers
Source: Gigascience. 2024 Jul 12;13:giae034. doi: 10.1093/gigascience/giae034 (PMC11240236; doi:10.1093/gigascience/giae034)
Supplement: giae034_Reviewer_3_Report_Original_Submission — Michael Pound -- 3/30/2024 Reviewed [file giae034_reviewer_3_report_original_submission.pdf]

Reviewer Report

Title: Exploring the cobia (Rachycentron canadum)

genome: Unveiling putative male heterogametic regions and identification sex-specific markers

Version: Original Submission **Date:** 2/4/2024

Reviewer name: Jerome Hui

Reviewer Comments to Author:

In this manuscript, Shen and colleagues reported the male and female genomic resources of cobia Rachycentron canadum, and narrowed down potential male-specific genomic region via resequencing male and female individuals. The authors also validated the sex-specific DNA markers with individuals coming from other populations. The introduction is adequate, and overall the manuscript is also well written. I have two minor comments for the authors to consider to improve the manuscript.
1) Title: The current title is "Genomic investigation of the cobia (Rachycentron canadum) reveals putative male heterogametic regions on chromosome 18". My understanding is that there were previous studies working on the karyotypes of Rachycentron canadum based on shape of metaphase chromosomes. Should I get it correctly, the authors assigned the number of chromosomes based on descending order of sizes (Table S4). Thus chromosome 18 named in previous karyotype study and the chromosome 18 named in this study can not be judged as if they are the same or not. In this case, "on chromosome 18" should be removed from the title.
2) GWAS and Fst analyses - The authors also detected potential association signals of GWAS on three other chromosomes (MChr4, 5, 17). The authors wrote in line 228-231 as follows: "a more detailed examination revealed that the SNP genotypes within these regions did not consistently exhibit heterozygosity in males and homozygosity in females. Furthermore, the resequencing data showed comparable coverage of these regions in both males and females". I think the authors should at least: i) show all these results in the supplementary information, and ii) at least write in the main text what protein coding genes are included in the genomic regions.

Methods

Are the methods appropriate to the aims of the study, are they well described, and are necessary controls included? Choose an item.

Conclusions

Are the conclusions adequately supported by the data shown? Choose an item.

Reporting Standards

Does the manuscript adhere to the journal’s guidelines on minimum standards of reporting? Choose an item.

Choose an item.

Statistics

Are you able to assess all statistics in the manuscript, including the appropriateness of statistical tests used? Choose an item.

Quality of Written English

Please indicate the quality of language in the manuscript: Choose an item.

Declaration of Competing Interests

Please complete a declaration of competing interests, considering the following questions:

- Have you in the past five years received reimbursements, fees, funding, or salary from an organisation that may in any way gain or lose financially from the publication of this manuscript, either now or in the future?
- Do you hold any stocks or shares in an organisation that may in any way gain or lose financially from the publication of this manuscript, either now or in the future?
- Do you hold or are you currently applying for any patents relating to the content of the manuscript?
- Have you received reimbursements, fees, funding, or salary from an organization that holds or has applied for patents relating to the content of the manuscript?
- Do you have any other financial competing interests?
- Do you have any non-financial competing interests in relation to this paper?

If you can answer no to all of the above, write 'I declare that I have no competing interests' below. If your reply is yes to any, please give details below.

I declare that I have no competing interests.

I agree to the open peer review policy of the journal. I understand that my name will be included on my report to the authors and, if the manuscript is accepted for publication, my named report including any attachments I upload will be posted on the website along with the authors' responses. I agree for my report to be made available under an Open Access Creative Commons CC-BY license (http://creativecommons.org/licenses/by/4.0/). I understand that any comments which I do not wish to be included in my named report can be included as confidential comments to the editors, which will not be published.

Choose an item.

To further support our reviewers, we have joined with Publons, where you can gain additional credit to further highlight your hard work (see: https://publons.com/journal/530/gigascience). On publication of this paper, your review will be automatically added to Publons, you can then choose whether or not to claim your Publons credit. I understand this statement.

Yes Choose an item.
